# Supplementary material for: Molecular evolutionary trends and feeding ecology diversification in the Hemiptera, anchored by the milkweed bug genome
Source: Genome Biol. 2019 Apr 2;20:64. doi: 10.1186/s13059-019-1660-0 (PMC6444547; doi:10.1186/s13059-019-1660-0)
Supplement: Supplementary file 1 — Supplementary notes, figures, and small tables. (PDF 6142 kb) [file 13059_2019_1660_MOESM1_ESM.pdf]

Milkweed Bug (*Oncopeltus fasciatus*)

Genome Consortium

[Logo by Chiaki Ueda]

Supplementary Information

Table of Contents

1. Genome and transcriptome sequencing and assembly....4
1.1 Source materials, DNA and RNA purification....4
1.2 Library preparation....4
1.3 Sequencing....5
2. Genome characteristics, quality control, expression analyses....6
2.1 Genome size....6
2.1.a Flow cytometry estimation....6
2.1.b k-mer estimation....8
2.2 Lateral gene transfer events and bacterial contamination....13
2.3 Repeat content....19
2.4 Comparative transcriptomic assessments of hemipteroid reproductive biology 23
3. Automated gene annotation using a Maker 2.0 pipeline tuned for arthropods....26
4. Community curation and generating the official gene set....28

5. Curation and comparative analysis of specific gene families 33
5.1 Developmental regulation: transcription factors and signaling pathways 33
5.1.a Anterior-posterior body axis: terminal patterning system and segmentation 34
5.1.b Hox and other homeobox transcription factors 36
5.1.c Iroquois Complex (Iro-C) cluster 39
5.1.d T-box transcription factors and heart determinants 42
5.1.e Nuclear receptors 44
5.1.f Dorsal-ventral body axis: BMP/TGF-β pathway 46
5.1.g Dorsal-ventral body axis: Toll/ NFκB pathway 48
5.1.h Innate immunity 51
5.1.i Notch, Hedgehog, and Torso RTK pathways 53
5.1.j Wnt pathway 55
5.1.k Appendage patterning 59
5.1.l Germline genes 61
5.1.m Eye development 63
5.2 Structural and differentiation genes 65
5.2.a Bristle and neural development 65
5.2.b Molting and metamorphosis genes 72
5.2.c Structural cuticular proteins and pigmentation 74
5.3 Environmental adaptations 77
5.3.a Stress response 77
5.3.b Cytochrome P450s 79
5.3.c Insecticide resistance 80

5.3.d Neuropeptides and their receptors..81

5.3.e Visual genes and light detection..82

5.3.f Chemoreceptors 85

5.4 Molecular machinery 95

5.4.a Gene silencing machinery (RNAi, miRNA, piRNA)...........................95

5.4.b Sex determination and dosage compensation 97

5.4.c Epigenetic machinery..99

5.4.d Repressive C2H2 zinc finger effectors (KAP-1/ TRIM proteins) 107

6. Post-OGS v1.1 pipeline analyses 109

6.1 Protein gene orthology assessments via OrthoDB and BUSCO.......................109

6.2 Transcription factor classifications and orthology assignments 112

6.3 Gene structure evolution 113

6.4 Interspecific comparisons of metabolic enzymes 116

7. References..117

1. Genome and transcriptome sequencing and assembly

Contributors: Stephen Richards, Daniel S.T. Hughes, Shwetha C. Murali, Jiaxin Qu, Shannon Dugan, Sandra L. Lee, Hsu Chao, Huyen Dinh, Yi Han, HarshaVardhan Doddapaneni, Kim C. Worley, Donna M. Muzny, Richard A. Gibbs, Kristen A. Panfilio, Stefan Koelzer

The milkweed bug *Oncopeltus fasciatus* is one of thirty arthropod species sequenced as a part of a pilot project for the i5K arthropod genomes project at Baylor College of Medicine Human Genome Sequencing Center. For all of these species, an enhanced Illumina-ALLPATHS-LG (v. 35218) sequencing and assembly strategy enabled multiple species to be approached in parallel at reduced costs. For most species in the pilot, including *O. fasciatus*, we sequenced four libraries of nominal insert sizes 180 bp, 500 bp, 3 kb and 8 kb. The amount of sequence generated from each of these libraries is noted in Table S 1.1, with NCBI SRA accessions.

1.1 Source materials, DNA and RNA purification

Genomic DNA was extracted from an individual adult male to construct the main sequencing libraries: 180-bp, 500-bp paired end and 3-kb mate pair libraries. A fourth, larger mate pair library with 8-10 kb inserts was constructed with DNA extracted from an individual adult female, due to the higher amount of starting DNA required for this library. Additionally, to aid in genome assembly and gene prediction, RNA was extracted from three samples representing three different life history samples: an individual adult male, an individual adult female, and pooled, mixed-instar nymphs.

1.2 Library preparation

To prepare the 180-bp and 500-bp libraries, we used a gel-cut paired end library protocol. Briefly, 1 µg of the DNA was sheared using a Covaris S-2 system (Covaris, Inc. Woburn, MA) using the 180-bp or 500-bp program. Sheared DNA fragments were purified with Agencourt AMPure XP beads, end-repaired, dA-tailed, and ligated to Illumina universal adapters. After adapter ligation, DNA fragments were further size selected by agarose gel and PCR amplified for 6 to 8 cycles using Illumina P1

and Index primer pair and Phusion® High-Fidelity PCR Master Mix (New England Biolabs). The final library was purified using Agencourt AMPure XP beads and quality assessed by Agilent Bioanalyzer 2100 (DNA 7500 kit) determining library quantity and fragment size distribution before sequencing.

The long mate pair libraries with 3-kb or 8-kb insert sizes were constructed according to the manufacturer's protocol (Mate Pair Library v2 Sample Preparation Guide art # 15001464 Rev. A PILOT RELEASE). Briefly, 5 µg (for 2 and 3-kb gap size library) or 10 µg (8-10 kb gap size library) of genomic DNA was sheared to desired size fragments by Hydroshear (Digilab, Marlborough, MA), then end repaired and biotinylated. Fragment sizes between 3-3.7 kb (3 kb) or 8-10 kb (8 kb) were purified from 1% low melting agarose gel and then circularized by blunt-end ligation. These size selected circular DNA fragments were then sheared to 400 bp (Covaris S-2), purified using Dynabeads M-280 Streptavidin Magnetic Beads, end-repaired, dA-tailed, and ligated to Illumina PE sequencing adapters. DNA fragments with adapter molecules on both ends were amplified for 12 to 15 cycles with Illumina P1 and Index primers. Amplified DNA fragments were purified with Agencourt AMPure XP beads. Quantification and size distribution of the final library was determined before sequencing as described above.

1.3 Sequencing

Sequencing was performed on Illumina HiSeq2000s generating 100-bp paired end reads. Reads were pre-processed using cutadapt for adapter removal and sickle-trim for quality trimming (- min length 20bp). Subsequently, reads were assembled using ALLPATHS-LG (v35218) [1] on a large memory computer with 1 TB of RAM and further scaffolded and gap-filled using in-house tools Atlas-Link (v.1.0) and Atlas gap-fill (v.2.2) (https://www.hgsc.bcm.edu/software/). This yielded an assembly of 1.099 Gb (774 Mb without gaps within scaffolds), with a contig N50 of 4.0 kb and scaffold N50 of 340 kb, which has been deposited in GenBank (assembly accession GCA_000696205.1).

Table S 1.1: Sequencing, assembly, annotation statistics and accession numbers in Excel Supplement

2. Genome characteristics, quality control, expression analyses

2.1 Genome size

2.1.a Flow cytometry estimation

Contributors: Patricia J. Moore and J. Spencer Johnston

Genome size estimations for the *Oncopeltus* genome were obtained by flow cytometry. Four to five females and males each from the Carolina Biological Supply lab strain and a wild strain (collected from Athens, Georgia, USA; GPS coordinates: 33° 56' 52.8216" N, 83° 22' 38.3484'' W) were measured. The samples were prepared after Hare and Johnston [2]. In short, the head of a single individual was placed into 1ml of cold Galbraith buffer in a 2-ml Kontes Dounce, along with the head of a *Drosophila virilis* female (1C = 328 Mb) and a *Callosobruchus maculatus* male (1C = 1175.6 Mb) that were added as co-prepared internal standards. Nuclei from the sample and standards were isolated with 15 strokes of the loose (B) pestle, then filtered through a 40µm filter, and stained with propidium iodide at 25 mg/ml, rather than 50 mg/ml. Following at least 30 minutes of staining in the cold and dark, the amount of fluorescence of the nuclei from the sample and standard was scored with a Partec CyFlo Flow cytometer, with excitation at 532 nm provided by a Cobalt Samba laser (Solina Sweden) and PI fluorescence detected after passing a 590nm long pass filter. The 1C amount of DNA was determined as the ratio of the mean 2C peak channel number of the sample peak divided by the 2C mean sample channel number of each standard times the amount of DNA in the *C. maculatus* standard (Figure S 2.1). At least 2000 nuclei were scored under each peak. The CV was 3.0 or less for all peaks. The *D. virilis* standard was used to verify the estimated genome size of the *C. maculatus* standard. Both standards gave the genome size estimates shown, but the standard error (shown) was lower using estimates based on the *C. maculatus* standard.

The genome size of the lab and wild strains are not significantly different. However, the male genome is very slightly larger than the female (Table S 2.1). The larger genome size estimate for the male is consistent with a large neo X/Y in the male. Oncopeltus has 2n = 16 chromosomes and an XX/XY type of sex determination; the Y is largely heterochromatic and transcriptionally inactive, pairing only briefly with the X in meiosis [3].

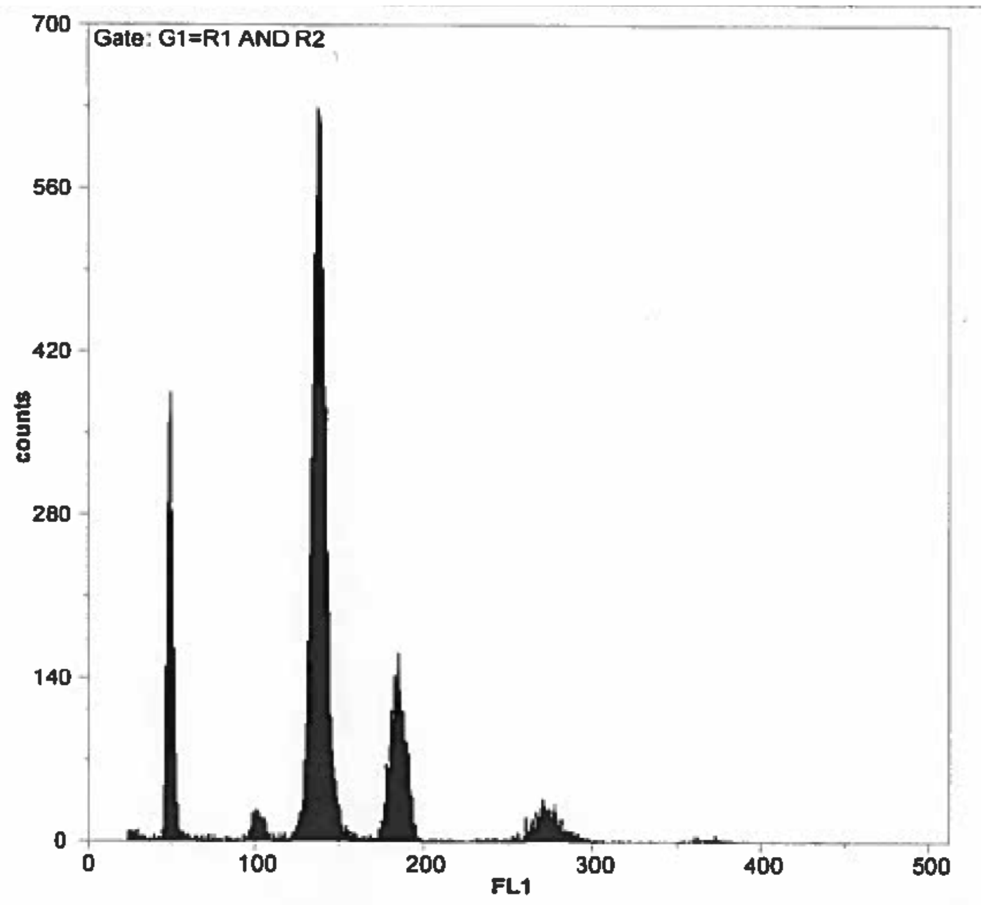

Relative Red (PI) Fluorescence

Figure S 2.1: Peaks from Flow cytometry measurement in *Oncopeltus fasciatus*. The first peak is the *D. virilis* 2C. The small second peak is the *D. virilis* 4C, the large third peak is the 2C of *Oncopeltus*, the 4th peak is the *C. maculatus* 2C peak, and the two smaller right-most peaks are the 4C of *Oncopeltus* and the 4C of *C. maculatus*, respectively.

Table S 2.1: Flow cytometry estimations of genome size. Four or five individuals were scored for each strain. The 1C (haploid) mean genome size (in Mb) is given for each strain along with the standard error based on the 4 or 5 individual estimates.

**Sample/strain**	**Individuals**	**Flow cytometry estimation**
O. fasciatus Lab F	5	922.8 +/- 4.8 Mb
O. fasciatus Lab M	4	927.9+/- 3.7 Mb
O. fasciatus Wild F	5	923.8+/- 5.9 Mb
O. fasciatus Wild M	4	928.8+/- 4.5 Mb

2.1.b k-mer estimation

Contributors: Iris M. Vargas Jentzsch and Kristen A. Panfilio

With the aim of estimating genome size, heterozygosity, and repeat content from unassembled sequencing reads, we tried several approaches to characterize the *k*-mer frequency spectrum in the *Oncopeltus* genomic dataset. As starting point we had four 100-bp Illumina read libraries: two mate-pair and two paired-end (see Table S 2.2). All libraries were filtered for quality, adapters were removed, and only correctly paired reads were retained (with the program Trimmomatic v0.30, parameters: `trimmomatic-0.30.jar PE -phred33 ILLUMINACLIP:Trimmomatic-0.30/adapters/TruSeq3-PE.fa:2:30:15:8:true LEADING:3 TRAILING:20 SLIDINGWINDOW:4:15 MINLEN:36`).

Table S 2.2: i5k Illumina library sizes after filtering.

Library	Number of reads	Expected depth of coverage
Genomic_180bp	397,866,178	36x
Genomic_500bp	148,709,592	13x
Genomic_3Kb	333,805,426	30x
Genomic_8-10Kb	189,085,040	17x

We used the programs Jellyfish2.1.4 [4] and bbmap [5], to perform *k*-mer counts on each sequencing library separately, for a range of *k* between 15 and 35. The program bbmap was initially used only to confirm the Jellyfish results, but proved more efficient by generating the same results, and even extending these over a higher range of *k*-mer depths (up to 100,000 with bbmap vs. up to 10,000 with Jellyfish). Counts for *k* > 30 could only be completed with bbmap.

K-mer frequency spectra in *Oncopeltus*

To generate the *k*-mer frequency spectrum, the frequency of occurrence of *k*-mers in the dataset (also called depth or multiplicity) is plotted against the observed counts for each of the frequencies. (Note that there is no consensus in the naming of axes for the

k-mer frequency plots, and because we are actually plotting the frequency of a frequency, either axis can be labeled as ‘frequency’.) In an ideal dataset, with no sequencing errors and where all parts of the genome are represented equally, we expect a curve with one or more clear peaks, with the highest peak corresponding to the homozygous non-repetitive fraction of the genome. This peak would ideally be centered at the expected depth of coverage of the respective dataset (or more precisely at expected coverage - 1/read length*k^{-1}), because unique regions of the genome should be sampled on average as many times as the sequencing coverage [see 6, 7, 8]. In practice, *k*-mers from repetitive regions of the genome and *k*-mers containing sequencing errors can shift or change the shape of this curve.

The *k*-mer spectra obtained for our datasets did not resemble theoretical expectations [like in 9] in that they had very shallow or non-existent peaks. Excluding *k*-mers with counts <5, which represent mostly erroneous *k*-mers [10], allowed the visualization of small peaks for all but the 500-bp dataset. All *k*-mer spectra distributions were unimodal, with a long, slowly decreasing tail towards higher *k*-mer frequencies. Among the four datasets, the most prominent peaks were observed in the mate-pair libraries (Figure S 2.2). The 500-bp dataset showed an almost monotonic exponential decay for all measured *k*-mer values, probably due to its low coverage (13%). However, the shape of the 500-bp dataset did not improve when combined with counts from the 180-bp dataset.

For each dataset, the position of the peak (frequency or depth at which the peak is centered) varied depending on the size of the *k*-mer counted: in all cases increasing *k*-mer lengths produced a shift of the peak towards lower *k*-mer frequencies (Figure S 2.3 shows the progression for the 180-bp library). This kind of shift can be due to poor data quality or bias in sequencing coverage [9, 10]. In the case of sequencing errors, these become magnified by a factor of *k* because there will be *k* erroneous *k*-mers per error, and erroneous *k*-mers are expected to have very low frequencies [11]. This magnification will shift total *k*-mer counts towards very low frequencies and reduce the difference between this noisy region and the remaining curve. The second factor, sequencing coverage bias, is typical in amplification-based sequencing approaches due to variation in amplification efficiency across the genome. This affects the probabilities of sampling *k*-mers in the same genomic frequency class,

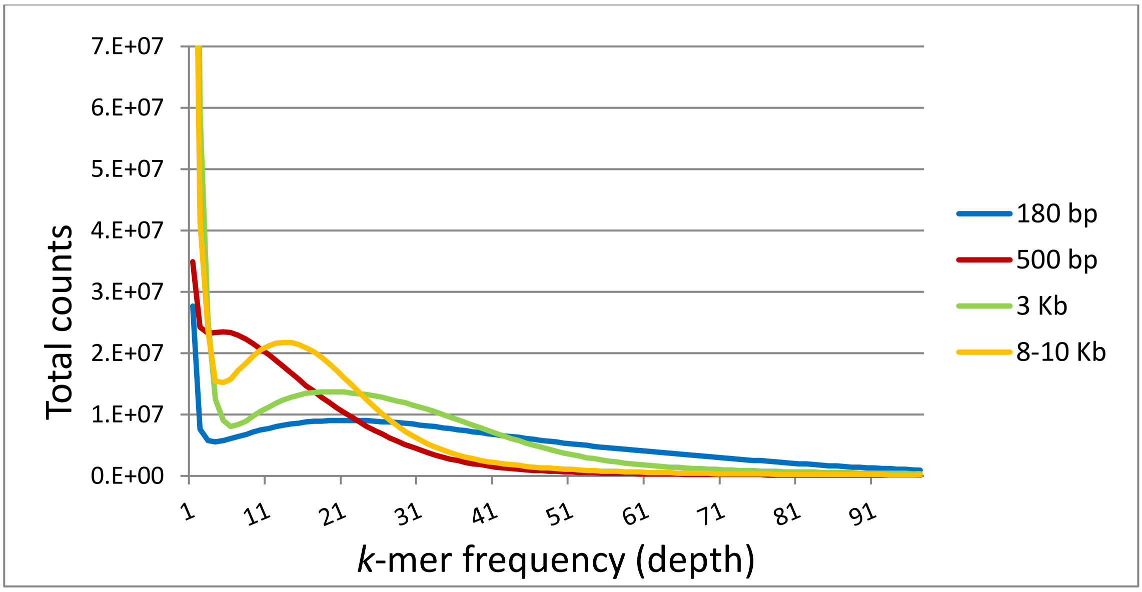

Figure S 2.2: Overlap of 17-mer spectra for the four Illumina libraries. The 8-10Kb mate pair library had the peak with the highest volume, but centered at a depth of 14, followed by the 3Kb peak at a depth of 20 and the 180bp peak centered at a depth of 23. The 500 bp library has a very shallow peak. All the peak depths were lower than the expected depth of coverage for the respective libraries (see Table S 2.2).

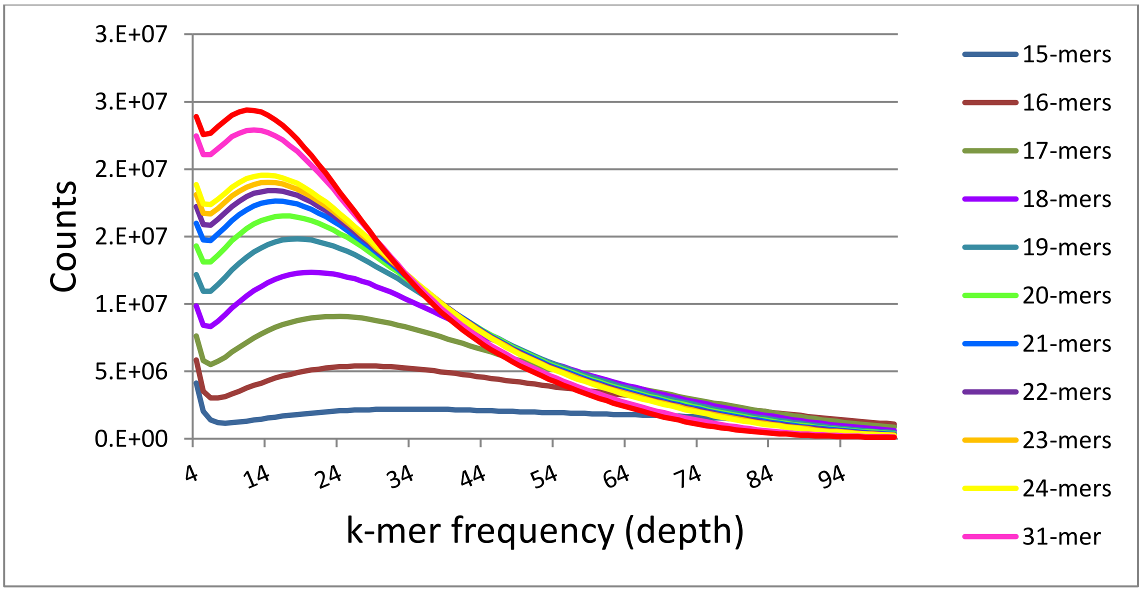

Figure S 2.3: *k*-mer spectra for the 180 bp library and variable values of k. The display is limited to depth values from 4 to 100, to better visualize the 'peaks'. For increasing *k*-mer sizes, the peak becomes more pronounced and shifted towards lower *k*-mer frequencies, whereby the inflexion in the curve separating erroneous *k*-mers from the rest of the distribution occurs at higher counts. This means that, the larger the *k*-mer size, the less distinction there is between correct and erroneous *k*-mers.

flattening the *k*-mer frequency curve and making it more difficult to observe a peak [10]. Our datasets had very good quality as inferred from Illumina quality scores, and erroneous *k*-mers are usually dealt with by excluding low frequency *k*-mers from the calculations. On the other hand, coverage bias is more difficult to deal with and requires complex modeling [see 10], which we did not do here. Unimodal distributions with peaks shifted towards lower depths were suggested to be characteristic for most animals except mammals and birds [12, 13], but these previous analyses were limited to *Drosophila* and *Apis mellifera* for the insects.

The presence of only a single peak in all our *k*-mer spectra impeded the estimation of heterozygosity. Expectations from ideal simulated data for a diploid genome show that the *k*-mers corresponding to the heterozygous part of the genome would form a second peak at about half the expected depth of coverage of the given dataset. The ratio between these two peaks depends on the heterozygosity of the genome, therefore allowing its estimation [see 10]. For *Oncopeltus* we also expected a bimodal distribution because the individual sequenced was not inbred. However, several other arthropods sequenced as part of the i5K pilot project showed two distinct peaks in their *k*-mer spectra (S.C. Murali and S. Richards, manuscript in preparation), suggesting that the relative flatness of our *k*-mer spectra may indeed be due to specific characteristics of the *Oncopeltus* genome.

Genome size estimation

We estimated genome size from all obtained *k*-mer spectra using two formulas. One is based simply on *k*-mer numbers: total *k*-mer number/coverage at the peak (formula 1) [14], while the second uses nucleotide counts from the estimate-genome-size.pl script by J. Ryan (formula 2) (https://github.com/josephryan/estimate_genome_size.pl):

$$G = T / [(M * L)/(L - k + 1)]$$

T = total nucleotides

M = depth of coverage at the peak

K = *k*-mer size

L = mean read length

For all estimations we excluded the low depth *k*-mers forming the left side sharp peak in the *k*-mer spectra curves; the threshold was set at the lowest turning point of each curve, which varied between depth values of 4 and 6. The estimates from our calculations (Table S 2.3) were in most cases too high compared to the genome size estimated by flow cytometry of 920 to 930 Mb (see Section 2.1.a). The best approximation was obtained from the 15-mer spectra, because this curve had the peak at the highest depth of coverage. This depth of coverage (or *k*-mer frequency) at the peak was the main factor affecting the estimations; it became smaller for larger values of *k*, resulting in an overestimation of genome size. As mentioned above, it is probably due to sequencing coverage bias that all our sequencing libraries had the peak of the *k*-mer spectra centered at much lower depth of coverage values than the library sequencing depth. Consequently, about one third of the genomic *k*-mers were indistinguishable from erroneous low frequency *k*-mers, precluding more accurate estimations from the *k*-mer frequency spectra.

Table S 2.3: Estimates of genome size for various *k*-mer sizes from the 180-bp library.

k	Number of erroneous *k-mers* (Depth ≤5)	Total *k*-mers	Peak	Genome size estimate from formula 1 [Mb]	Genome size estimate from formula 2 [Mb]
15	96,179,874	31,916,279,278	32	994	1,024
16	190,554,545	28,967,316,992	26	1,107	1,261
17	282,378,853	28,797,962,402	23	1,240	1,425
18	360,059,200	28,595,269,396	19	1,486	1,725
19	423,188,873	28,496,383,832	17	1,651	1,928
20	473,437,832	28,138,862,469	15	1,844	2,185
21	512,152,262	27,895,835,094	15	1,826	2,185
22	541,893,371	27,644,584,878	14	1,936	2,342
23	565,679,336	27,385,616,052	13	2,063	2,522
24	586,102,741	27,119,171,927	13	2,041	2,522

2.2 Lateral gene transfer events and bacterial contamination

Contributors: Rose Richter, Amanda Dolan, Kristen A. Panfilio, Stefan Koelzer, John H. Werren

LGT candidates

For *Oncopeltus fasciatus*, 20 lateral gene transfer (LGT) candidates from bacteria were predicted using computational methods and assessed by subsequent manual annotation. These have a blastn similarity score to prokaryotes <1e-10 or a bitscore >75. We then assessed these LGT candidates, by several criteria, including information on the possible prokaryotic source of the LGT, gene annotation, and expression support (Table S 2.4). Of these, 19 have at least one flanking gene on the scaffold, and five were empirically validated by PCR amplification from *Oncopeltus* gDNA (see Methods, below). An additional 61 potential LGTs were deemed weak candidates, based on the presence of eukaryotic similarity or lack of sufficiently strong bacterial similarity, and are not addressed in this report.

Table S 2.4: Information on candidate LGTs in *O. fasciatus* (in Excel supplement file).

Noteworthy among the LGTs are two genes that are involved in peptidoglycan biosynthesis in bacteria. Peptidoglycan (also known as murein) is an important constituent of bacterial cell walls, particularly in gram-positive bacteria. The LGT genes *alanine racemase* and *UDP-N-acetylenolpyruvoylglucosamine reductase* (*murB*) appear to have been transferred from *Vitreoscilla* and *Chlamydia* bacteria, respectively, have expression support, and were empirically validated in *Oncopeltus* (Table S 2.4). Alanine racemase is an enzyme that catalyzes a structural conversion from L-alanine to D-alanine, with D-alanine being used in murein biosynthesis in bacteria. Although alanine racemase has been found in some marine invertebrates [15], the LGT found in *Oncopeltus* is phylogenetically embedded among *alanine racemase* genes from bacteria (based both on nucleotide and protein analyses: Figure S 2.4) and was not detected in any other invertebrates based on blastn and blastp searches. Similarly, murB shows a strong signature of lateral gene transfer from *Chlamydia* (Figure S 2.5). The potential functions of these genes in *Oncopeltus* are

unknown, but worthy of further investigation. Possibilities may include defense against bacteria or enhanced ability to digest bacteria for nutrition.

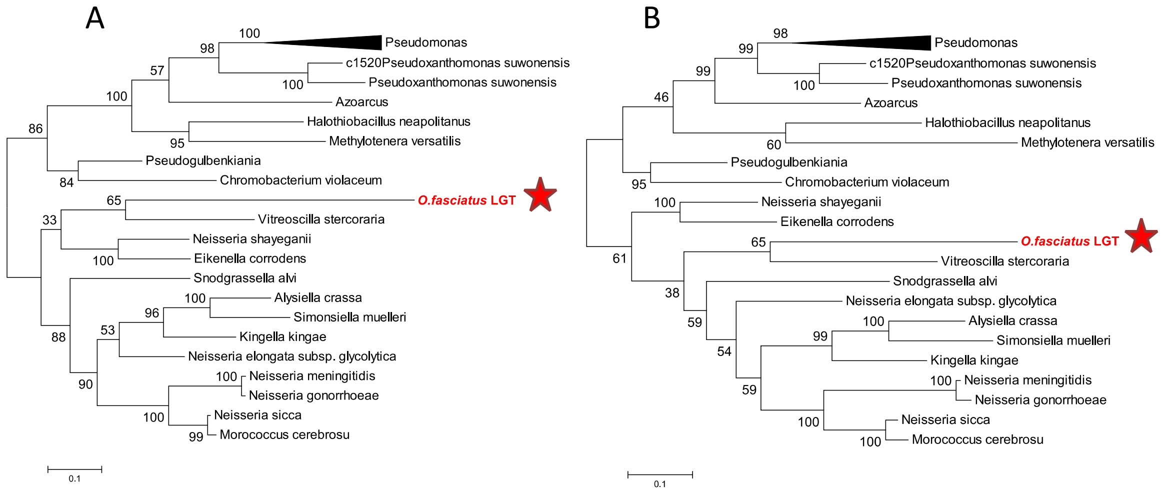

Figure S 2.4: Maximum likelihood phylogenies of the alanine racemase LGT using **(A)** amino acid and **(B)** nucleotide sequences of a 1053-bp region, showing the *Oncopeltus* sequence (red stars) embedded among bacterial orthologs. Sequences with strong similarity to the racemase LGT in NCBI's nr/nt database were aligned in MEGA 5.1 using ClustalW. Sequences were trimmed to 1053 bp, and the alignment was manually curated for accuracy. Sequences included top hits and strong matches to the *Oncopeltus* sequence. The Jones-Taylor-Thornton model was used, with 500 bootstrap replications to create maximum likelihood trees.

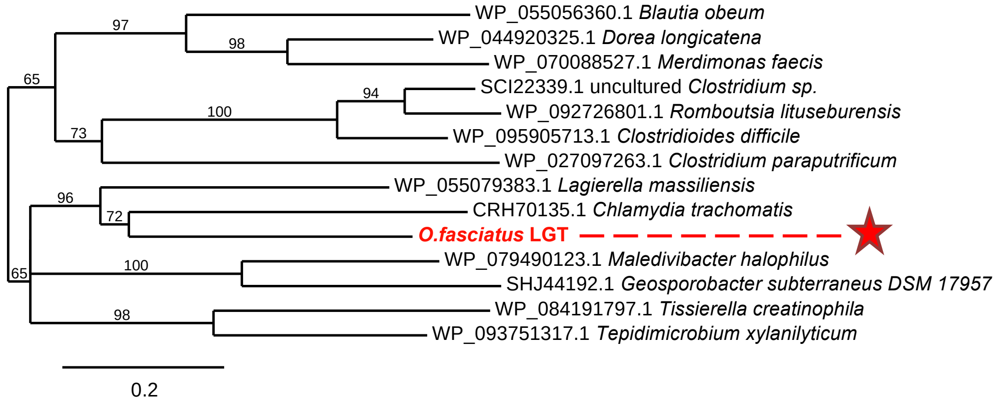

Figure S 2.5: Maximum likelihood phylogeny of the UDP-N-acetylenolpyruvoylglucosamine reductase LGT, using amino acid sequences of the top blastp hits in NCBI's nr database, without taxonomic restriction, showing the *Oncopeltus* sequence (red star, 307 aa ORF) embedded among bacterial orthologs.

Whereas the two LGTs with potential roles in cell wall synthesis appear to be unique to *Oncopeltus* among the insects, an older LGT event led to the introduction of the cell wall degradation enzyme endo-1,4-beta-mannosidase [16] in the common ancestor of *Oncopeltus* and the stink bug *Halyomorpha halys*, a fellow member of the hemipteran infraorder Pentatomomorpha (Figure S 2.6, and see main text).

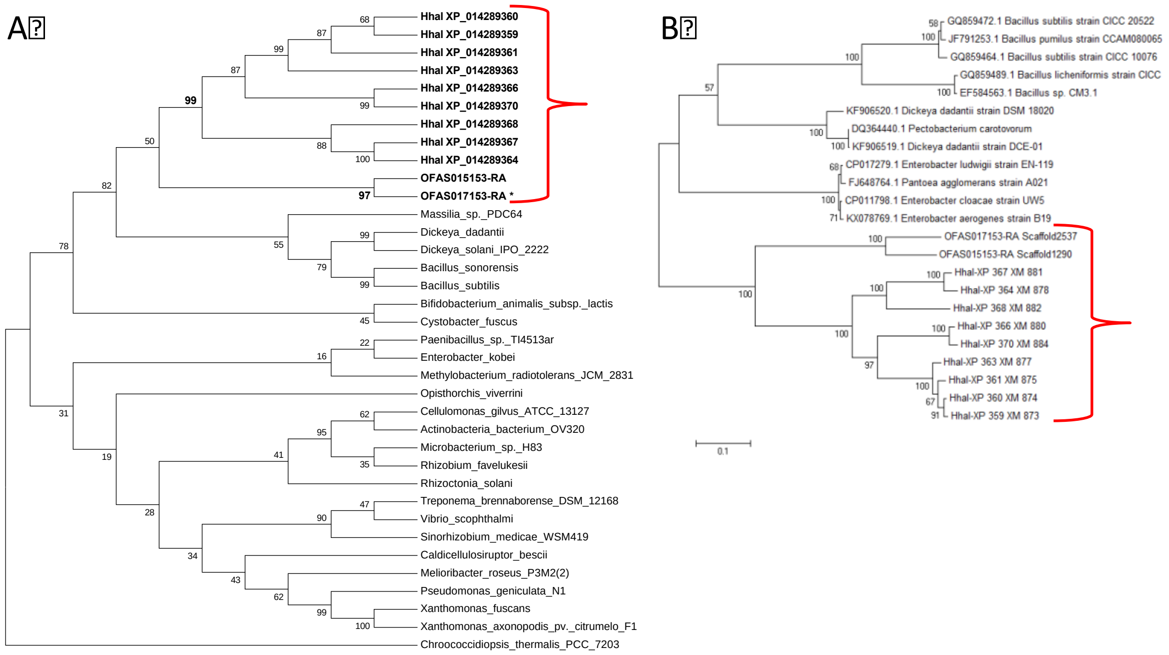

Figure S 2.6: Maximum likelihood phylogenetic analyses of the endo-mannosidase LGT using **(A)** amino acid and **(B)** nucleotide sequences of a 1037-bp region, with the *Oncopeltus* and *Halyomorpha* sequences forming a well-supported clade with species-specific expansions (red brackets) among bacterial orthologs. The protein data **(A)** are shown in a bootstrap consensus tree. See main text for further details.

Finally, although not detected by our DNA-based LGT pipeline, we also found in *Oncopeltus* the ancient lysozyme glycoside hydrolase (GH25) LGT of *Wolbachia* origin, which is present in other hemipterans [17]. GH25 (also known as muramidase) is implicated in antimicrobial defense, and encodes an enzyme that also breaks down the bacterial cell wall. Therefore, we have detected various LGTs in *Oncopeltus* that are implicated in bacterial or plant cell-wall metabolism, and range in age from relatively recent (*Oncopeltus* specific), to presence in a subset of Hemiptera, to an LGT of apparently ancient origin in the Hemiptera.

Potential bacterial scaffolds

In addition to LGT candidates, there were five candidate "contaminating" bacterial scaffolds identified. Typically we find scaffolds that are probably from bacteria and these most likely are from bacterial associates of the insect. Thus, they can be informative. The identified scaffolds have homology to *Sphingomonas sanxanigenens* (Scaffold 13019), *Acinetobacter sp.* (Scaffold 15841), *Methylobacterium oryzae* (Scaffold 16784), *Paracoccus marcusii* (Scaffold 17186), and *Comamonas testosterone* (Scaffold 15798). The contaminating scaffolds are small (919 to 3841 bp), with stretches of only 0.9 to 1.4 Kb each with strong blast similarity to the bacterial sequence. Furthermore, these scaffolds do not represent entire bacterial genomes, and each has highest homology to a different bacterial species. A caveat is that we cannot always rule out that these "bacterial" scaffolds are actually large LGTs. Future comparison of the depth of coverage on these scaffolds to depth of coverage in genomic scaffolds (single copy regions, not TEs) could clarify this, as bacterial contaminants are typically at different density from nuclear genes (sometimes much higher, sometimes much lower). Note that the overall low number of such "contaminating" scaffolds is consistent with the method of template preparation for sequencing, as DNA was prepared from dissected adults from which gut material was removed. This also further strengthens the likely validity of the strong LGT predictions.

Methods

Two different computational pipeline scripts were used to identify LGT candidates and contaminating bacterial scaffolds in the genome assembly.

Information on the "old" computational pipeline

The scaffold assemblies (Genome File Name: Ofas.contaminationfree.scaffolds, downloaded from the i5k FTP site on 6-10-15) were run through this pipeline [18], which compares sequences for matches to ~1000 different bacterial genomes and compares the blast matches to a set of up to 9 reference eukaryotic genomes. For *O. fasciatus*, the animal database contained transcripts from the following animal genera: *Anopheles, Drosophila, Xenopus, Tribolium, Daphnia, Mus,* and *Homo sapiens.*

Scaffold outputs were then sorted into potential LGTs, contaminating bacterial scaffolds or likely conserved scaffolds. Sorting was based on length of the scaffold, length of bacterial matches across the scaffold, and difference score in bacterial blast match relative to eukaryotic match [18]. This pipeline focuses on the best blastn bacterial hit (highest e-value) on each scaffold, and therefore additional regions with bacterial similarity were found manually using NCBI blastn.

Information on the "new" computational pipeline

Since the old pipeline sorted scaffolds based on only the top prokaryotic hit information, there was concern that LGT candidates were being missed if they were not the best hit. The "new" computational pipeline breaks long scaffolds into 1000-bp intervals and searches each of them against the bacterial database. Any positive hits of the 1000-bp regions were then searched against the animal database. The bacterial database contained about 1000 bacterial species and was masked for low complexity regions using the NCBI Dustmasker function. The animal database for *O. fasciatus* contained transcripts from a representative from each of the following animal genera: *Anopheles, Drosophila, Xenopus, Tribolium, Daphnia, Strongylocentrotus, Mus, Homo sapiens, Aplysia, Caenorhabditis, Hydra, Monosiga,* and *Acanthamoeba.* The significance e-value cut-off used was $<1e-5$ for both the animal and bacterial hits. Regions of bacterial similarity that fell from the end of one 1000-bp interval to the adjacent interval were joined. Only the putative LGT regions ≥ 100 bp and without any hits to the animal database were used in the final analysis.

Manual annotation

The candidate LGT outputs were then manually curated by the following basic steps. Potential LGT regions on the scaffolds were searched via blastn (NCBI) in the nr/nt database. If this indicated that the region is simply a conserved gene in insects and other metazoan organisms, it was noted as conserved and disregarded. If not, the region was searched via blastx to the nr/nt database to determine if it is a conserved gene or remains an LGT candidate. For LGT candidates, flanking genes were analyzed by both blastn/blastx of the flanking sequence and/or by observations of gene models and transcription models on the species-specific Apollo web browser

(https://apollo.nal.usda.gov/oncfas/selectTrack.jsp). Outputs meeting the criteria of a potential LGT were labeled as an LGT candidate in the annotation comments.

After the OGS had been generated, further manual assessment was performed, including inspection of transcriptome and RNA-seq read evidence tracks in the genome browser, and by cross-referencing to 4 stage-specific libraries (embryo/maternal, nymph, adult female, adult male) if the LGT could be assigned to a given OGS gene model. Additional GenBank NCBI blastn and blastp analyses were also performed in light of finalized OGS gene models from community curation (Section 4, above).

Experimental validation of selected LGT candidates

PCR amplification from *Oncopeltus* genomic DNA (gDNA) template was used to validate five selected LGT candidates, with three biological replicates for template gDNA template. Amplification followed a published thermocycle program for LGT candidates [19] with the following modifications: annealing temperature of 54.4 ºC and a 2.5-minute extension step. Primers were designed against the genome assembly and chosen to flank the LGT region (sequences listed in Table S 2.4). The resulting amplicons (1.1-1.7 kb) were cloned, and two clones per LGT amplicon were Sanger sequenced and confirmed to fully encompass the predicted LGT sequence.

2.3 Repeat content

Contributors: Iris M. Vargas Jentzsch and Kristen A. Panfilio

The repeat content of the assembly was assessed with RepeatModeler [20] using default parameters, based on a species-specific repeat library generated *de novo* with RECON [21], RepeatScout [22], and Tandem Repeats Finder [23]. For comparative analysis, the same analysis was performed on the genome assemblies of two fellow hemipterans: the pea aphid *Acyrthosiphon pisum* (version 2: http://bipaa.genouest.org/data/public/a_pisum/assembly2_scaffolds.fasta.bz2) and the bed bug *Cimex lectularius* (ftp://ftp.hgsc.bcm.edu/I5K-pilot/Bed_bug/genome_assemblies/). As a result, only 25% of the *Oncopeltus* assembly could be identified as repetitive, while in 35% of the *Cimex* assembly and 31% of the pea aphid assembly were covered by repeats (see main text and Table S 2.5 for discussion and additional information). Comparisons between genome assemblies need to be done with caution, as differences in sequencing technologies and assembly strategies can produce spurious results. The pea aphid assembly is based on Sanger sequencing, while the bed bug and *Oncopeltus* assemblies were produced from Illumina sequence by the i5K project. Nevertheless, the bed bug assembly is much smaller than the *Oncopeltus* one (650 Mb compared to 1,099 Mb), and its assembly quality is much higher (contig N50 of 4,047 bp for *Oncopeltus* and 23,511 bp for *Cimex*). With 17,222 scaffolds and 325 million undetermined nucleotides, the current *Oncopeltus* assembly is considerably fragmented. One of the main causes of fragmentation in genome assemblies is a high proportion of repeats in the assembly [24]. Thus, the analysis of repetitive content in a highly fragmented assembly is essentially flawed.

In an attempt to improve the *Oncopeltus* assembly, we generated additional sequencing reads with the single molecule real time sequencing technique from Pacific Biosciences. This technology can produce very long reads with the potential to span gaps and repetitive sequences, overcoming biases associated with features like GC content and repeats [24, 25]. The sequencing was done at the Genome Centre of the Max Plank for Plant Breeding Research (MPIPZ Cologne) with a PacBio RS II machine. The single template library was generated from pooled gDNA from an adult

virgin female and mixed-stage eggs. A total of 34 SMRT cells were sequenced generating a set of reads to an approximate coverage of 8× (Table S 2.6).

We first attempted to generate a hybrid assembly from PacBio and Illumina reads, using ALLPATHS-LG. At the time of writing this paper we had no success in generating a hybrid PacBio-Illumina assembly, due to excessive requirements of RAM by the program (>500 Gb). Nevertheless, initial ALLPATHS-LG estimates of total repetitive content placed the value at 68% with these hybrid data.

Thus, we also used the PacBio subreads to perform gap-filling on the i5k scaffolds with PBJelly Version: 13.10.22 [26] with the following blastr parameters: ‘minMatch 8 -minPctIdentity 70 -bestn 1 -nCandidates 20 -maxScore -500 -nproc 4 –noSplitSubreads’. Gap-filling substantially reduced the proportion of undetermined nucleotides (assembly gaps) from 30% to 6%, with an attendant modest reduction in the number of scaffolds (17095 scaffolds, N50 = 409 Kb), while the assembly size increased from 1,099 to 1,361 Mb.

This increase in assembly size is problematic because the initial i5K genome assembly (1,099 Mb) already had a slightly larger assembly size compared to the genome size estimation by flow cytometry (925 Mb, see Section 2.1.a). On the other hand, preliminary estimations of genome size and repeat content by ALLPATHS-LG on all raw data (PacBio and Illumina) with k=25 produced estimations of genome size and repeat content of 1.51 Mb and 58%, respectively. We would expect that measurements of genome size by flow cytometry are more reliable than those obtained with bioinformatics approaches, especially because we did not obtain satisfying results from *k*-mer analyses (see Section 2.1.b). Nevertheless, published examples from other insect species show that the *k*-mer estimations also reasonably agree with laboratory measurements, both for genomes around 600 Mb in length like in the winter moth and whitefly [see 7, 8] as well as for the very large genome of the migratory locust [27]. Therefore, there is something particularly challenging in our current *Oncopeltus* dataset, and we decided to do further analyses to check if any more information could be gained by including PacBio data into our assembly. Hereafter we will call the gap-filled Illumina assembly the ‘PBJelly assembly’.

For an initial quality assessment on the PBJelly assembly, we compared it to the Illumina assembly with respect to presence, completeness, and copy number for benchmarking universal single-copy orthologs of protein coding genes (BUSCO, v. 3,

[28, 29]) based on expectations across the Insecta. The two assemblies are highly similar for these metrics, with slight improvements for completeness at the expense of only a minor increase in duplicates in the PBJelly assembly (Table S 2.7; see Section 6.1 for the complete BUSCO analysis on the Illumina assembly). Specifically, this analysis documents a slight improvement in reducing the proportion of fragmented genes (from 3% to 1.5%) and of missing genes (0.5% reduction), with only a marginal gain in duplications (0.5% increase). Given the starting high quality of the Illumina-only assembly for protein coding gene content, and the fact that we are endeavoring to produce a better hybrid assembly with new computational approaches (unpublished work in progress), we restricted use of the gap-filled assembly in the current study to our repeats analysis, and we retain the Illumina assembly as the current definitive/official version.

Repeat analysis with RepeatModeler on the PBJelly assembly revealed an increase in all repeat categories with the exception of satellites, which are still not represented at all, implying that no large tandem repeats could be unambiguously placed with PBJelly (Figure S 2.7 A). The repeat content within this assembly amounted to 32%, which is now closer to the repeat content detected by RepeatModeler in the pea aphid (31%) and bed bug (35%) genomes (details in Table S 2.5), but is still low compared to the repeat content reported for similarly sized genomes, like the brown plant hopper (1.2 Gbp assembly with 48.6% repeats: [30]), and notably lower than our ALLPATHS-LG estimate of 58% (Figure S 2.7 B). The absolute coverage, however, is almost double that in the *Cimex* assembly and more than 2.5 times that in the pea aphid assembly (see main text Fig. 5b). The majority of the repeats found by RepeatModeler were in the “unknown” category, probably because they were too short or incomplete to be identified unambiguously. Lastly, although our approach to identify repeats in *Oncopeltus* was a combination of *de novo* and homology-based prediction approaches, sensitivity could be enhanced by further manual curation of the *de novo* library [31], as was done for the pea aphid [32].

Table S 2.5: Comparison of repeat content between three bug species (in Excel Supplement).

Table S 2.6: Details on additional sequencing with PacBio.

Chemistry	PacBio RS II: P4-C2 and P6-C4
Number of SMRT cells	34 (2 SMRT cells with P6-C4)
Total number of reads	2,135,043
Average insert size	15,760 nt
Read length range	35 to 46,753 nt
Coverage	8x

Table S 2.7: BUSCO v3 statistics for gene count and percentage representation of the Insecta dataset (n= 1658) for the PBJelly assembly compared to the original i5K Illumina assembly.

**Dataset**	**Complete (single copy and duplicated)**	**Duplicated (subset of "complete")**	**Fragmented**	**Missing**
Illumina i5K assembly	1,568 (94.6%)	23 (1.4%)	50 (3.0%)	40 (2.4%)
PB Jelly assembly	1,605 (96.8%)	32 (1.9%)	22 (1.3%)	32 (1.9%)

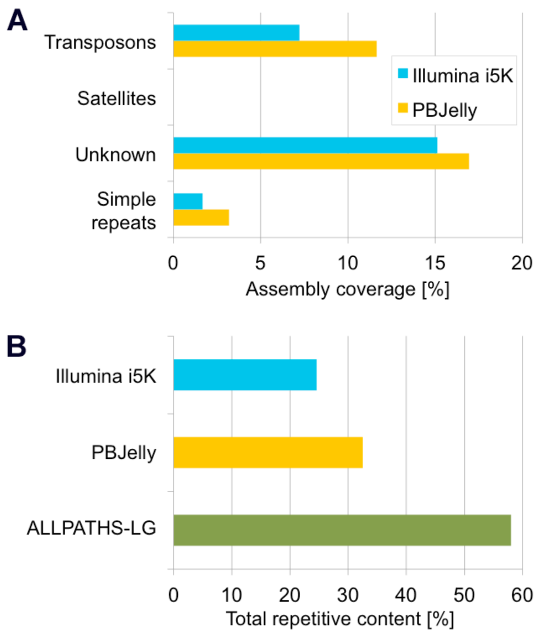

Figure S 2.7: Comparison of repeat content estimations. **(A)** Relative proportions of selected repeat categories in Oncopeltus, compared between two assembly versions. "Illumina i5K" is the current official assembly based solely on Illumina short read data. "PBJelly" is the same i5K assembly after gap filling with PacBio reads (see also Supplemental Note 2.3). Even after gap filling, it was not possible to identify large tandem repeat structures of the satellites category in these analyses with RepeatModeler. **(B)** Total repetitive content estimations for Oncopeltus based on different genome assembly versions. In addition to the assemblies used in panel (a), a third dataset, "ALLPATHS-LG", represents an estimation derived from initial attempts to generate a hybrid assembly with Illumina and PacBio reads.

2.4 Comparative transcriptomic assessments of hemipteroid reproductive biology

Contributors: Christopher J. Holmes and Joshua B. Benoit

To gain insight into stage- and sex-specific enrichment in gene expression, we compared RNA-seq datasets for *Oncopeltus* with three other hemipterans (*Cimex lectularius*, *Acyrthosiphon pisum*, *Pachypsylla venusta*) and with the thysanopteran *Frankliniella occidentalis* as a close outgroup within the hemipteroid assemblage (Tables S 2.8-2.10, Figure S 2.8).

For *Oncopeltus*, we further used our known ("i5K") RNA-seq datasets for an adult male, an adult female, and mixed-instar nymphs to assess a previously published dataset for an *Oncopeltus* adult of unspecified sex ("Andolfatto", [33]). Genes expressed in the Andolfatto set are consistent with this sample being most similar to the i5K male sample. Overall, the Andolfatto set had nearly 50% fewer differentially expressed genes in relation to the i5K male RNA-seq set when compared to either the i5K female or i5K nymph RNA-seq sets. Also, sperm-specific genes, such as serine-threonine kinases, showed a noticeably higher level of expression in the Andolfatto set even compared to the male-specific i5K set. However, vitellogenin-specific genes are also detectable in the Andolfatto set, albeit at lower levels compared to the female-specific i5K set. Thus, it appears that the Andolfatto set is mainly composed of male-specific genes, but does represent female- and nymph-specific genes as well.

Methods

Sex-specific and developmental stage-specific RNA-seq analyses were conducted according to published methods [34, 35], with modifications. In addition to the RNA-seq datasets generated in this project for *Oncopeltus* (GenBank Bioproject: PRJNA275739), RNA-seq datasets for other hemipteroid species were sequenced as parts of other i5K species projects (*Cimex lectularius*, PRJNA275741; *Acyrthosiphon pisum*, PRJNA209321; *Pachypsylla venusta*; PRJNA275248; *Frankliniella occidentalis*, PRJNA203209). These RNA-seq sets were used to examine transcriptional differences between males, females, and nymphs (if available). RNA-seq sets were evaluated with FastQC and trimmed with CLC Genomics (CLC Bio).

Reads required at least 90% similarity at 70% of the transcript length with only two mismatches. Each read was permitted to match up to five locations. Transcripts per million (TPM) was used as a proxy for transcript levels. The Baggerly's test (beta-binomial distribution statistic) followed by Bonferroni correction at 0.01 and two-fold difference between samples was used to identify genes with significant enrichment in a specific sample. This stringent statistical analysis was used because only a single replicate was available for each treatment. Genes were identified by BLASTx searches in a GenBank non-redundant protein database for arthropods (e-value ≤0.001). Following the identification of stage-specific enriched sets, these sequences were compared between life history stages in *Oncopeltus* and pairwise between *Oncopeltus* and each of the other four hemipteroid species. Overlap was visualized by Venn diagram (http://bioinformatics.psb.ugent.be/webtools/Venn/).

Table S 2.8: Information for each hemipteroid species, including paired-end read, gene, and sequence counts for individual data sets.

**Species (Dataset)**	**Sex/ Stage**	**Paired-End Reads**	**Genes**	**# Sequences, 2-Fold Increase**
Acyrthosiphon pisum	Male 1	22,908,878	36,939	2,940
	Male 2	18,692,759		
	Female 1	12,924,374		2,883
	Female 2	17,201,208		
Cimex lectularius	Male	34,043,709	14,214	1,619
	Female	26,526,400		1,657
Frankliniella occidentalis	Male	24,336,855	17,553	646
	Female	22,622,821		984
	Nymph	32,515,059		497
Oncopeltus fasciatus (published adult, "Andolfatto")	N/A	27,109,674	19,811	to known male: 1,929
				to known female: 3,906
				to known nymph: 3,421
Oncopeltus fasciatus (i5K)	Male	22,196,171	19,811	1,180
	Female	30,342,335		594
	Nymph	20,508,626		400
Pachypsylla venusta	Male	22,103,387	14,390	440
	Female	37,520,371		2,317
Rhodnius prolixus	N/A	N/A	15,078	N/A

Table S 2.9A-C: Hemipteran gene comparisons for adult males, adult females, and nymphs. Gene IDs associated with the Venn diagrams in Figure S 2.8D-F (in Excel supplement file).

Table S 2.10A-F: Hemipteran sex- and stage-specific comparisons. Gene IDs, fold-change, and significance for all sex- and stage-specific comparisons (in Excel supplement file).

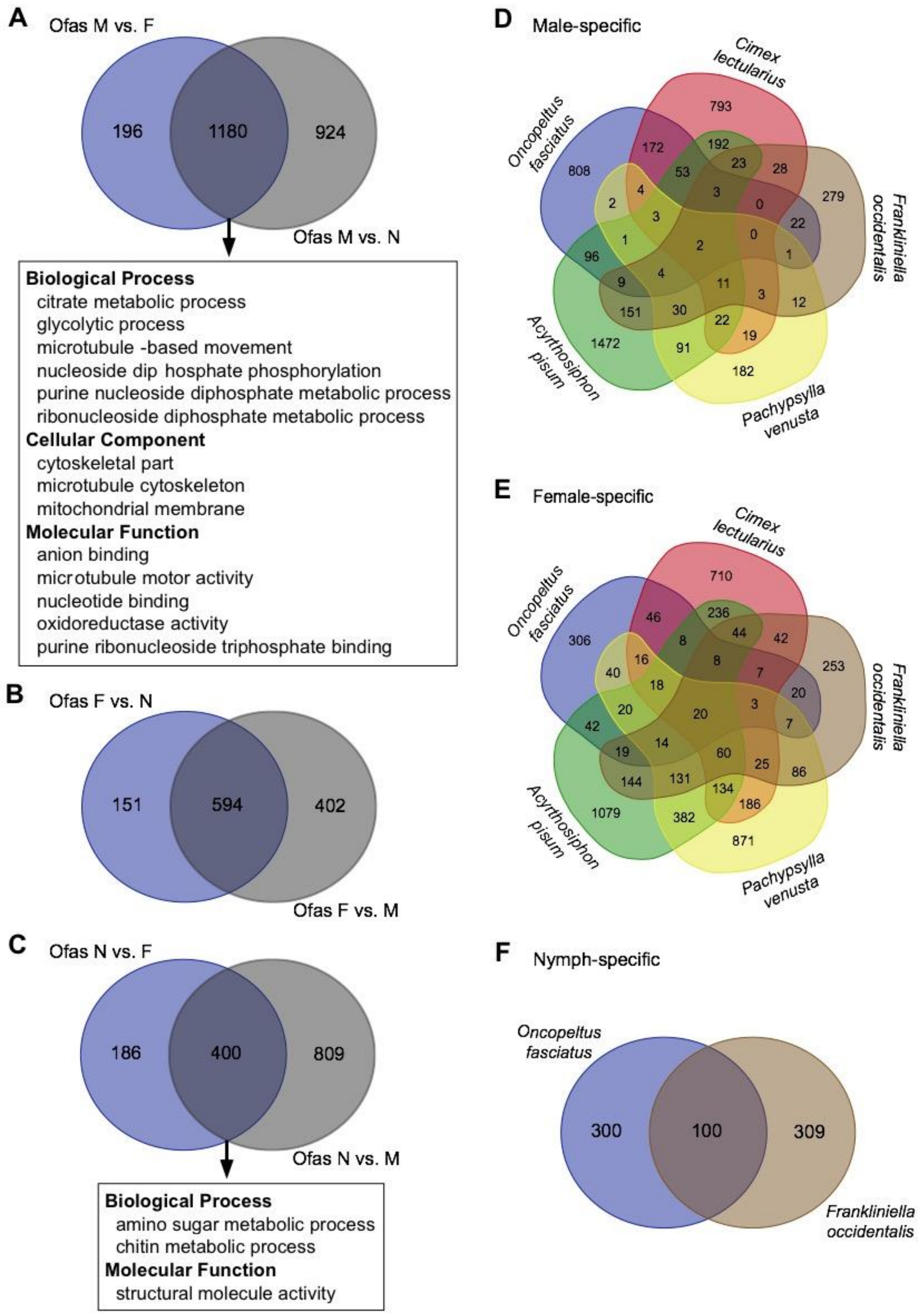

Figure S 2.8: Venn diagrams show the number of genes associated with stage-specific comparisons within *Oncopeltus* **(A-C)** and across hemipteroid species **(D-F)** for: adult males (A, D), adult females (B, E), and nymphs (C, F). Boxed terms represent GO terms that were significantly upregulated compared to the annotated genome of *Rhodnius prolixus*.

3. Automated gene annotation using a Maker 2.0 pipeline tuned for arthropods

Contributors: Dan S.T. Hughes and Stephen Richards

Oncopeltus fasciatus was among 28 i5K pilot species for which genome assemblies were subjected to automatic gene annotation using a Maker 2.0 annotation pipeline tuned specifically for arthropods. The pipeline is designed to be systematic, providing a single consistent procedure for the species in the pilot study and scalable to handle hundreds of genome assemblies, using both protein and RNA-seq evidence to guide gene models, and targeted to utilize extant information on arthropod gene sets.

The core of the pipeline was a Maker v2.28 [36] instance, modified slightly to enable efficient running on our computational resources. The genome assembly was first subjected to *de novo* repeat prediction (RepeatModeler 1.0.8 [20]) and CEGMA analysis to generate gene models for initial training of the *ab initio* gene predictors. Three rounds of training of the Augustus [37] and SNAP [38] gene predictors within Maker were used to bootstrap to a high quality training set. Input protein data included 1 million peptides from a non-redundant reduction (90% identity) of Uniprot Ecdysozoa (1.25 million peptides), supplemented with proteomes from eighteen additional species (*Strigamia maritime, Tetranychus urticae, Caenorhabditis elegans, Loa loa, Trichoplax adhaerens, Amphimedon queenslandica, Strongylocentrotus purpuratus, Nematostella vectensis, Branchiostoma floridae, Ciona intestinalis, Ciona savignyi, Homo sapiens, Mus musculus, Capitella teleta, Helobdella robusta, Crassostrea gigas, Lottia gigantean, Schistosoma mansoni*), leading to a final non-redundant evidence set of 1.03 million peptides.

RNA-seq data derived from *Oncopeltus* adult males, adult females, and mixed sex juveniles (see also Table S 1.1) were used judiciously to identify exon-intron boundaries, but with a heuristic script to identify and split erroneously joined gene models. We used CEGMA models for quality control purposes: of 1,977 CEGMA single copy ortholog gene models, 1,915 (97%) were found in the assembly and 1,837 (93%) in the final predicted gene set – a reasonable result given the small contig sizes of the assembly. Finally, the pipeline uses a nine-way homology prediction with human, *Drosophila*, and *C. elegans*, and InterPro Scan5 to allocate gene names.

The automated gene sets are available from the Baylor College of Medicine Human Genome Sequencing Center (BCM-HGSC) website:

https://www.hgsc.bcm.edu/milkweed-bug-genome-project

as well as the National Agricultural Library (NAL):

https://i5k.nal.usda.gov/Oncopeltus_fasciatus

where a web browser of the genome, annotations, and supporting annotation data is accessible. Furthermore, the genome assembly [39], Maker gene set predictions [40], and official gene set v. 1.1 ([41], see next section) are available as citable databases hosted by the NAL Ag Data Commons.

4. Community curation and generating the official gene set

Contributors: Iris M. Vargas Jentzsch, Monica F. Poelchau, Daniel S. T. Hughes, Monica C. Munoz-Torres, Christopher P. Childers, Chien-Yueh Lee, Mei-Ju Chen, Kristen A. Panfilio

The automated gene set from the MAKER pipeline, consisting of 19,615 gene models, was subjected to manual curation to assess the completeness and accuracy of gene models. This was done using the aforementioned web browser set up by the National Agricultural Library (https://i5k.nal.usda.gov/Oncopeltus_fasciatus), which allowed coordinated editing of gene models and their metadata in real time among the 16 research groups involved in the curation process. Each group curated gene families based on their own research interests and expertise, which was coordinated through a shared Google document.

The general procedure for manual curation involved retrieving homologous sequences for the genes of interest from NCBI, either from other hemipteran species (*e.g.*, *Acyrthosiphon pisum*) or from insects with high quality annotations like *Drosophila melanogaster* and *Tribolium castaneum*. These were blasted against the *Oncopeltus* assembly (generally, tblastn with a homologous protein query against the genome assembly) either in a standalone fashion or through the blast instance at the National Agricultural Library (NAL): https://i5k.nal.usda.gov/webapp/blast/. The blast results were visualized in the Apollo v.1.0.4 instance for *Oncopeltus* (https://apollo.nal.usda.gov/oncfas/selectTrack.jsp), where the corresponding automated annotation models could be inspected and edited. To confirm homology, protein translations of the edited models were blasted back into NCBI. Orthology, intron/exon boundary assessments, and protein sequence completeness were further determined by manual inspection and correction of protein alignments generated with Clustal Omega (http://www.ebi.ac.uk/Tools/msa/clustalo/).

Possible gene duplications were assessed by performing tblastn searches on the *Oncopeltus* scaffolds using the protein sequences of completed annotation models as queries, and then re-blasting the resulting hit sequences into GenBank for Arthropoda hits. Phylogenetic analysis to confirm the paralogous status of the gene models was based on trees constructed with selected protein sequences among the GenBank blast hits (*e.g.*, selection favored broad taxonomic sampling within the

Arthropoda and avoided accessions labeled as 'predicted'). Phylogenies were primarily generated at the Phylogeny.fr site (http://www.phylogeny.fr/, [42, 43]) with the default pipeline (MUSCLE v. 3.8.31 alignment, GBLOCKS v. 0.91b curation, PhyML v. 3.1/3.0 aLRT maximum likelihood tree construction), unless otherwise stated.

Special care was required for curating genes that were only partially predicted in the assembly. *Ab initio* gene prediction is difficult across big gaps (when parts of the gene are on different contigs), and a complete gene prediction is often not possible if parts of the same gene are on different scaffolds. Each part of a gene split across scaffolds may have its own automated model, or some parts may lack models altogether. Split gene models are to be expected in fragmented genome assemblies, because sequence gaps make gene prediction difficult [44]. Furthermore, having multiple good blast hits for the same gene on different scaffolds could indeed be due to the gene being split across multiple scaffolds, or to either gene duplication or conserved sequence within a gene family. If the automated model to be curated only represented part of the query sequence, the search was repeated with only the missing part of the query, in order to focus the blast search on relevant local alignment regions. If this query sequence was very short, turning off the low complexity filter in the blast options increased the probability of getting a hit. For genes split across multiple scaffolds, the model for each part was checked and documented for exon start and end phases at the break points, to make sure that the proper reading frame is obtained when concatenating all parts into a single model. Lastly, split models were documented as such both in the metadata and in the model name (labeled with the suffix '–part *x* of *y*'). As the gff3 format does not provide a way to specify this information other than in the comments section, the official gene set gff files contains multiple models per split gene, and the details for putting these parts together were documented separately (see Table S 4.2).

After the manual curation stage, the official gene set (OGS) was created by merging the computationally predicted gene set and the manual curated models. This was done using a 'patch' build system that uses heuristics to merge manual and automated gene predictions [45], whereby all automated models that overlapped on the same strand with manually curated models were replaced. This overlap was restricted to coding exons, and therefore it was important to take care that all reading frames were set correctly: if the reading frame of an upstream exon was incorrectly

set and generated a premature stop codon, the rest of the gene model downstream would be registered as untranslated region and all intersecting automated annotations would be kept in the OGS. Indeed, several of these cases were registered in the OGS v1.1, and corrected in v1.2. The OGS v1.1 [41] was used for downstream pipeline analyses and included manual curation of 1,426 models (194 *de novo*) for 1,201 genes, including multiple isoforms and the split gene models, among a total of 19,690 gene models for 19,465 unique genes.

The manual curation process was then resumed to make further improvements based on analysis of the OGS v1.1. These revisions affected 337 models (206 revised, 122 added *de novo*, 9 deleted). Almost half of these models represent the comprehensive addition of chemoreceptor genes of the ionotropic and odorant receptor classes, including a further 96 *de novo* models that were absent from the OGS v1.1. Among the remaining revisions were 52 models (13 *de novo*, 39 updated) for various metabolic enzymes. The annotation sets were merged again to produce the OGS v1.2 (Tables S 4.1, 4.2). After each merge, the annotation sets were screened in a quality control step to ensure that curated functional annotations were present and without formatting errors. The OGS v1.2 is the version that was submitted to NCBI, and both OGS datasets are available here: https://i5k.nal.usda.gov/data/Arthropoda/oncfas-(Oncopeltus_fasciatus)/Current%20Genome%20Assembly/2.Official%20or%20Primary%20Gene%20Set/.

To quantify the main curation actions that were performed during the manual curation effort, we ran the program gff-cmp-cat (https://github.com/chienyuehlee/gff-cmp-cat) on the curated models from OGS v1.2 and the original Maker dataset. This program classifies curation actions applied to a gene model by calculating differences between two overlapping models (see Supplementary Table S5 in McKenna, *et al.* [46] for definitions of how each action is calculated). A comprehensive list of the curated gene models and their curation actions is available in Table S 4.3. Out of 1697 curated gene models, 1159 had some structural modification or were newly added, while 359 gene models were approved without editing and were given functional annotations only (Table S 4.4). Strikingly, the number of genes resulting from a merged CDS action (205) is far greater than the number of gene models resulting from split CDS actions (30). These numbers suggest that the original Maker dataset may be an overestimate of the actual number of genes from certain gene families, and

that gene length was also underestimated. Indeed, the mean gene locus size of the OGS v1.1 is 12,985 bp (median: 8,819 bp), which is slightly higher than in the original automated set, because manually annotated genes were longer (mean: 20,794 bp; median: 11,326 bp). In an extreme example, the *Oncopeltus* orthologue of *hemocytin* (also known as *hemolectin*), which encodes a conserved carbohydrate binding protein of 3667 amino acids, was split across three scaffolds and predicted in ten separate automatic annotation models.

In addition, many more gene models were extended than reduced (376 vs. 215), in part due to correction of gene structure. The mean number of introns was lower in the OGS v1.1 compared to the automated predictions, because often the MAKER pipeline inserted very small introns within exons, where no RNA-seq supported junctions were evident. Indeed, the minimum intron length in the OGS v1.1 was 5 bp in an automated annotation, while the smallest intron supported by RNA-seq evidence was 71 bp, belonging to a manual annotation model for the gene *FK506-binding protein 15-like*.

These trends may be due to the fragmented nature of the assembly – genome assembly fragmentation is often associated with a higher predicted gene number and fewer exons per gene [44]. Furthermore, many gene models were newly added in the curation process (285), 70% of which are chemoreceptors (gustatory receptors, ionotropic receptors, and odorant receptors, Table S1; chemoreceptors represented only 4% of all curated genes), which are fairly small, rapidly evolving, and often lack expression support, making them difficult to predict computationally [see also. 46].

Table S 4.1: List of all curated models in the OGS v1.2.

Table S 4.2: Gene models split across scaffolds in the *Oncopeltus* genome assembly: correspondence of gene model IDs, and information on the models (in Excel supplement file).

Table S 4.3: A comprehensive list of all curated genes in OGS v1.2, including modification actions used to generate the gene model (in Excel supplement file).

Table S 4.4: A summary of curation actions by feature type in OGS v1.2, as computed by the program gff-cmp-cat. Note that the program does not handle rRNA features, so these actions were added manually. Descriptions of the curation actions and how they are calculated are presented in Table S5 of McKenna, *et al*. [46].

**Action type**	**gene/ pseudogene**	**mRNA**	**pseudogenic transcript**	**rRNA**
Added Models	298	316	13	
Extended Models	376	416		
Reduced Models	215	255		1
Models modified within boundary coordinates	58	62		
Models resulting from merged CDS	205	229		1
Models resulting from split CDS	30	43		
Models resulting from merged UTRs	30	34		
Models resulting from split UTRs	12	14		
All structurally modified models	1159	1307	13	2
Models with only functional annotations	359	375		
**Total number of manually curated gene Models**	**1518**	**1682**	**13**	**2**

5. Curation and comparative analysis of specific gene families

5.1 Developmental regulation: transcription factors and signaling pathways

One of the main reasons for choosing to sequence the *Oncopeltus* genome was due to its status as an experimental model system for developmental studies. For this reason, it was of special interest to analyze its developmental gene complement. In total, over one-third of all curated gene models (672 models, 40.4% of OGS v1.2) are of genes known to be involved in developmental processes and signaling pathways in other insects, with many of these encoding transcription factors. (Note that genes encoding proteins involved in the endocrinological control of postembryonic development and specifically in neurogenesis are addressed in separate sections below.)

5.1.a Anterior-posterior body axis: terminal patterning system and segmentation

Contributors: Peter K. Dearden, Andrew G. Cridge, Elizabeth J. Duncan, Megan Leask, Mackenzie K. Lovegrove, Olivia Tidswell, Ariel D. Chipman, Barbara M.I. Vreede, Neta Ginzburg

Early patterning genes appear mainly conserved relative to what is known from other insects, with very few instances of lineage-specific duplications in *Oncopeltus*, which are highlighted below. In cases where no ortholog was identified we remain cautious. It is already known that several key genes involved in axis formation in *Drosophila* are not conserved in other insects [47]. In other cases apparent gene absence may be due to genome assembly limitations, and readers are encouraged to also examine available transcriptomic resources (summarized in main text Fig. 2; [48]).

The terminal patterning system has been previously studied in *Oncopeltus* [49], and most of the relevant genes have been identified there. The genome analysis revealed a duplication of *Torso-like*, which encodes a perforin-like protein. *Torso-like* is also duplicated in the aphid genome, with one quite derived copy (*Torso-like related*), but the *Oncopeltus* duplications are independent of the aphid ones (based on Bayesian phylogenetics), and less derived. These copies have been named *Torso-like 1* and *Torso-like 2* to reflect a recent duplication with similar copies. The genome analysis did not recover a copy of *trunk*, which is involved in terminal patterning in *Drosophila*. However, we have recovered a copy of the closely related *PTTH*, which controls developmental timing of juvenile stages. In aphids and crustaceans, genes similar to *trunk/PTTH* are found with more similarity to vertebrate and lophotrochozoan *noggin*. A representative of this class of genes is also present in *Oncopeltus* (see below in Section 5.1.i). We have confirmed the absence of *torso*, reported to be missing in Weisbrod, *et al*. [49]. This gene encodes a receptor tyrosine kinase, of which many are present in the genome. The most closely related receptor tyrosine kinase we have found is most similar to 'Neurospecific receptor kinase'. This is a surprising result, since in all species studied so far where there is a ligand (either PTTH or trunk) there is also a receptor similar to Torso [50]. These findings suggest that *Oncopeltus* uses PTTH to control developmental timing (as this appears to be an

ancestral trait in insects), but it appears as though this pathway is not involved in terminal patterning [49, 50].

The genes known from the *Drosophila* segmentation cascade (maternal, gap, and pair-rule members) were examined in the *Oncopeltus* genome. With the exception of the cyclorrhaphan-restricted *bicoid*, homologs were found for all inspected candidate genes, including two copies each of *knirps* (previously characterized in [51]) and of *paired* class genes, and with expression support for two isoforms each of *nanos* and *Giant*. Interestingly, the gene reported as *engrailed* and used in several reports of *Oncopeltus* development [49, 52-54] turns out to be orthologous to *invected*, which encodes a diagnostic "RS-motif" from a single, small internal exon [55], and which we could validate empirically by amplification from cDNA. The actual *engrailed* ortholog occurs in a tail-to-tail orientation on the same scaffold.

Lastly, all three *odd*-like genes (*odd-skipped* (*odd*), *brother of odd with entrails limited* (*bowl*), and *sister of odd and bowl* (*sob*)) were identified in the *Oncopeltus* genome. However, simple recursive BLAST was not sufficient in identifying each ortholog individually; thus, additional care based on protein structure and conserved domains was taken in gene model curation and orthology assignments for the *odd*-like genes (Figure S 5.1).

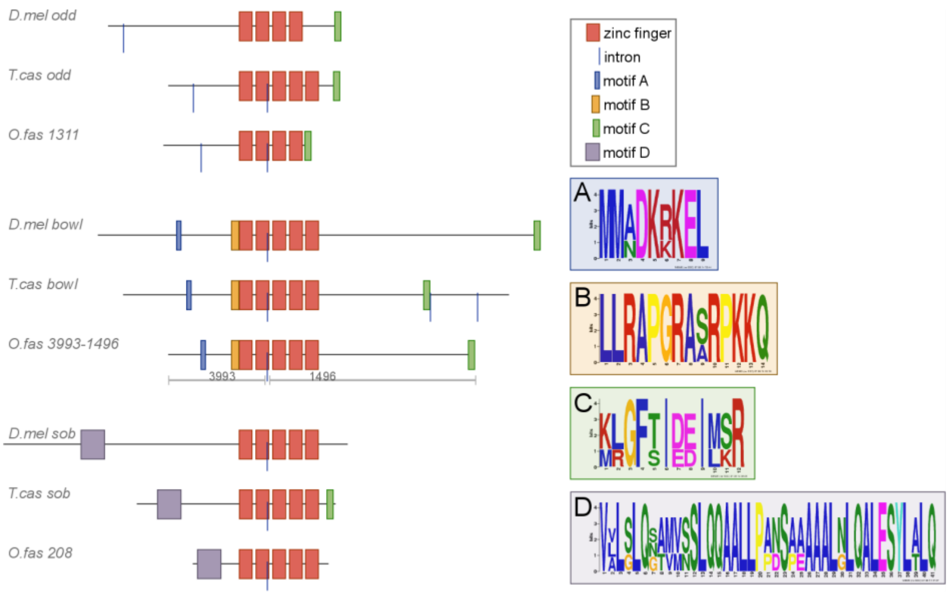

Figure S 5.1: Schematic of the coding DNA for *odd*-like genes *odd-skipped* (*odd*), *brother of odd with entrails limited* (*bowl*), and *sister of odd and bowl* (*sob*) in genomes of *Drosophila melanogaster*, *Tribolium castaneum*, and *Oncopeltus fasciatus*. *O. fasciatus* homologs were identified using a combination of intron locations, zinc finger motifs, as well as other key conserved motifs, outlined in A-D.

5.1.b Hox and other homeobox transcription factors

Contributors: Kristen A. Panfilio and Iris M. Vargas Jentzsch

Homeodomain transcription factors are a protein superfamily with diverse roles in developmental regulation, with the eponymous Hox genes representing a textbook case of metazoan-wide conservation in gene cluster organization, protein sequence, and role in tissue specification along the anterior-posterior body axis [56]. Between pipeline analyses of OGS v1.1 (see main text and Section 6.2, below) and manual curation, we have identified 96 genes encoding homeodomain transcription factors, of which 39 have been manually curated for their relevance to specific biological roles covered here and in other manual curation sections. For the Hox genes, we have found and annotated complete, single copy orthologs for all ten expected genes in *Oncopeltus* (Table S 5.1). The Hox cluster is, however, fragmented and distributed across 11 scaffolds in the current assembly: only *zen* and *Dfd* are linked on the same scaffold, while *Scr* and *Ubx* are each split across two scaffolds (Figure S 5.2 A).

The size of the complete Hox cluster, assuming a direct concatenation of these 11 scaffolds, is 4.2 Mb. This is relatively large compared to the better-assembled clusters we annotated in other i5K genomes: in both the bed bug (*Cimex lectularius*, [19]) and the Asian longhorned beetle (*Anoplophora glabripennis*, [46]) the cluster spans 3.5 Mb, assembled onto only one or two scaffolds, respectively. Both species have genome sizes comparable to *Oncopeltus* (926 Mb), from 865 Mb in the bed bug to 976 Mb in the Asian longhorned beetle. The increase in size of the *Oncopeltus* cluster is largely due to an increase in the length of intronic and intergenic regions, including gaps in the assembly. In contrast, protein sizes are marginally smaller (see Table S 5.2). This reduction in protein size was more prominent for the anterior four genes (*lab*, *pb*, *zen*, and *Dfd*). Splice sites in the central and posterior genes (*Dfd*, *Scr*, *Antp*, *Ubx*, *abd*-A and *Abd*-B) are well conserved.

For the functionally diverged Hox gene *fushi tarazu (ftz)*, a single transcript sequence was identified in a previous transcriptome [48]. Based on empirical analyses by Yong Lu (group of Leslie Pick, unpublished data), two transcript isoforms were annotated for *ftz* in the current genome assembly. However, while the presumptive homeodomain open reading frame could be found, neither isoform appears to encode

a complete transcript for a functional protein, and these two isoforms may rather represent degradation to pseudogene status.

Beyond the Hox genes, we were also able to identify clear orthologs in conserved syntenic pairs for the aforementioned *engrailed* and *invected* paralogs (see Section 5.1.a) as well as the Iroquois Complex pair *iroquois* and *mirror*, which retain the conserved organization of shared transcriptional orientation from the same DNA strand (Figure S 5.2 B). Please see below (Section 5.1.c) for further details on Iro-C gene copy number evolution.

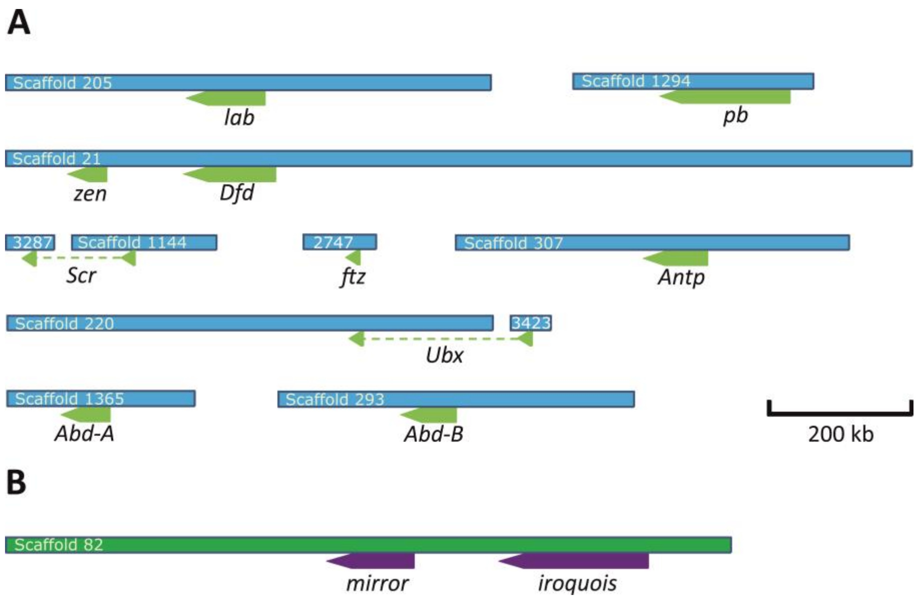

Figure S 5.2: Schematic representations of the Hox cluster **(A)** and the Iroquois Complex **(B)**, with transcriptional orientation as indicated and shown to scale for gene loci and total scaffold lengths.

Table S 5.1: Summary information for the Hox cluster genes.

Gene	Scaffold: start..end	Locus length (nt)	Number of CDS exons	Protein length (aa)
labial	Scaffold205:185231..267231	82,001	3	273
proboscipedia	Scaffold1294:17732..184912	167,181	3	571
zerknuellt	Scaffold21:1100766..1120884	20,119	4	198
Deformed	Scaffold21:161709..839714	678,006	2	331
Sex combs reduced	Scaffold1144:107131..107896<br>Scaffold3287:29411..30383	123,099[1]	2	301
fushi-tarazu	Scaffold2747:19754..20621	868	0	-
Antennapedia	Scaffold307:282886..304398	21,513	2	301
Ultrabithorax	Scaffold3423:34350..35230<br>Scaffold220:445840..446672	259,972[1]	2	295
abdominal-A	Scaffold1365:78419..171923	93,518	3	334
Abdominal-B	Scaffold293:181929..221609	39,681	2	316

[1] Gene locus length estimate assumes direct concatenation of the annotation-containing scaffolds.

Table S 5.2: Comparison of Hox proteins between the beetles *Tribolium castaneum* (Tcas) [57] and *Anoplophora glabripennis* (Agla) [46], and the bugs *Oncopeltus fasciatus* (Ofas) and *Cimex lectularius* (Clec) [19]. Percent protein size change is relative to *Tribolium*.

	Tcas		Agla			Ofas			Clec		
**Hox gene**	**protein length**	**# CDS exons**	**protein length**	**% protein size change**	**# CDS exons**	**protein length**	**% protein size change**	**# CDS exons**	**protein length**	**% protein size change**	**# CDS exons**
lab	353	2	384	1.09	2	273	0.77	3	279	0.79	2
pb (mxp)	654	3	717	1.10	4	571	0.87	3	601	0.92	3
zen (zen1)	246	3	319	1.30	2	198	0.80	4	307	1.25	3
Dfd	412	2	454	1.10	2	331	0.80	2	337	0.82	2
Scr (Cx)	312	2	336	1.08	3	302	0.97	2	323	1.04	2
ftz	290	2	389	1.34	2	-	0	0	235	0.81	2
Antp (Ptl)	325	2	327	1.01	2	301	0.93	2	302	0.93	2
Ubx	314	3	296	0.94	2	295	0.94	2	301	0.96	2
abd-A	343	3	365	1.06	3	334	0.97	3	345	1.01	3
Abd-B	351	2	388/400	1.14	2	316	0.90	2	361	1.03	3

5.1.c Iroquois Complex (Iro-C) cluster

Contributors: Jan Seibert and Kristen A. Panfilio

The transcription factors *araucan* (*ara*), *caupolican* (*caup*), and *mirror* (*mirr*) belong to the TALE superclass of homeodomain proteins [58] and form the Iroquois-Complex (Iro-C) in the fruit fly *Drosophila melanogaster* [59-61]. It is already known that *ara* and *caup* arose due to a tandem duplication of the gene *iro* in the drosophilid lineage [62], while the Iro-C itself is ancestral within crustaceans and insects. In vertebrates on the other hand, one can find 2-4 Irx clusters, with up to three genes per cluster. More basally branching metazoans like the cnidarians or the placozoans, but also nematodes, which as fellow Ecdysozoa are much closer to insects, have only a single Irx gene [63]. Given these deep, lineage-specific complements of Irx genes and clusters, they provide a perfect target to assay the quality of the *Oncopeltus* genome.

The search for genes of the Iro-C in *O. fasciatus* revealed again its conserved synteny (Figure S 5.2 B, previous section; see also Methods, below, and Table S 5.3, Figure S 5.3). As in *D. melanogaster* and *Tribolium castaneum*, in *O. fasciatus* the *iro* gene (the single *ara*/*caup* ortholog) and *mirr* are transcribed from the same DNA strand, with *iro* situated 5′ to *mirr*, in our genome assembly (Scaffold 82). Phylogenetic analyses of Iro-C protein sequences from representative species within the insects confirmed the expected relationship of *O. fasciatus* Iro and Mirr with orthologous proteins of the other species (Figure S 5.4). Looking at other predicted gene models within i5K species, *iro* and *mirr* could also be found in the bed bug *Cimex lectularius* (Scaffold 47, [19]) and the Asian longhorned beetle *Anoplophora glabripennis* (Scaffold 135, [46]) with conserved synteny.

In most bilaterians, but not in vertebrates, this synteny can be extended to the ankyrin repeat-containing *sosondowah* (*sowah*) gene, which is known to be associated with the Iro-C [61, 64]. In all so far investigated insect species *sowah* is located 5′ to the Iro-C and has an opposite reading direction [63].

In our analyses, *sowah* is found on Scaffold 1078, so it is unclear if the synteny is conserved in *O. fasciatus*. An indirect hint could be that also *ceramide transfer protein* (*cert*) is found in the same reading direction on Scaffold 1078. *cert* is in *D. melanogaster* located on chromosome 3L, together with *sowah* and all three Iro-C genes. So the fact that both genes have the same reading direction and the same

transcriptional orientation in the milkweed bug compared to the fruit fly might indicate conserved synteny.

Our results not only support the high degree of conservation of the Iro-C and its associated genes in *Oncopeltus fasciatus*, they also strengthen the quality of our assembly in general with the expected linage of the Iro-C gene pair.

Methods

Iro-C and Irx protein sequences were retrieved using TBLASTN and BLASTP algorithms on the corresponding databases/genome browsers (Table S 5.3) for selected species (Figure S 5.3). The sequences of the three Iro-C genes in *Drosophila melanogaster* served as queries in all cases. Predicted gene models within the *Oncopeltus fasciatus* assembly were manually curated to obtain the full sequence.

The phylogenetic relationships of the cladogram (Figure S 5.3) were first obtained via NCBI's taxonomy browser, refined with published findings [65], and rebuilt and edited with the free software INKSCAPE.

The final tree (Figure S 5.4) was built with the free software MEGA 6 [66], using ClustalW for the alignment and Maximum Likelihood for phylogenetic tree construction. For the analyses preferences the default settings were used, except the number of bootstrap replications, which was set to 1.000.

Table S 5.3: Iro-C homolog protein sequence sources.

**Species name**	**Abbreviation**	**Source database**	**ID's for *araucan/ caupolican***	**ID for ortholog of *iroquois***	**ID for ortholog of *mirror***
Bombyx mori	*Bmor*	NCBI	-	XP_004929820.1	XP_004929953.1
Drosophila melanogaster	*Dmel*	NCBI	AAF49896.1/ AAF49895.1	-	AGB94471.1
Nasonia vitripennis	*Nvit*	NCBI	-	XP_008216675.1	XP_001604937.1
Oncopeltus fasciatus	*Ofas*	i5k	-	OFAS025321	OFAS001761
Tribolium castaneum	*Tcas*	Beetle Base	-	TC003632	TC003634
Trichinella spiralis	*Tspi*	NCBI	-	XP_003372666.1	-

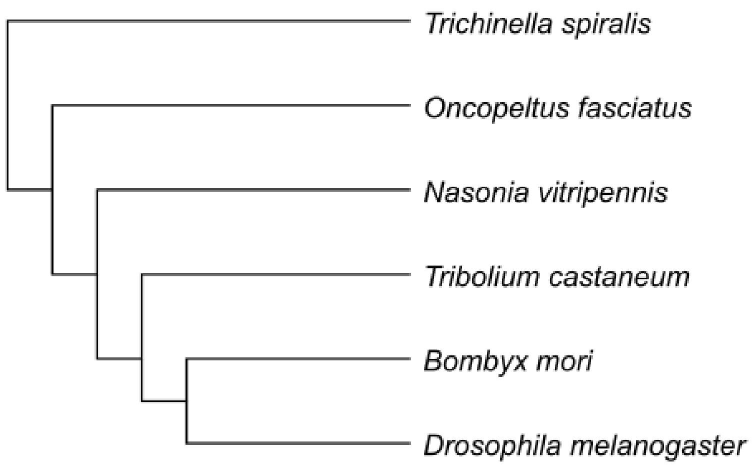

Figure S 5.3: Cladogram of species, based on the NCBI Taxonomy Browser, with the nematode *Trichinella spiralis* providing an ecdysozoan outgroup to selected insects.

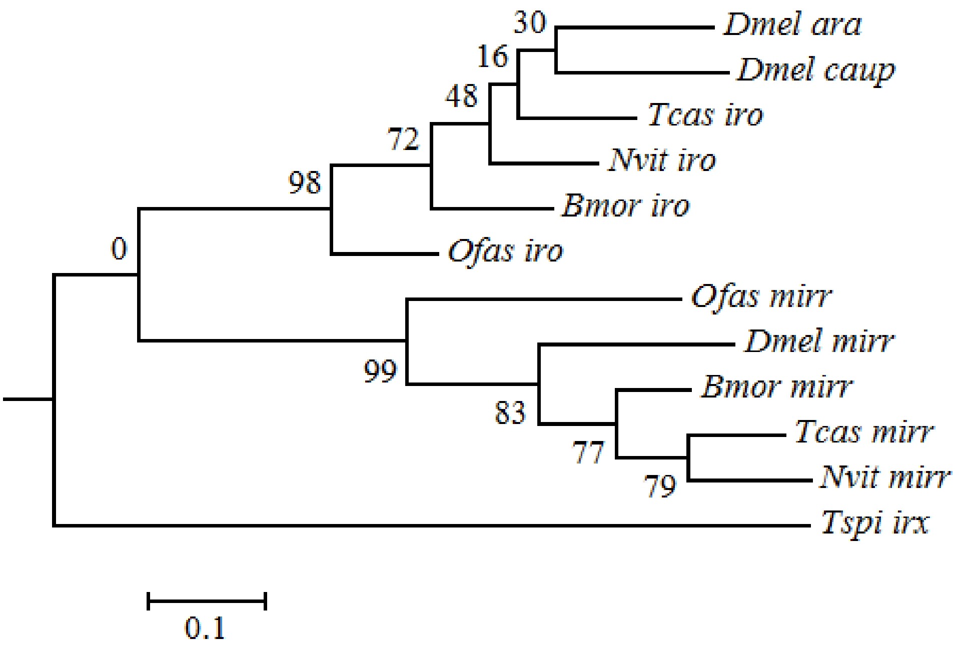

Figure S 5.4: Maximum likelihood phylogenetic analysis of insect Iro-C proteins. The two branches of the two (three in *D. melanogaster*) Iro-C genes have an excellent support value, putting *O. fasciatus mirror*, as well as *iroquois* in the corresponding group. *D. melanogaster araucan* and *caupolican* group together, like one would predict after gene duplication. Notably the support values for this grouping and already for those in the *iroqouis* branch starting at *N. vitripennis* are quite low.

5.1.d T-box transcription factors and heart determinants

Contributors: Thorsten Horn and Kristen A. Panfilio

We found 5 T-box genes in *Oncopeltus* based on homology to *Drosophila melanogaster*, *Tribolium castaneum* and additional species where available (Figure S 5.5). This is fewer than in *Drosophila* (8) and *Tribolium* (6). However the gene *Dorsocross* has three paralogs in *Drosophila* (*Dmel-Doc1-3*), which stem from a recent duplication in the *Drosophila* lineage and possess similar expression patterns and functions [67]. Therefore, we would only expect 6 T-box genes in *Oncopeltus*, similar to *Tribolium*. The only missing T-box gene in *Oncopeltus* is *optomotor-blind-related-gene-1* (*org-1*). We did find a partial duplication of *Ofas-optomotor-blind* (*Ofas-omb*) on Scaffold 195, but as the genomic sequence of both regions is exactly the same, we rather suggest an assembly error than a hint towards *Ofas-org-1*. *Ofas-omb* consists of 11 exons (7 in *Drosophila*, 4 in *Tribolium*) located on two scaffolds and is probably complete on the CDS level. *brachyenteron* (*byn*), the insect homologue of *brachyury* (the founding member of the T-box gene group), was found in 8 exons (7 in *Drosophila*, 4 in *Tribolium*), which probably do not represent the full CDS.

All three T-box genes important for heart development in *Drosophila* (*midline* (*mid*), *H15* and *Doc*) [68] were found, as were other highly conserved transcription factors involved in heart development (the homeobox gene *tinman* and the zinc finger class gene *pannier*: Table S 5.4). *Ofas-mid* is split across two scaffolds and consists of 5 exons (4 in *Drosophila*, 6 in *Tribolium*). *Ofas-H15* consists of 5 exons (6 in *Drosophila*, 5 in *Tribolium*). The protein is slightly smaller than in *Drosophila* and *Tribolium* and the 5' UTR is missing. The orthologs of *Ofas-H15* and *Ofas-mid* are neighboring genes in both *Drosophila* and *Tribolium*, but in the current *Oncopeltus* assembly they occupy separate, relatively small scaffolds. Further improvement of the assembly will show if they are also linked in *Oncopeltus*. Homology assignment was not possible based on *Drosophila* homologues (Figure S 5.5), but is supported by *Tribolium* and additional species (not shown).

Based on homology and experimental RACE-PCR data, *Ofas-Doc* could be identified scattered across 4 scaffolds, which when put together probably constitute the complete gene model. The gene consists of 6 exons (5-7 in *Drosophila* and 4 in

Tcas). Of 4 splice sites within the coding region, 3 are conserved with *Drosophila* and 2 are conserved with *Tribolium*. The overall length seems comparable between *Oncopeltus*, *Drosophila* and *Tribolium*. Although *Doc* plays an important role in extraembryonic development in *Tribolium* and *Drosophila* [69], its role in heart development does not seem to be conserved in *Tribolium* [69]. Functional investigations of this gene in *Oncopeltus* will facilitate our understanding of the evolution of T-box transcription factors in insects.

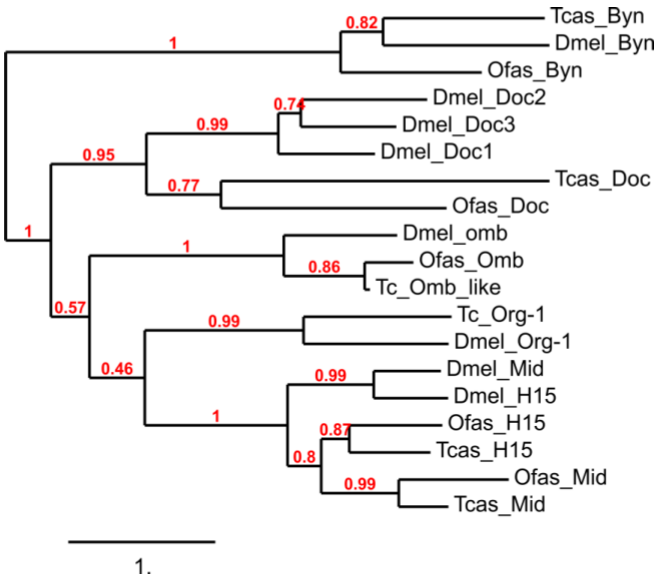

Figure S 5.5: T-box transcription factors in *Drosophila*, *Tribolium* and *Oncopeltus*. Maximum likelihood tree generated from protein sequences with default parameters without Gblocks at http://www.phylogeny.fr, based on [42, 43].

Table S 5.4: Manual curation summary of potential dorsal and mesodermal determinants.

Gene	Scaffold: start..end	Locus length (nt)	# CDS exons	Protein length (aa)
tinman	Scaffold212:18752..21136	2,385	2	128 (partial)
pannier	Scaffold2229:82858..97807	14,950	3	208 (partial)
dorsocross	Scaffold7:450646..450744<br>Scaffold6818:378..503<br>Scaffold1378:36101..37197<br>Scaffold7:510006..512170	99<br>126<br>1,097<br>2,165	1<br>1<br>2<br>2	319
midline	Scaffold776:16160..18303<br>Scaffold2277:68612..109253	2,144<br>40,642	2<br>3	312
H15	Scaffold679:74406..129278	54,873	5	296
optomotor-blind	Scaffold111:653518..772820<br>Scaffold195:155269..184484	119,303<br>177,148	2<br>9	636
brachyenteron	Scaffold262:13930..46676	32,747	8	292 (partial)

5.1.e Nuclear receptors

Contributors: Yong Lu and Leslie Pick

The nuclear receptor (NR) family of transcription factors is one of the largest transcription factor families, functioning in diverse biological processes including homeostasis, cell proliferation, reproduction and development [reviewed in 70, 71-76]. Many NRs function as ligand-responsive transcription factors whose activity is regulated by the binding of small molecule ligands (such as ecdysone, see below in Section 5.2.b). However, for other NRs, no ligand has been identified to date and many of these orphan NRs are likely regulated by other mechanisms. In fact, NRs appear to be evolutionarily flexible transcription factors with gain and loss of modes of regulation. Changes in ligand binding, protein-protein interaction and post translational modifications all play roles in their cooption into new biological roles, perhaps explaining their maintenance in animal genomes [77-79]. The NRs have been parsed into 5 large families that are distinguished by their requirements for ligand as well as by the structure of their ligand binding domains [80]. Both ligand-activated and orphan nuclear receptors share a similar, modular protein structure comprised of a variable N-terminal region, a highly conserved zinc-finger DNA binding Domain (DBD), a second variable hinge region, and a less conserved Ligand Binding Domain (LBD) that includes the activation function-2 (AF-2) domain (Table S 5.5, Figure S 5.6). For ligand-regulated NRs, the role of ligand binding is to induce a conformational change that repositions the AF-2 domain, releasing corepressors and creating a surface for coactivator binding, thus switching the NR between repressive and activating states [81]. Orphan nuclear receptors are activated by protein-protein interactions and post-translational modifications with, at least in several cases, the LBD folding in an active conformation in the absence of ligand [82, 83]. In *Drosophila*, 18 classic NRs have been identified [84]. Eight of these NRs are involved in ecdysone signaling pathways. The other NRs participate in a variety of processes including embryonic development, differentiation and metabolism. Orthologs of six of these NRs were identified in the *Oncopeltus* genome: *HR96, HNF4, HR78, Tailless, Dissatisfaction, HR51, Seven up,* and *HR38* (Table S 5.5). The two *Drosophila* NRs not found in the *Oncopeltus* genome are ERR and HR83. In *Drosophila*, ERR was found to be important for carbohydrate metabolism [85, 86]. The function of HR83 in *Drosophila* is not clear. According to FlyBase, two classic

HR83 alleles do not show any obvious phenotype and the flies are fertile. However, because the current *Oncopeltus* genome is still divided among many scaffolds, it is possible that these genes were missed in our annotation. The *Anopheles*, *Tribolium* and *Apis* genomes all have 18 NRs, with *Aedes* missing HR83 [57, 87, 88]. Thus, the family of NRs appears to be highly conserved in hemi- and holometabolous insects.

Overall, of the 16 nuclear receptors that were found in the *Oncopeltus* genome (Table S 5.5), Seven Up retains the highest degree of similarity to its *Drosophila* ortholog, with its DBD 100% identical to that of the *Drosophila*, and its LBD 94% identical to that of the *Drosophila*. On Scaffold 1872, there is a predicted NR (OFAS016187-RA), most likely an ortholog of HNF4, that may have resulted from retrotransposition of a processed NR transcript, as it is comprised of a single exon. This gene would encode a protein of 470 aa; the DBD of the predicted protein shares 79% identity with that of *Drosophila HNF4*, and the LBD shares 60% identity with that of *Drosophila HNF4*; and it has expression support from both embryonic and post-embryonic transcriptomes. For the 16 NRs identified, all appear to be present in single copy in the *Oncopeltus* genome. This is similar to the situation in other insects, including *Drosophila*, *Anopheles*, *Aedes*, *Tribolium* and *Apis*. Finally, aside from OFAS016187-RA, no novel NRs were identified in the *Oncopeltus* genome, although species-specific NRs may still be revealed.

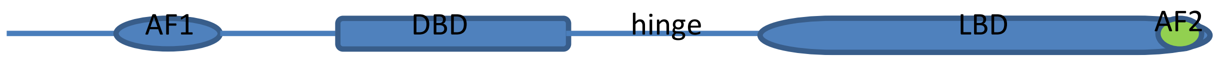

Figure S 5.6: Common structure of nuclear receptors: AAF1 (Activation Function 1), DBD (DNA Binding Domain), Hinge, LBD (Ligand Binding Domain), and AF2 (Activation Function 2) located within LBD.

Table S 5.5: List of identified *Oncopeltus* nuclear receptors (NRs) (in Excel supplement).

5.1.f Dorsal-ventral body axis: BMP/TGF-β pathway

Contributors: Lena Sachs and Siegfried Roth

The BMP pathway consists of extracellular ligands and their modulators, of transmembrane receptors with intracellular serine/threonine kinase activity and of cytoplasmic signal transducers, which relay the signal to the nucleus and regulate target genes [89]. All core components of the BMP signal pathway were found in the *Oncopeltus* genome (for functional analysis of key components see [90]). These include orthologs encoding: the two typical BMP ligands Decapentaplegic (Dpp, a BMP2/4 ligand) and Glass bottom boat (Gbb, a BMP5/7 ligand), the type I receptors Thickveins (Tkv) and Saxophone (Sax), and one type II receptor (Punt). Regarding cytoplasmic signal transducers we found one homolog of Medea (Co-Smad/Smad4). However, we found three paralogs for Mad *(*Smad1/5), two of them occurring on the same scaffold in close proximity with inverse orientation, which likely represents a recent duplication.

In *Drosophila* two additional transcription factors, Schnurri and Brinker, have been identified that act together with Medea/Mad to regulate many BMP target genes [91]. Both are present in genomes of other holometabolous insects. We found homologs of each in the *Oncopeltus* genome. The gene model for *brinker* is supported by transcriptome data ([48]). Potential orthologs of *brinker* are also present in the bed bug and pea aphid (41% and 39% amino acid identity, from the GenBank "uncharacterized protein" accessions XP_014250940.1 and XP_003240098.1, respectively). A deep conservation of *brinker* in insects is interesting, as there are no clear *brinker* homologs in other arthropods or other animal phyla. However, ongoing *brinker* evolution may be suggested by the fact that whereas the *Oncopeltus*, pea aphid, and *Drosophila* orthologs each encode a protein with a single Brinker DNA-binding domain (BrkDBD: Pfam ID PF09607), existing protein accessions from both the bed bug and *Tribolium* each contain two BrkDBD's.

The activity of BMP receptors and cytoplasmic signal transducers are subject to secondary modulation. An interesting case is the pseudoreceptor BAMBI, which has been well studied in vertebrates and acts as a negative regulator of BMP signaling [92]. BAMBI has been lost in the lineage leading to *Drosophila*, however it is present in *Tribolium* and *Nasonia* [93, 94]. We found a homolog in *Oncopeltus* (incomplete

gene model). Homologs also exist in *Halyomorpha* and *Cime*x. Modulation of the BMP receptors and SMADs occurs also at the level of regulated protein degradation. In *Drosophila* the E3 ligase Smurf regulates ubiquitination and proteolysis of the BMP receptor Thickveins and/or pMAD [95, 96]. A Smurf ortholog was found in *Oncopeltus*.

Oncopeltus possesses a typical set of extracellular modulators of BMP signaling, including: a homolog of the BMP inhibitor Short gastrulation (Sog); a homolog of Tolloid (Tld), the protease that cleaves Sog to release the BMPs from Sog-BMP complexes; Crossveinless-1 (Cv-1, also known as Twisted gastrulation (Tsg)); Crossveinless-2 (Cv-2); and Pentagone [89, 97]. However, no BMP modulators of the DAN family were discovered in the genome, although two are present in *Tribolium*, suggesting a lineage-specific loss [92, 94]. Similarly, no *admp* ortholog could be identified, similar to the situation in pea aphids, although one is present in the holometabolous wasp *Nasonia vitripennis* [93].

Interestingly, Noggin, a secreted BMP inhibitor that so far has not been identified in any of the holometabolous insect genomes [50], is present in *Oncopeltus*. Within the Hemiptera Noggin homologs show considerable sequence divergence, as the Noggin proteins of the bed bug and pea aphid show only 50% and 32% similarity to *Oncopeltus* Noggin, respectively.

5.1.g Dorsal-ventral body axis: Toll/ NFκB pathway

Contributors: Yen-Ta Chen and Siegfried Roth

The Toll pathway is involved in innate immunity, convergent extension and dorsal-ventral (DV) patterning in a wide range of insect species including *Oncopeltu*s and *Rhodnius* [Y.T. C and S.R. unpublished results, 90, 98, 99-101]. The focus of this annotation report is DV patterning.

The core Toll signaling cascade in insects comprises the Toll-like transmembrane receptors (TLRs), the intracellular adaptor Myd88, two serine-threonine kinases (Tube-like kinase and Pelle), the I-kappaB homolog Cactus and the NF-kappaB transcription factor Dorsal [102, 103].

We identified six TLRs in *Oncopeltus*. Toll1, Toll6, Toll7, Toll8, Toll10 were identified in both the genome and transcriptome [48] while Toll9 was only identified in the transcriptome. Toll6, Toll8 and Toll10 belong to the Loto clade of segmentally expressed Tolls [101]. Toll1 is involved in DV patterning and probably also in innate immunity [90].

As for the downstream cytoplasmic signal transducers we found one representative each for Myd88, Pelle and Tube-like kinase. The presence of the latter further supports the idea that Tube proteins fused to a kinase domain represent the ancestral state for insects, and consequently that the Tube proteins of Hymenoptera and higher Diptera lacking a kinase domain resulted from a secondary loss [103]. Surprisingly, we identified six paralogs for I-kappaB/*cactus* scattered throughout the genome (Figure S 5.7), four of which have been shown to be expressed during blastoderm stages [90]. (Note that these cactus paralogs, annotated as 'cactus-like', were added only for OGS v1.2.) Phylogenetic analysis shows that the increase in *cactus* paralogs is lineage-specific within Heteroptera, as *Cimex lectularius* and *Rhodnius prolixus* each have only one *cactus* gene. However, a tendency to duplicate *cactus* genes has been observed in other insect lineages, such as in the Hymenoptera, and is likely to be linked to complex immune functions of Toll/NF-kappaB signaling.

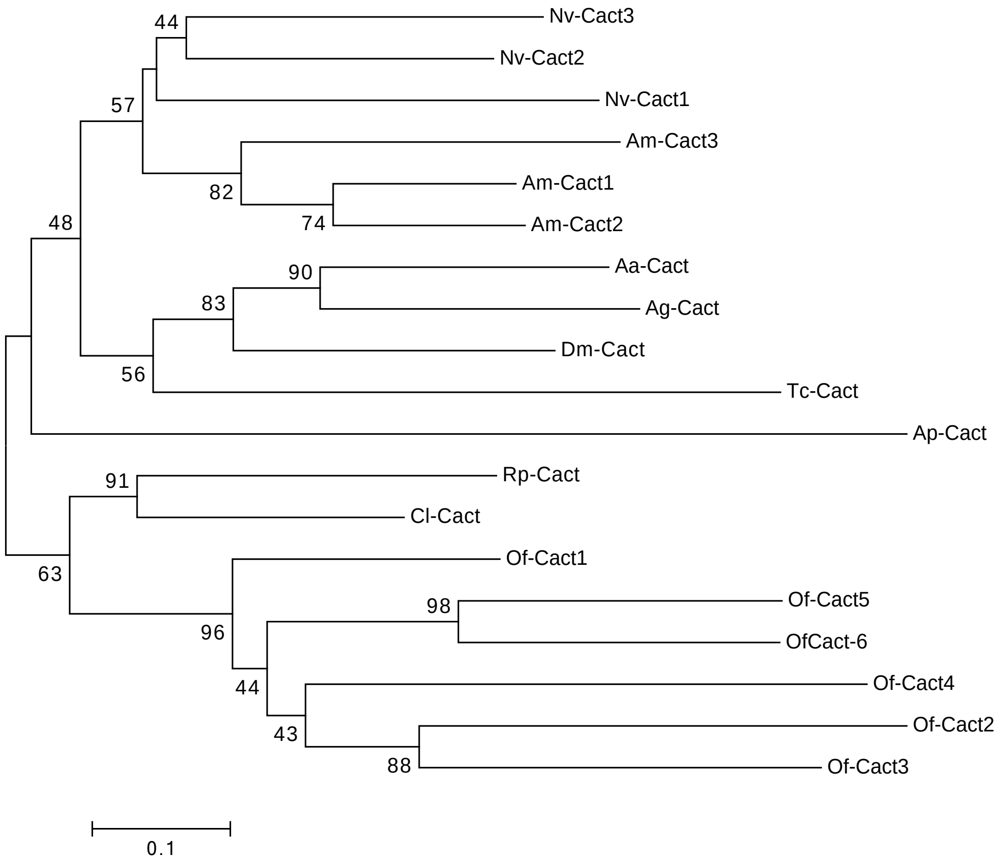

Figure S 5.7: Phylogenetic tree of *cactus* genes.

The optimal tree with the sum of branch length = 6.19659819 is shown. The percentage of replicate trees in which the associated taxa clustered together in the bootstrap test (1000 replicates) is shown next to the branches. The tree is drawn to scale, with branch length in the same units as those of the evolutionary distances used to infer the phylogenetic tree. The evolutionary distances were computed using the Poisson correction method and are in the units of the number of amino acid substitutions per site. The analysis involved 19 amino acid sequences. All positions containing gaps and missing data were eliminated. There were a total of 118 positions in the final dataset. Evolutionary analyses were conducted with MEGA7 [104].

The *Oncopeltus* genome contains two NF-kappaB/*dorsal* sequences resulting from a duplication which has occurred independent from the *dorsal* gene duplications found in holometabolous lineages and indeed is lineage-specific within the Heteroptera as *Cimex* and *Rhodnius* possess only one *dorsal* gene. Both *Of-dorsal* genes are on the same scaffold within a 45-kb interval and have the same orientation

suggesting that they have arisen by tandem duplication. Both dorsal genes contribute to DV patterning, albeit to a different degree [90].

Among the potential direct or indirect target genes of NF-kappaB/Dorsal [100, 105] we identified single copies of the following DV patterning genes: *short gastrulation* (see annotation of BMP signaling components), *twist* (*twi*), *snail* (*sna*), *single minded* (*sim*), *SoxNeuro* [106], and the three columnar neuroectodermal genes *muscle segment homeodomain* (*msh*), *intermediate nerve cord defective* (*ind*) and *ventral nervous system defective* (*vnd*) [107].

5.1.h Innate immunity

Contributors: Chris G.C. Jacobs, Yen-Ta Chen, Maurijn van der Zee

We were able to annotate 99 immune genes in the *Oncopeltus* genome. This number includes the Toll signaling components described in Section 5.1.g. Additionally, fifteen Defensins, seven lysozymes and five Hemiptericins were annotated, of which some have been previously identified [108]. Interestingly, no Cecropins, Attacins or Thaumatins were found. In addition, we found nine new potential antimicrobial peptides (AMPs), including three recently confirmed Serosins and two recently confirmed Ovicins (Submitted manuscript: Jacobs CGC, van der Hulst R, Chen YT, Roth S, van der Zee M. (2017) Innate immune function of the serosa in a hemimetabolous insect). This illustrates the need for species-specific AMPs for effective elimination of evolving pathogens. The Toll pathway is well conserved (see Section 5.1.g). Although IMD is missing from the hematophagous hemipterans *Rhodnius* and *Cimex*, IMD is present in the genomes of *Oncopeltus* and *Gerris*. However, classical cloning with degenerate primers was necessary to isolate the *Oncopeltus* ortholog. As for FADD and Tab2, gene models were absent from the genome assembly and coding sequences were even not found by tBLASTn, possibly because of extremely short exons (see also main text results and discussion on gene structure evolution and short exons within the Hemiptera). The sequences were finally found by *de novo* RNA-seq assembly (submitted manuscript cited above). IMD is also present in the closely related leaf hoppers *Nilaparvata* [109] and *Homalodisca*, suggesting that IMD was present in an ancestor of all Paraneoptera. Loss of IMD was reported from *Acyrthosiphon* [32]. IMD was also not found in the genomes of *Frankliniella*, *Pediculus*, *Diaphorina* and *Pachypsylla*. This could be a single evolutionary loss, if a close relationship of the Thysanoptera, Phthiraptera and Sternorrhyncha is assumed, as found for instance when using the BI-DNA method (see Figure S 5.8, [110]). However, the most accepted phylogeny suggests multiple losses. These losses could mean that the Toll pathway is not only able to upregulate effector genes in response to Gram-positive bacteria, but also in response to Gram-negative bacteria. Interestingly, response to these distinct bacterial inputs is also not strict in *Tribolium*, and to some extent also not in *Drosophila* [111-114].

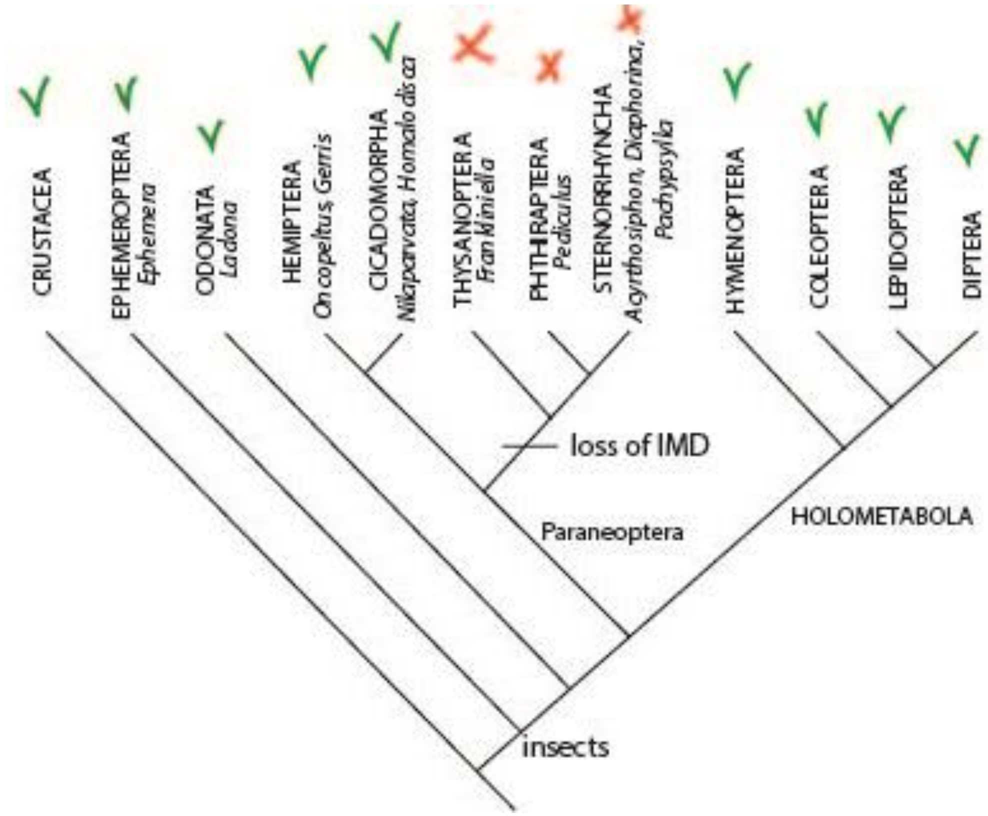

Figure S 5.8: Presence of IMD in major insect groups. Phylogeny from [110] based on BI-DNA method.

5.1.i Notch, Hedgehog, and Torso RTK pathways

Contributors: Peter K. Dearden, Andrew G. Cridge, Elizabeth J. Duncan, Megan Leask, Mackenzie Lovegrove, Olivia Tidswell.

Cell signaling pathways (Notch, TGFβ and Hedgehog) are, on the whole, conserved. Subtle differences in downstream genes likely reflect evolutionary distance from *Drosophila* (where these systems are well studied), rather than *Oncopeltus* specific biology. However, see above (Section 5.1.a) for details on the Torso Receptor Protein-Tyrosine Kinase (RTK) signaling pathway, which is involved in terminal patterning in the context of anterior-posterior axis specification, and for which we have conducted a phylogenetic analysis to assign *Oncopeltus* ligand orthology (Figure S. 5.9).

Previous work has indicated that conserved gene complexes are rare in insect genomes, and that only three are conserved over significant evolutionary time. One of these is the Hox complex (Section 5.1.b)**.**

A second conserved complex is the Runt complex. This set of four Runt domain containing genes is a feature of insect genomes, but has not yet been found in crustaceans [20]. Ancestrally (as seen in aphids and *Pediculus*) the complex contains 4 genes in a stereotyped order. In *Oncopeltus*, this order may be conserved. *Runt* and *RunXA* sit on the same scaffold, with no surrounding genes, and *lozenge* and *RunXB* sit on their own scaffolds with no surrounding genes. If these scaffolds were contiguous, then this would produce a conserved Runt complex. Additionally, a duplicated RunXA gene sits on Scaffold 2955.

The third complex is the Enhancer of split (E(spl)) complex, a feature of both insect and crustacean genomes [115]. This complex of genes is regulated by Notch signaling and has roles in cell fate specification. Ancestrally (as seen in *Daphnia* and aphids), this complex consists of four genes in stereotyped locations and is often associated with a paired-like gene at one end of the complex. In *Oncopeltus* this complex appears to have been broken apart, with only two E(spl) genes in the genome, both surrounded by other genes with homologs in other species, providing confidence that these are real genes. The two E(spl) genes are most closely related to E(spl) bHLH2 genes (annotated as E(spl) mBeta, Scaffold 744), and E(spl) bHLH1

genes (annotated as E(Spl) m7, Scaffold 857). No orthologues of *Her* or *malpha* (a bearded class gene found in the E(Spl) complex) can be found. This rearrangement is most likely derived given the conservation of the complex in crustaceans.

Other gene families analyzed include the *sp* genes, where we have identified orthologs of *sp4* and *sp8/9* and confirmed the absence of *sp5/buttonhead* [116, 117].

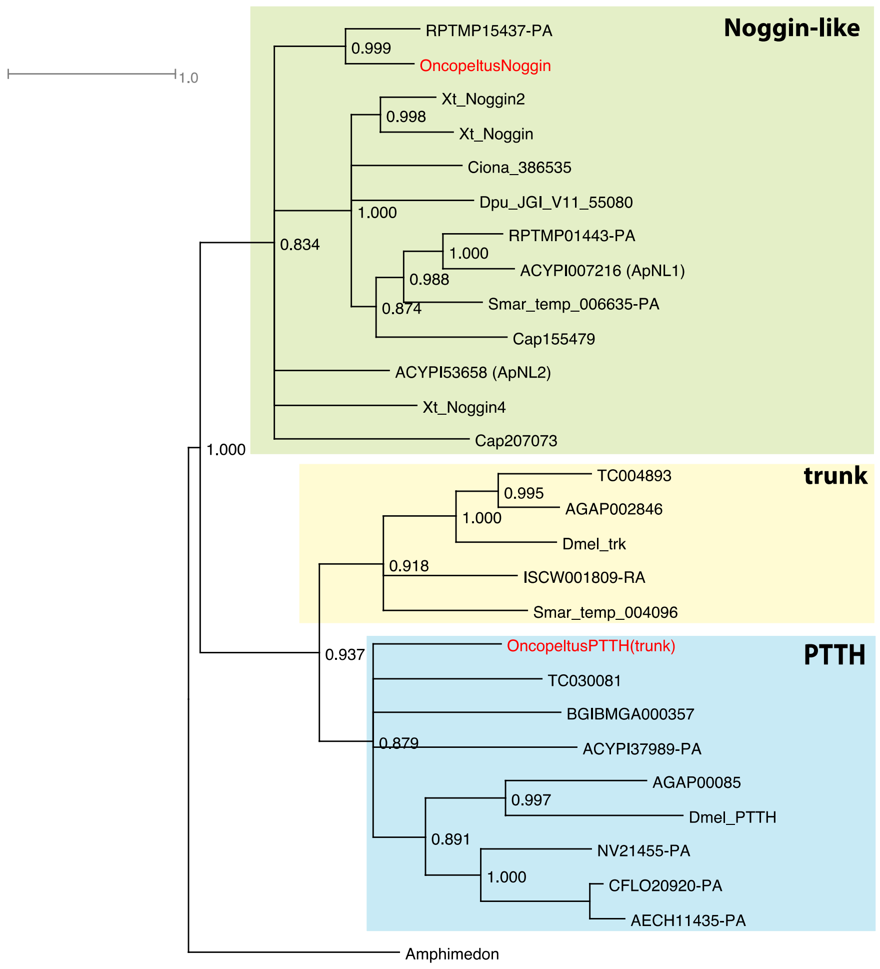

Figure S 5.9: Bayesian phylogeny of arthropod Noggin-like/PTTH/trunk family of proteins [50], including orthologs identified from the *Oncopeltus fasciatus* genome. Using a combination of methods, including BLAST homology searches and screening annotated *Oncopeltus* proteins with a custom HMM motif using the HMMER suite of programs, two Noggin-like/PTTH/trunk family members were identified in *Oncopeltus*. Bayesian phylogeny indicates one of these orthologs is most closely related to other insect PTTH proteins, and the other ortholog is a member of the Noggin-like family. Posterior probabilities are shown at nodes, the tree is rooted with the Noggin-like sequence identified in the *Amphimedon* genome. Multiple alignments were carried out using ClustalX [118]; the phylogeny was initially carried out under mixed models, and then with the most appropriate specific model using MrBayes. The Monte Carlo Markov Chain search was run with four chains over 1,000,000 generations with trees sampled every 1000 generations. The first 25% trees were discarded as 'burn-in'.

5.1.j Wnt pathway

Contributors: Iris M. Vargas Jentzsch and Kristen A. Panfilio

The Wnt pathway is a signal transduction pathway with fundamental regulatory roles in embryonic development in all metazoans. The emergence of several gene families of both Wnt ligands and Frizzled receptors allowed the evolution of complex combinatorial interactions with multiple layers of regulation [119]. Wnt signaling affects cell proliferation and migration, as well as segment polarity, patterning and addition in most arthropods [120]. Here we strived to identify and curate the automated models corresponding to the main components of the Wnt signaling pathway.

Methods

The protein sequences for the *wnt* ligands as well as receptors and downstream components (*armadillo*/*beta-catenin*, *dishevelled*, *frizzled*, *arrow*, *axin*, *shaggy*/*GSK-*3) from *Drosophila melanogaster*, *Tribolium castaneum* and *Acyrthosiphon pisum* were retrieved from NCBI (we excluded accessions named as 'predicted' to avoid propagating errors from automated annotations). Using these as queries, we performed tblastn searches on the *Oncopeltus* scaffolds with a cutoff maximum e-value of 1e^{-10}. Hits from all species together were ordered by scaffold and start position, and for each group of overlapping or closely adjacent hits, the putative gene name was identified by blasting back the hit sequence against Arthropoda proteins in GenBank. The query sequences with the best hits (lowest e-value) for each group were then used to identify the model to be curated, by doing a tblastn search in the *Oncopeltus* scaffolds from the blast instance at the National Agricultural Library (https://i5k.nal.usda.gov/legacy_blast). The blast results were visualized in the Apollo web browser for *Oncopeltus* (https://apollo.nal.usda.gov/oncfas/selectTrack.jsp), where the corresponding automated annotation models were edited. Homology, intron/exon boundary assessments, and protein sequence completeness were identified by manual inspection and correction of protein alignments generated with Clustal Omega (http://www.ebi.ac.uk/Tools/msa/clustalo/), and subsequent phylogenetic analyses at http://www.phylogeny.fr/ [42, 43]. For this, we used all query sequences, and included additional orthologs from the crustacean *Daphnia pulex* and the

myriapod *Glomeris marginata*. Potential gene duplications were also confirmed during this process.

The numbering for *wnt and fz* orthologs was assigned based on the corresponding vertebrate homolog (the naming of *Drosophila* orthologs was changed accordingly in phylogenetic analyses).

Results

A total of 25 models were curated for the main Wnt signaling genes on the *Oncopeltus* assembly (Table S 5.6). We identified complete gene models for six Wnt ligand subfamilies, two Frizzled transmembrane receptor families, the co-receptor *arrow*, and the downstream components *armadillo*/*beta-catenin*, *dishevelled*, *axin*, and *shaggy*/*GSK*-3. For all these genes, we were able to find the complete coding sequence, even though four genes were each split across two different scaffolds: *axin*, *dishevelled*, *wnt7* and *wntA*. The automated gene models were a good starting point for most annotated genes. However, 16 out of 23 exons had to be added *de novo* in the case of *arrow*, and we identified two possible mis-assembly instances in *wnt5* and *dishevelled*. In the case of *wnt5* there was a non-canonical splice site at the 3′ end of exon 3, and in the case of *dishevelled* exon 11 was found in between exons 6 and 7 (data supported by intron-spanning RNA-seq reads).

Two genes were duplicated, *armadillo* and *wnt8*, while all other genes were present in single copy in the assembly. Duplication and divergence of *armadillo/β-catenin*, which encodes an intracellular transducer of the Wnt pathway, was reported in the pea aphid and independently in *Tribolium* [121]. In fact, blastp searches in GenBank and phylogenetic analyses reveal that two copies are also found in a number of other hemimetabolous species, whereas most holometabolous species have a single copy. However, phylogenetic branch lengths suggest that functional divergence only occurred independently in the pea aphid and *Tribolium*. Only the bed bug and *Oncopeltus* do not conform to a strict paralogous sister gene topology, and it is unclear how many independent duplications of *armadillo* genes have occurred within the insects (Figure S 5.10). Interestingly, despite their sequence divergence, the *Tribolium* paralogs are the product of a tandem duplication, whereas the more highly conserved paralogs in *Oncopeltus* and *Cimex* are not linked in either species' assembly.

In contrast, the tandem duplication of *wnt8* in *Oncopeltus* seems to be unique, as a single *wnt8* ortholog was identified in other hemipteroid species, including the bed bug *Cimex lectularius*. The *Oncopeltus wnt8* paralogs are both expressed in the maternal/embryonic transcriptome [48], with additional expression support for *wnt8b* in the previously published adult (male) RNA-seq library ([33]; see also main text Fig. 2b).

The *Oncopeltus* Wnt ligand repertoire – *wingless/wnt1*, *wnt*5, *wnt7*, *wnt8a* and *wnt8b, wnt10* and *wntA* – is similar to the one found in the pea aphid (*Acyrthosiphon pisum*): *wingless/wnt1, wnt5, wnt*7, *wnt11, wnt16* and *wntA* [87]. In comparison, *Drosophila* and *Tribolium* have 7 and 9 Wnt subfamilies, respectively. This supports observations of a reduction in the ligand repertoire in insects compared to the 12 Wnt subfamilies inferred to have been present the last common ancestor of all arthropods [122]. Furthermore, members of the Hemiptera seem to have the fewest *Wnt* gene families reported in insects, with some of these losses perhaps having occurred relatively recently and independently in this clade. Nevertheless, assessments of gene absence need to be done with caution when dealing with draft assemblies from second generation sequencing, which is the case for most recently published genomes.

All Wnt ligand models were found isolated on different scaffolds with the exception of the *wnt8* paralogs, and *wingless* and *wnt10*. The latter two were clustered (on Scaffold 926) in the same transcriptional orientation and without other intervening genes, which is also the case in *Cimex*. This gene arrangement was also observed in *Tribolium castaneum* and *Drosophila melanogaster*, but with the *wnt6* locus between the two genes [123], reflecting the ancient arrangement of *wnt* genes in metazoans [124]. *Wnt6* was not found in the *Oncopeltus* assembly, and we also could not find evidence of *wnt6* in *Cimex lectularius*. This corroborates the postulated evolutionary loss of *wnt6* in Hemiptera [125], where the absence of *wnt6* was correlated with the absence of maxillary palps in insects.

Three models were curated for the *frizzled* (*fz*) transmembrane receptor families: two isoforms for *frizzled*, and one *frizzled-2*. These are only two of the four ancient *fz* families expected to have been present in the common ancestor of arthropods: *fz, fz2, fz3, fz4* [126]. The loss of *fz4* was also observed in *Acyrthosiphon pisum* [87].

Table S 5.6: Information on curated Wnt pathway models (in Excel Supplement).

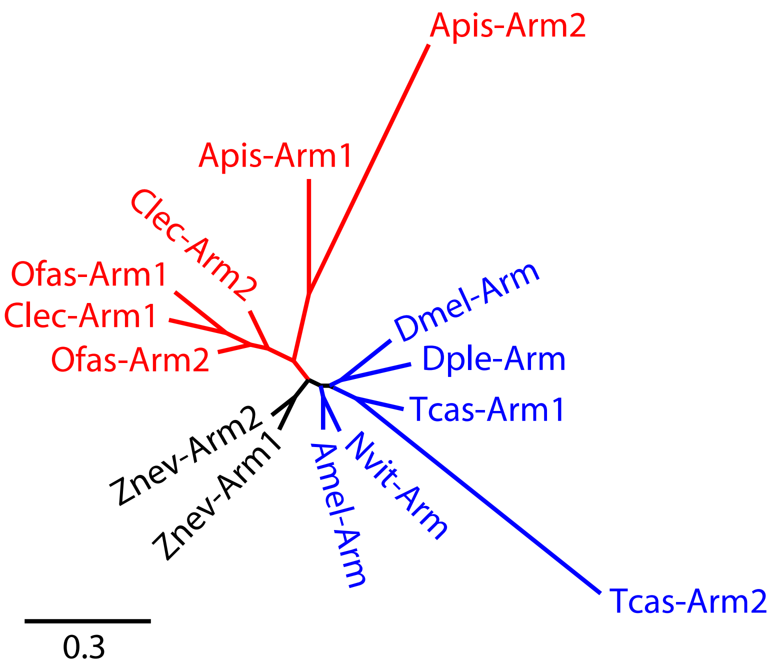

Figure S 5.10: Maximum likelihood phylogeny of Armadillo proteins, with hemipteran proteins in red and holometabolous proteins in blue. Branch length unit is substitutions per site, and all node support values are ≥62. Data are shown from nine insect species, represented by the four-letter species abbreviation and where the "Arm1" and "Arm2" paralogy distinction was arbitrarily assigned here for clarity, except in the case of *Tribolium* and the pea aphid, where public sequence accessions also bear these names. Taxonomic abbreviations and accession numbers (GenBank or *Oncopeltus* OGS) are: *Amel, Apis mellifera*, Arm: XP_006557863.1*; Apis, Acyrthosiphon pisum*, Arm1: XP_008183996.1, Arm2: XP_001946088.1*; Clec, Cimex lectularius*, Arm1: XP_014244091.1, Arm2: XP_014240454.1*; Dmel, Drosophila melanogaster*, Arm: NP_476666*; Dple, Danaus plexippus* Arm: OWR49369.1; *Nvit, Nasonia vitripennis*, Arm: XP_016840456.1; *Ofas, Oncopeltus fasciatus*, Arm1: OFAS000062, Arm2: OFAS025143*; Tcas, Tribolium castaneum*, EFA10737.1, Arm2: NP_001164124.1*;* and *Znev, Zootermopsis nevadensis*, Arm1: KDR13423.1, Arm2: KDR13424.1.

5.1.k Appendage patterning

Contributor: Yuichiro Suzuki

The transcriptional regulation of appendage patterning in insects has been well characterized in *Drosophila* [e.g. 127]. In *Drosophila*, appendages develop from imaginal discs, which are set aside during embryogenesis. Patterning of imaginal discs commences at embryogenesis and continues through larval development. During this period, the proximal-distal and the dorsal-ventral patterning of the appendages are established. During metamorphosis, the imaginal discs evert to form adult appendages [128]. Like other hemimetabolous insects, *Oncopeltus* does not form imaginal discs, and the nymphal antennae and legs resemble those of adults although the number of tarsal segments increases during the final molt. *Oncopeltus*, like other hemimetabolous insects, also differ from *Drosophila* in that they develop external wing pads during the nymphal stages that give rise to adult wings. Thus, understanding the development of *Oncopeltus* limbs has interesting evolutionary implications.

The early development of *Oncopeltus* appendage formation differs between holometabolous insects and hemimetabolous insects. In *Drosophila*, imaginal discs formation requires Wingless (Wg) [129, 130]. Similarly, *Tribolium* also requires Wg signaling for larval limb development [131]. In contrast, Wg signaling does not appear to be required for appendage patterning in *Oncopeltus* [132]. Thus, the earliest stages of appendage formation likely differ between hemimetabolous insects and holometabolous insects. Once the imaginal discs are formed, Hedgehog (Hh) signaling activates the expression of *Wg* and Decapentaplegic (*Dpp*), which interact to pattern the anterior-posterior and dorsal-ventral axes [133]. *Oncopeltus Dpp* has already been identified [132], and we have identified components of the Hedgehog pathway in the *Oncopeltus* genome. Studying these signaling pathways should provide interesting insights into the early development of appendages in hemimetabolous insects.

In the leg imaginal discs, the regional patterning in the proximal-distal axis involves gradual demarcation of the distinct regions of the limbs. At first, the imaginal disc expresses two proteins, *Distal-less* (*Dll*) in the presumptive distal portion and *homothorax* (*hth*) in the proximal portion [134-136]. Hth expression leads

to the nuclear localization of the Extradenticle (Exd) protein [137]. *Oncopeltus Dll* and *hth* have already been identified by Angelini and Kaufman [138] and appear to play similar roles in *Oncopeltus* and *Drosophila* legs. We have also identified *exd* in the genome. At the intersection of Dll and Hth, Dachshund is activated and patterns the medial portion of the developing leg in both *Drosophila* and *Oncopeltus* [138]. Thus, the regional patterning mechanisms appear to be generally conserved between *Oncopeltus* and *Drosophila.* Later on, additional sculpting of the leg occurs though segmentation, which involve several transcription factors and signaling proteins [see e.g. 127]. We have identified homologs of several of these factors (*Notch, odd-skipped, spineless, abrupt* and *bric-a-brac*) with certainty in the *Oncopeltus* genome. Whether or not these transcription factors play similar appendage patterning roles in *Oncopeltus* and *Drosophila* remains to be seen.

Many of these genes also play patterning roles in the antennae and mouthparts of holometabolous insects [e.g. 139, 140]. *Oncopeltus* have specialized mouthparts that are adapted for piercing seeds. The stylets are formed from modifications to the mandibles and the maxilla, and aspects of the genetic regulation underlying their development appear to be distinct from *Tribolium* and *Drosophila* [138]. Thus, additional comparative studies should shed light on the evolution and development of these specialized morphologies.

In *Drosophila*, the imaginal discs adopt wing/haltere identity in response to the expressions of the wing selector genes *vestigial* (*vg*) and *scalloped* (*sd*), which encode proteins that form a heterodimeric complex [141, 142]. Loss of *vg* expression leads to cell death in the wing blade [142], and loss of *sd* also leads to cell death and loss of cell proliferation in the wing pouch [143, 144]. Similarly, Apterous (*Ap*) plays a key role in wing outgrowth and development of the dorsal wing structures and the wing margins [129, 145]. We have found *vg, sd* and *ap* in the *Oncopeltus* genome. The effects of *vg* and *sd* knockdowns have recently been reported in *Oncopeltus* [146] and demonstrate similar functional roles for wing development in both *Oncopeltus* and *Drosophila*.

5.1.1 Germline genes

Contributors: Chun-che Chang, Cassandra G. Extavour, Yi-min Hsiao, Tamsin E. Jones, Hsiao-ling Lu

Germline development in the Metazoa is controlled by a relatively small number of conserved genes. We searched the *Oncopeltus fasciatus* genome for these genes (Tables S 5.7, 5.8). In contrast to the pea aphid *Acyrthosiphon pisum* – another hemipteran model with a sequenced genome – *Oncopeltus* does not have an extraordinary duplication of the most conserved germline gene families, the *vasa*, *nanos* and *piwi* families. Like most other insects, *Oncopeltus* possesses single copies of *vasa* (previously described in [147]) and *nanos*. This is in contrast to the pea aphid, which possesses four copies each of *vasa* and *nanos* [87]. This difference may reflect the simple life cycle of *Oncopeltus* as compared to the pea aphid, which utilizes both sexual and asexual life cycles and undergoes different developmental programs in these two different phases [50, 148]. We found a single *piwi* gene in *Oncopeltus*, in contrast to both *Drosophila melanogaster*, which has two (*piwi* and *aubergine*), and to *A. pisum*, which possesses eight *piwi* paralogs [149]. Similarly, in *Oncopeltus* we could only identify one *ago3* gene—another component of the piRNA machinery like *piwi*—whilst in *A. pisum* duplicated *ago3* genes (*ago3a*, *ago3b*) have been annotated (see Section 5.4.a on RNAi machinery, below). This may imply that *Oncopeltus* employs a more ancestral piRNA pathway in germ cells than both *D. melanogaster* and *A. pisum*. The *wunen* genes in *Drosophila wun* and *wun-2* are involved in germ cell development and germ cell migration. We identified a single *wunen* gene in *Oncopeltus*, which may imply that *Oncopeltus wunen* is capable alone of performing both the germ cell development and germ cell migration functions performed by *wun* and *wun-2* in *Drosophila*. We found two copies of the gene *oo18 RNA-binding protein* (*orb*), which is found in single copy in both *D. melanogaster* and *A. pisum*. *Drosophila orb* is involved in axis specification as well as germ cell development, and the functional significance of *orb* duplication in *Oncopeltus* is unknown. We did not identify any ortholog of the *oskar* gene, which has not to date been identified in any other hemipteran species and has likely been lost from this lineage of insects.

Table S 5.7: Presence/absence of targeted genes in *Oncopeltus* (additional information can be found in Table S 5.8 in the Excel supplement).

Gene name	Gene abbreviation	Number of copies found	Comments
armitage	*armi*	1	
aubergine	*aub*	0	Low expectation, specific to Diptera
belle	*bel*	1	Identified previously in [150]
boule	*bol*	1	Identified previously in [150]
bruno/arrest	*bru/aret*	1	Identified previously in [150]
cappuccino	*capu*	1	
cup	*cup*	0	Low expectation, specific to *Drosophila*
fear of intimacy	*foi*	1	
germ cell-less	*gcl*	1	
gurken	*grk*	0	Low expectation, specific to *Drosophila*
gustavus	*gus*	1	
HMG Coenzyme A reductase	*Hmgcr*	1	
homeless/spindle E	*hls/spn-E*	1	
mago nashi	*mago*	1	
nanos	*nos*	1	
oo18 RNA-binding protein	*orb*	2	
oskar	*osk*	0	Low expectation, not identified in any hemipteran species
par-1	*par-1*	1	
pipsqueak	*psq*	1	
piwi	*piwi*	1	
polar granule component	*pgc*	0	Low expectation, specific to *Drosophila*
pumilio	*pum*	1	
spire	*spir*	1	
staufen	*stau*	1	
Trapped in endoderm-1	*Tre1*	0	Expectation unclear: according to FlyBase and OrthoDB, orthologs are found in other insects and in a few other non-insect Metazoa.
tropomysin II	*Tm2*	1	
tudor	*tud*	1	Identified previously in [150]
valois	*vls*	0	Low expectation, specific to Diptera
vasa	*vas*	1	Identified previously in [150]
wunen	*wun*	1	Plays likely redundant roles in germ cell migration with *wun2* in *Drosophila*
wunen-2	*wun2*	0	

Table S 5.8: Further details of annotated germline genes (in Excel supplement).

5.1.m Eye development

Contributors: Yi-min Hsiao, Hsiao-ling Lu, Chun-che Chang

We identified twenty-one *Drosophila* homologs of eye developmental genes in the milkweed bug *Oncopeltus fasciatus* (Table S 5.9). These genes are reckoned to participate in retinal development and eye morphogenesis as their conserved roles have been identified in *Drosophila* and other insect models (Table S 5.10). In comparison with the pea aphid *Acyrthosiphon pisum*, *O. fasciatus* shares all of the 21 eye developmental genes that have been annotated in *A. pisum* except *eyegone* (*eyg*) and *Optix* [87]. Both of these genes are unlikely to be missing in the *Oncopeltus* genome because their homologs can be identified in other insect models. Nonetheless, *twin of eyegone* (*toe*)—a tandem duplicated sister paralog of *eyg* in *Drosophila* and other higher dipterans [151] —was not found in both *A. pisum* and *Oncopeltus*. In *A. pisum* a homolog of the *eyeless* (*ey*) gene was not identified, and in *Oncopeltus* we could only find an incomplete contig without sequences encoding the homeodomain of the EY protein. This suggests that *Oncopeltus* possess a pseudogene of *ey* homologs or part of its complete sequence is missing in the database.

Table S 5.9: Major components of eye development in *Oncopeltus fasciatus.*

Gene name	Gene abbreviation	Number of copies found	Comments
twin of eyeless	*toy*	1	
eyeless	*ey*	1	Incomplete sequences, loss of HD domain
eyes absent	*eya*	1	
twin of eygone	*toe*	0	Not identified in *Oncopeltus*, specific to *Drosophila*
eyegone	*eyg*	0	Not identified in *Oncopeltus*
sine oculis	*so*	1	
Optix	*Optix*	0	Not identified in *Oncopeltus*
lozenge	*lz*	1	
hedgehog	*hh*	1	
sevenless	*sev*	1	
bride of sevenless	*boss*	1	
Son of sevenless	*Sos*	1	CDS split in three different scaffolds
downstream of receptor kinase	*drk*	1	
dachshund	*dac*	1	

atonal	*ato*	1	
decapentaplegic	*dpp*	1	
Transforming growth factor alpha	*TGFα*	1	
EGF receptor	*Egfr*	1	
delta	*Dl*	1	
serrate	*ser*	1	
Notch	*N*	1	

Table S 5.10: Orthologous numbers of eye developmental genes in four insect species.

Gene	Fruit fly	Beetle	Pea aphid	Milkweed bug
toy	1	1	1	1
ey	1	1	NA	NC
eya	1	1	1	1
toe	1	NA	NA	NA
eyg	1	1	1	?
so	1	1	1	1
Optix	1	1	1	?
lz	1	1	1	1
hh	1	1	1	1
sev	1	?	1	1
boss	1	?	1	1
Sos	1	1	2	1
drk	1	?	1	1
Wg	1	1	1	1
dpp	1	1	4	1
dac	1	1	?	1
ato	1	1	1	1
Egfr	1	1	1	1
N	1	1	1	1

?: genes that have not been identified

NA: sequences are not available.

NC: sequences are incomplete.

5.2 Structural and differentiation genes

5.2.a Bristle and neural development

Contributors: David Armisén, Antonin J.J. Johan Crumière, Séverine Viala, Essia Sghaier, Chloe S. Berger, Maria Emilia Santos, Peter N. Refki, Abderrahman Khila.

Abstract

The term “bristle” includes various hair-like structures with different functions, including mechanosensory and chemosensory hairs that are in constant contact with substrates and air, allowing the insect to sense its surrounding environment. For this reason, many genes involved in bristle development have been previously described as playing a role in neural development in *Drosophila* [152, 153]. In this study we annotated and analyzed 88 genes known to be involved in bristle development. Our results show an overall high conservation of protein sequence compared with *Drosophila* despite a higher intron number.

Results and discussion

The most studied role of insect bristles is perhaps their function as sensory organs for detecting various environmental stimuli. During development, each bristle is built from a small cluster of specialized cells including sensory neurons and support cells [154]. The shaft of the bristle extends from a single cell primarily via cytoskeletal arrangements [155]. In the fly *Drosophila*, the development of these bristles is quite well described and is regulated by a set of conserved developmental genes [156]. QTL studies have uncovered dozens of candidate genes and regions linked to variation in bristle density and morphology [152]. Some of this variation is also attributable to changes in non-coding sequences of a number of conserved developmental genes such as the achaete-scute complex [157, 158]. Based on fly genetics, we established and annotated a list of 105 genes known to be involved in neurogenesis and bristle development, as well as in variation in bristle number and density ([152, 153]; Table S 5.11). Among these, we were able to annotate 88 genes in the *Oncopeltus* genome, with no duplications, while we could not identify homologs for the remaining 17 candidate genes. Sequence comparison of the 88 genes with their homologous counterparts in *Tribolium castaneum*, *Acyrthosiphon pisum*, *Rhodnius prolixus*,

Pediculus humanus, *Anopheles gambiae* and *Drosophila melanogaster* revealed a median of ~46 % protein identity. *Drosophila* alignment in particular shows that our dataset contains many genes encoding proteins recovered with full length, such is the case of the highly conserved gene Actin-5C [159]. Alignments show that many *Oncopeltus* genes with shorter sequence than *Drosophila* homologs have missing sequence primarily in the N-terminal and C-terminal. The number of exons of *Oncopeltus* compared with their *Drosophila* homologs suggests that in many cases this reduced size is due to missing exons. As neither sequence similarity nor RNA-seq data available support the presence of additional exons, a possible explanation for this absence could be a technical artifact due to problems in the assembly process or the impossibility to close all the gaps in the genome.

The lack of some exons highly contrasts with the higher number of exons included in *Oncopeltus* genes in average compared with *Drosophila*. It is striking that some particular genes such as *flightless* have a higher number of exons (nineteen in *Oncopeltus* instead of four in *Drosophila*) (Table S 5.11, Figure S 5.11), despite remaining shorter than its *Drosophila* homolog. In contrast, other genes, such as *raspberry* and *Ras85D*, have lost their introns and are rather encoded by single exons, (Figure S 5.12 and Figure S 5.13). This indicates that despite a substantial divergence in gene structure between *Oncopeltus* and *Drosophila*, there is high conservation in the encoded protein, suggesting possible strong selection on the coding sequence of these genes.

On the other hand, we can divide the 17 missing genes into four categories depending on the potential meaning of our failure to identify them in *Oncopeltus* genome. The first category includes genes that might be inherently hard to identify because their coding sequence is too short, such is the case of *Bantam*, a miRNA that is only 81 nucleotides long. The second category includes genes not found outside the *Drosophila* genus or closely related species through a 'nr' blastp search, such as *corto*, *dalmatian*, *diminutive*, *gliolectin* and *mushroom body defect*, and we therefore believe that they are truly missing from the *Oncopeltus* genome. The third category includes genes that we would have expected to find in the *Oncopeltus* genome, such as *Rala*, *effete*, *bonus* and *nuclear fallout*, as these genes have been described in *Tribolium castaneum* and *Acyrthosiphon pisum* or are highly and widely conserved in many species. The lack of *Oncopeltus* homologs is therefore very likely to be caused by an assembly artifact and not by a real gene loss. Lastly, in the fourth category, genes

such as *bifocal*, *buttonless*, *cabut*, *escargot*, *pxb*, *spitz* and *Twin of m4* similar genes are present in other close species like *Tribolium castaneum* or *Acyrthosiphon pisum* but have diverged more and their absence in *Oncopeltus* genome can be either real, or caused by a high divergence that prevents blast recognition, or can be the result of assembly problems. Therefore, in this last instance we do not have enough elements to discern the cause of absence for each gene.

Methods

We annotated the bristle development genes by performing BLAT and tblastn searches on the *Oncopeltus* scaffolds with the corresponding *Drosophila* gene protein sequences available in FlyBase (release 5 for first round annotation and release 6 for annotation verification). To confirm orthology, we then blasted our *Oncopeltus* models into NCBI. Homology, intron/exon boundary assessments, and protein sequence completeness were identified by manual inspection using RNA-seq alignments available and protein alignments generated with Clustal Omega (http://www.ebi.ac.uk/Tools/msa/clustalo/).

Table S 5.11: Annotation of bristle and neural development genes: number of exons of the longest isoform. Percentage of identity calculated using protein alignment made by Clustal Omega (in Excel Supplement). Includes also a list of genes not found or only partially annotated.

```
CLUSTAL O(1.2.1) multiple sequence alignment


Flightless_FBpp0076893      ---MSVLPFVRGVDFTKNDFSA-TFPSSMRQMSRVQWLTLDRTQLAEIPEELGHLQKLEH
FlightlessI_Oncopeltus      MANTGVLPFVRGVDFSRNDFSDGKFPSSVRLMTGLQWLKLDRTHLDNIPEELGNLMKLEN
                                .**********::****  .****:* *: :***.****:* :******.* ***.

Flightless_FBpp0076893      LSLNHNRLEKIFGELTELSCLRSLDLRHNQLKNSGIPPELFHLEELTTLDLSHNKLKEVP
FlightlessI_Oncopeltus      LSLVRNDLERLHGELTELPCLRSLIIRHNKVKSSGIPADLFRSEELTTLDLSHNALKEVP
                            *** :* **::.****** ***** :***::*.**** :**: *********** *****

Flightless_FBpp0076893      EGLERAKNLIVLNLSNNQIESIPTPLFIHLTDLLFLDLSHNRLETLPPQTRRLINLKTLD
FlightlessI_Oncopeltus      DGLEKCKTVLVLNLSHNNITSIPNTLFMNLTDLLFLDLSNNLLETLPPQMRRLGNLQTLI
                            :***:.*.::*****.*:* ***. **:.**********.* ******* *** **:**

Flightless_FBpp0076893      LSHNPLELFQLRQLPSLQSLEVLKMSGTQRTLLNFPTSIDSLANLCELDLSHNSLPKLPD
FlightlessI_Oncopeltus      LNNNPLAHFQLRQLPSLVSLESLHMRYTQRTLSNLPSSLEMLTNLTDVDLSYNSLPKIPD
                            *..***  ********* *** *:*  ***** *:*:*:: *:** ::***:*****:**

Flightless_FBpp0076893      CVYNVVTLVRLNLSDNELTELTAGVELWQRLESLNLSRNQLVALPAALCKLPKLRRLLVN
FlightlessI_Oncopeltus      CLFTLVNLKRLNLSNNYLSELSLALEVWQRLETLNLSSNKLTSLPASVCKLTALRRLYLN
                            *::.:*.* *****:* *:**: .:*:*****:**** *:*.:***::***  **** :*

Flightless_FBpp0076893      DNKLNFEGIPSGIGKLGALEVFSAANNLLEMVPEGLCRCGALKQLNLSCNRLITLPDAIH
FlightlessI_Oncopeltus      DNQLDFEGIPSGIGKLSNLEVFSAASNQLEMIPEGLCRCGSLKKLILSSNRLITLPDTVH
                            **:*:***********. *******.* ***:********:**:* **.********::*

Flightless_FBpp0076893      LLEGLDQLDLRNNPELVMPPKPSEASKATSLEFYNIDFSLQTQLRLAGAAVPPSMPSSAT
FlightlessI_Oncopeltus      LLSDLQVLEVKDNPDLVMPPRPCEAQRGSGLEYYNIDFSLQTQLRLAGAALPQPLPQTSA
                            **. *: *::::**:*****:*.**.:.:.**:*****************:*  :*.:::

Flightless_FBpp0076893      PKDSTARKIRLRRGPRS--EGDQDAAKVLKGMKDVAKDKDNEAGAVPEDGKPESLKPKRW
FlightlessI_Oncopeltus      SKDPIARKLRLRRSRRDAEEADSDQAKILKGMKDIATEKNKC--KTEEEERAESLKPKRW
                             **  ***:****. *.  *.*.* **:******:*.:*::    . *: : ********

Flightless_FBpp0076893      DESLEKPQLDYSKFFEKDDGQLPGLTIWEIENFLPNKIEEVVHGKFYEGDCYIVLKTKFD
FlightlessI_Oncopeltus      DESLEKPPLDYSELFDEDAGQIPGITVWEIENFLPNMVEEAVHGKFFQADCYIILKTSLD
                            ******* ****::*::* **:**:*:********* :**.*****::.****:***.:*

Flightless_FBpp0076893      DLGLLDWEIFFWIGNEATLDKRACAAIHAVNLRNFLGARCRTVREEQGDESEQFLSLFET
FlightlessI_Oncopeltus      DSGNLAWNIFFWIGDKATLDKRACAAIHAVNLRNYLGAECRTAREEQGEESEEFLALFPS
                            * * * *:******::******************:***.***.*****:***:**:** :

Flightless_FBpp0076893      EVIYIEGGRTATGFYTIEEMIHITRLYLVHAYGATIHLEPVAPAITSLDPRHAFVLDLGT
FlightlessI_Oncopeltus      GITYIQGGRTPSGFYTVEDMTYITRLYRVHGAGAGIHLEPVPISYESLDPRYVFILDTGL
                             : **:**** :****:*:* :***** **. ** ******  :  *****:.*:** *
```

```
Flightless_FBpp0076893      HIYIWMGERSKNTLNSKARLMAEKISKTERKNKCEIQLERQGEESAEFWQGLGMTSEEAD
FlightlessI_Oncopeltus      KIFMWYGKKSKNTFRSKARLLCEKINKNERKGKAEIMTELYKEESEDFWCSLGEESGK-P
                            :*::* *::****:.*****:.***.*.*** *.**  *   *** :** .**  * :

Flightless_FBpp0076893      AAEPPKEHVPEDYQPVQPRLYQVQLGMGYLELPQVELPEQKLCHTLLNSKHVYILDCYTD
FlightlessI_Oncopeltus      PEEPVIEHVRNEWHPVEPRLYQVKLGMGYLELPQVELPCNRLEHTLLNSRNVYILDCYLD
                              **  *** ::::**:******:************** ::* ******:.******* *

Flightless_FBpp0076893      LFVWFGKKSTRLVRAAAVKLSRELFNMMDRPDYALVMRVPEGNEMQIFRTKFAGWDEVMA
FlightlessI_Oncopeltus      VFVC--------------------------------------------------------
                            :**

Flightless_FBpp0076893      VDFTRTAKSVAKTGANLTQWARQQETRTDLAALFMPRQSAMPLAEAEQLEEEWNYDLEMM
FlightlessI_Oncopeltus      ------------------------------------------------------------


Flightless_FBpp0076893      EAFVLENKKFVRLPEEELGRFYTGECYVFLCRYCIPIEEPENGSEDGANPAADVSKSSAN
FlightlessI_Oncopeltus      ------------------------------------------------------------


Flightless_FBpp0076893      NQPEDEIQCVVYFWQGRNAGNMGWLTFTFTLQKKFKAMFGEELEVVRIFQQQENLKFMSH
FlightlessI_Oncopeltus      ------------------------------LQKKFKSLFGEKLEVVRTHQQQENIKFLAH
                                                          ******::***:*****  .*****:**::*

Flightless_FBpp0076893      FKRKFIIHTGKRKDKAHTAKGKSPVEFFHLRSNGGALTTRLIQINPDAVHLNSTFCYILH
FlightlessI_Oncopeltus      FKRKLVIHTGKRNTPR-DPNQPPPVEFYHLRANGGPLCTRLVQIKPDASTLNSAFCYILK
                            ****::******:      :   ****:***:*** * ***:**:***  ***:*****:

Flightless_FBpp0076893      VPFETEDDSQSGIVYVWIGSKACNEEAKLVQDIAEQMFNSPWVSLQILNEGDEPENFFWV
FlightlessI_Oncopeltus      VPFDSEE--SSGIVYVWIGSKANTDDIRLIGEIADEMFNNPWISLQVVNEGEEPDNFFWV
                            ***::*:  .************ .:: :*: :**::***.**:***::***:**:*****

Flightless_FBpp0076893      ALGGRKPYDTDAEYMNYTRLFRCSNERGYYTVAEKCADFCQDDLADDDIMILDNGEHVFL
FlightlessI_Oncopeltus      ALGGRKPYDKDADFMAYTRLFRCSNEKGYFIVSEKCSDFCQEDLADDDIMILDNGEQVFL
                            *********.**::* **********:**: *:***:****:**************:***

Flightless_FBpp0076893      WMGPRCSEVEVKLAYKSAQVYIQHMRIKQPERPRKLFLTMKNKESRRFTKCFHGWSAFKV
FlightlessI_Oncopeltus      WLGAKCSEVEVKLAYKSAQVCIF-------------------------------------
                            *:* :*************** *

Flightless_FBpp0076893      YL
FlightlessI_Oncopeltus      --
```

Figure S 5.11: Protein alignment made by Clustal Omega. Intron boundaries highlighted in blue for *Drosophila* and yellow for *Oncopeltus*.

```
CLUSTAL O(1.2.1) multiple sequence alignment


raspberry_FBpp0071423       MESTTKVKVNGFVESTSSSAAPAIQTKSTTGFDAELQDGLSCKELFQNGEGLTYNDFLIL
raspberry_Oncopeltus        ------------------------------MTDDIPVDGLSGKELFSNGDGLTYNDFIIL
                                                          *    **** ****.**:*******:**

raspberry_FBpp0071423       PGYIDFTAEEVDLSSPLTKSLTLRAPLVSSPMDTVTESEMAIAMALCGGIGIIHHNCTPE
raspberry_Oncopeltus        PGFIDFTPDEVDLRSELTKKITLQSPLVSSPMDTVTESDMAIAMALCGGIGIIHHNCTAE
                            **:**** :**** * ***.:**::*************:******************* *

raspberry_FBpp0071423       YQALEVHKVKKYKHGFMRDPSVMSPTNTVGDVLEARRKNGFTGYPVTENGKLGGKLLGMV
raspberry_Oncopeltus        YQANEVHKVKKYKHGFIRHPVVLSPKNTVADVFQVKKEHGFCGIPITENGQLGGKLCGIV
                            *** ************:*.* *:**.***.**::.:::.** * *:****:***** *:*

raspberry_FBpp0071423       TSRDIDFRENQP--EVLLADIMT--TELVTAPNGINLPTANAILEKSKKGKLPIVNQAGE
raspberry_Oncopeltus        TSRDIDFLADPNTHTILLEKVMTKLENIISAKAGVTLEEANHLLEESKKGKLPIINEKGE
                            *******  :     :** .:**   ::::*  *:.*  ** :**:********:*: **

raspberry_FBpp0071423       LVAMIARTDLKKARSYPNASKDSNKQLLVGAAIGTRSEDKARLALLVANGVDVIILDSSQ
raspberry_Oncopeltus        LVALIARTDLKKNRDYPKASKDENKQLLVGAAIGTRPEDQERLKLLASAGADVVVLDSSQ
                            ***:******** *.**:****.************* **: ** **.: *.**::*****

raspberry_FBpp0071423       GNSVYQVEMIKYIKETYPELQVIGGNVVTRAQAKNLIDAGVDGLRVGMGSGSICITQEVM
raspberry_Oncopeltus        GNSIFQVKMIKYIKETYPNLQVIGGNVVTAAQAKNLIDAGVDGLRVGMGSGSICITQEVM
                            ***::**:**********:********** ******************************

raspberry_FBpp0071423       ACGCPQATAVYQVSTYARQFGVPVIADGGIQSIGHIVKAIALGASAVMMGSLLAGTSEAP
raspberry_Oncopeltus        AVGRPQGTAVYKVAEYARRFGVPIIADGGIQSIGHVTKALSLGASTVMMGSLLAGTSEAP
                            * * **.****:*: ***:****:***********:.**::****:**************

raspberry_FBpp0071423       GEYFFSDGVRLKKYRGMGSLEAMERGDAKGAAMSRYYHNEMDKMKVAQGVSGSIVDKGSV
raspberry_Oncopeltus        GEYFFSDGVRLKKYRGMGSLEAMNRKDAQGSAMDRYFHSEMDKVKVAQGVSGSIVDKGSV
                            ***********************:* **:*:**.**:*.****:****************

raspberry_FBpp0071423       LRYLPYLECGLQHSCQDIGANSINKLRDMIYNGQLRFMKRTHSAQLEGNVHGLFSYEKRLF
raspberry_Oncopeltus        LRFLPYIQCGIQHGCQDIGARSLYVLRDMMYEGNLRFERRTHSAQNEGNVHSLFSYEKRLF
                            **:***::**:**.******.*:  ****:*:*:*** :****** *****.*********
```

Figure S 5.12: Protein alignment made by Clustal Omega. Intron boundaries in *Drosophila* highlighted in blue.

```
CLUSTAL O(1.2.1) multiple sequence alignment


Ras85D_FBpp0081600          MTEYKLVVVGAGGVGKSALTIQLIQNHFVDEYDPTIEDSYRKQVVIDGETCLLDILDTAG
Ras85D_Oncopeltus           MTEYKLVVVGAGGVGKSALTIQLIQNHFVDEYDPTIEDSYRKQVVIDGETCLLDILDTAG
                            ************************************************************

Ras85D_FBpp0081600          QEEYSAMRDQYMRTGEGFLLVFAVNSAKSFEDIGTYREQIKRVKDAEEVPMVLVGNKCDL
Ras85D_Oncopeltus           QEEYSAMRDQYMRTGEGFLLVFAVNSIKSFEDIGMYREQIKRVKDAEEVPMVLVGNKCDL
                            ************************** ******* *************************

Ras85D_FBpp0081600          ASWNVNNEQAREVAKQYGIPYIETSAKTRMGVDDAFYTLVREIRKDKDNKGRRGRKMNKP
Ras85D_Oncopeltus           HSWAVDMNQAREIAKNYSIPFVETSAKTRMGVDEAFYTLVREIRKDKEVRGKEKRKGINK
                             ** *: :****:**:*.**::***********:*************: :*:. **  :

Ras85D_FBpp0081600          NRRFKCKML
Ras85D_Oncopeltus           NRRKRCWIL
                            *** :* :*
```

Figure S 5.13: Protein alignment made by Clustal Omega. Intron boundaries in *Drosophila* highlighted in blue.

5.2.b Molting and metamorphosis genes

Contributors: Deniz Erezyilmaz and Yuichiro Suzuki

Although the milkweed bug has been a key research model for hemimetabolous endocrine studies since the 1960's [160-164], only a handful of genes that regulate the progression through postembryonic development have been cloned from *Oncopeltus* since that time [53, 165]. We therefore searched for genes that are involved in regulation of the molt cycle, cuticle identity, or ecdysis (see also manuscript main text).

For the cytochrome P450 enzymes of the Halloween family, which synthesize the ecdysteroid hormones that trigger the molting cycle, we have found all of the key P450 genes involved in ecdysone biosynthesis, including *spook*, *phantom*, *disembodied* and *shadow* [166]. We also identified *shade*, which encodes a P450 enzyme that converts ecdysone to its active form, 20-hydroxyecdysone, at target tissues [167].

Pioneering studies in *Drosophila* of the transcriptional response to ecdysone produced a model whereby a small set of 'early genes' are transcribed, and their gene products: (1) activate a large number of 'late genes', and (2) suppress their own transcription. Subsequent cloning of early and late genes involved in the ecdysone response cascade revealed a battery of transcription factors [168] many of which belong to the nuclear receptor superfamily (see also Supplemental Note 5.1.e). Early genes with clear *Oncopeltus* orthologs include *E74*, the previously characterized *E75* [53], *ultraspiracle* (*usp*), a homolog of the mammalian Retinoid-X-receptor (RXR), and its heterodimeric binding partner, the ecdysone receptor (EcR). Another early gene with stage-specific expression, the nuclear receptor gene *E93*, is also present in the *Oncopeltus* genome. This gene was recently shown to be required for adult metamorphosis in other hemi- and holometabolous insects [169]. Among the 'delayed early genes', which are known targets of EcR/USP heterodimers in *Drosophila* [84, 168, 170, 171], we additionally identified the nuclear receptors *E78, HR3, HR4, βftz-f1, HR39*. In *Drosophila*, the hormone receptor HR4 [172] represses early genes, but activates expression of *βftz-f1*, a midprepupal gene. βFtz-F1, in turn, is required for optimal expression of early genes at the next molt in flies [173]. Finally, we discovered an ortholog encoding HR38, an orphan receptor that has been shown to

heterodimerize with USP to mediate ecdysone signaling in *Drosophila* without directly binding ecdysteroids [174].

Ecdysis is driven by eclosion hormone (EH; [175]), ecdysis triggering hormone (ETH; [176]), crustacean cardioactive peptide (CCAP; [177]), and bursicon [178]. We have found genes encoding orthologs of EH, ETH, and the ETH receptor in the *Oncopeltus* genome. We were also able to identify a portion of the *ccap* ortholog in the *Oncopeltus* genome, and its putative ortholog encoding its receptor was represented in transcriptomic data.

5.2.c Structural cuticular proteins and pigmentation

Contributors: Andrew J. Rosendale, Joshua B. Benoit, Yuichiro Suzuki

Changes in the expression of specific cuticle proteins have been associated with increased stress tolerance and insecticide resistance [179, 180]. We identified 173 putative cuticle proteins using sequence motifs established by Willis [179, 181, 182] from the milkweed bug genome (Table S 5.12). Similar to other insects, the CPR family, with the RR-1 (soft cuticle), RR-2 (hard cuticle), and unclassifiable types, constituted the largest group of cuticle protein gene groups for *Oncopeltus*. The number of genes in the protein families of CPAP1, CPAP3, and TWDL were similar to the number in other insects [179, 182], but with a slight expansion in the CPF family compared to other taxa (Figure S 5.14).

Table S 5.12: Number of genes identified as putative cuticle proteins per family in the *Oncopeltus* genome.

CPR[1]								
RR-1	RR-2	Uncl	CPAP1	CPAP3	CPF	TWDL	Unclassified	Total
29	75	29	11	7	7	3	12	173

[1] Sequences that scored above the assigned cutoffs for the RR-1 and RR-2 models were classified as the corresponding type, whereas sequences with scores below the assigned cutoffs but above 0 were characterized as "unclassified" (for more information, see [181]).

Melanization of the cuticle has been suggested as critical in the prevention of excessive water loss [183]. Furthermore, understanding the physiological and molecular regulation of pigmentation synthesis has important implications for understanding the evolution of aposematic (warning) coloration, and we therefore analyzed components of the pathways responsible for the main color elements. We identified key genes associated with melanization and red pigment production for *Oncopeltus*. These include genes encoding Tyrosine hydrolase [184], Yellow [185], Dopa decarboxylase [184], Ebony [186], and at least two Phenol oxidases (Figure S 5.15). RNAi studies of these genes show that these genes play critical roles in melanin synthesis [187, 188].

In addition, the *Oncopeltus* abdomen and eyes also produce ommochrome pigments [189]. The regulation of ommochrome biosynthesis has been well characterized in *Drosophila* eye development. The key genes involved in this process (*vermilion*, *arylformamidase* or *kynurenine formamidase*, *cinnabar*, *white* and *scarlet*) were identified in the *Oncopeltus* genome. Thus, most, if not all, of the key enzymes involved in ommochrome synthesis appear to be present in the *Oncopeltus* genome.

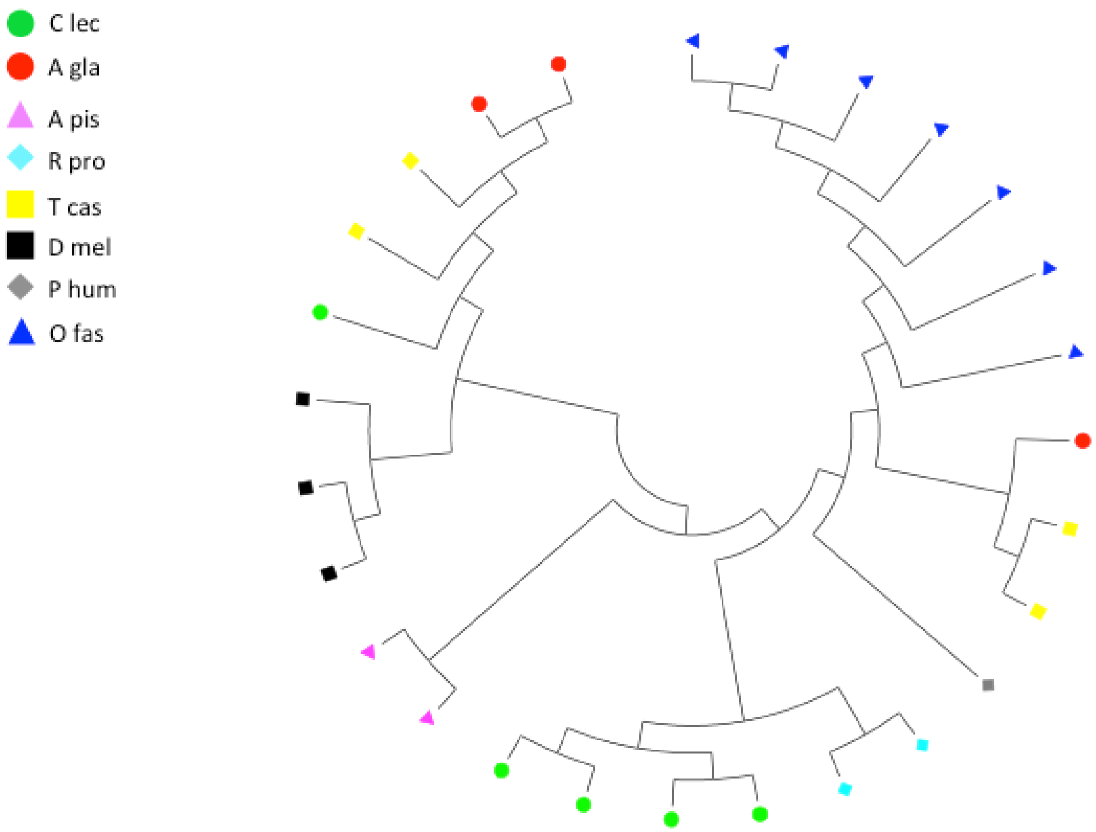

Figure S 5.14: Phylogenetic tree demonstrating relationship of CPF proteins from, *Cimex lectularius* (Clec), *Anoplophora glabripennis* (Agla), *Acyrthosiphon pisum* (Apis), *Rhodnius prolixus* (R pro), *Tribolium castaneum* (Tcas), *Drosophila melanogaster* (Dmel), *Pediculus humanus* (P hum), and *Oncopeltus fasciatus* (Ofas). The tree was constructed using the neighbor-joining method in MEGA6; Poisson correction and bootstrap replicates (2,000 replicates) were used.

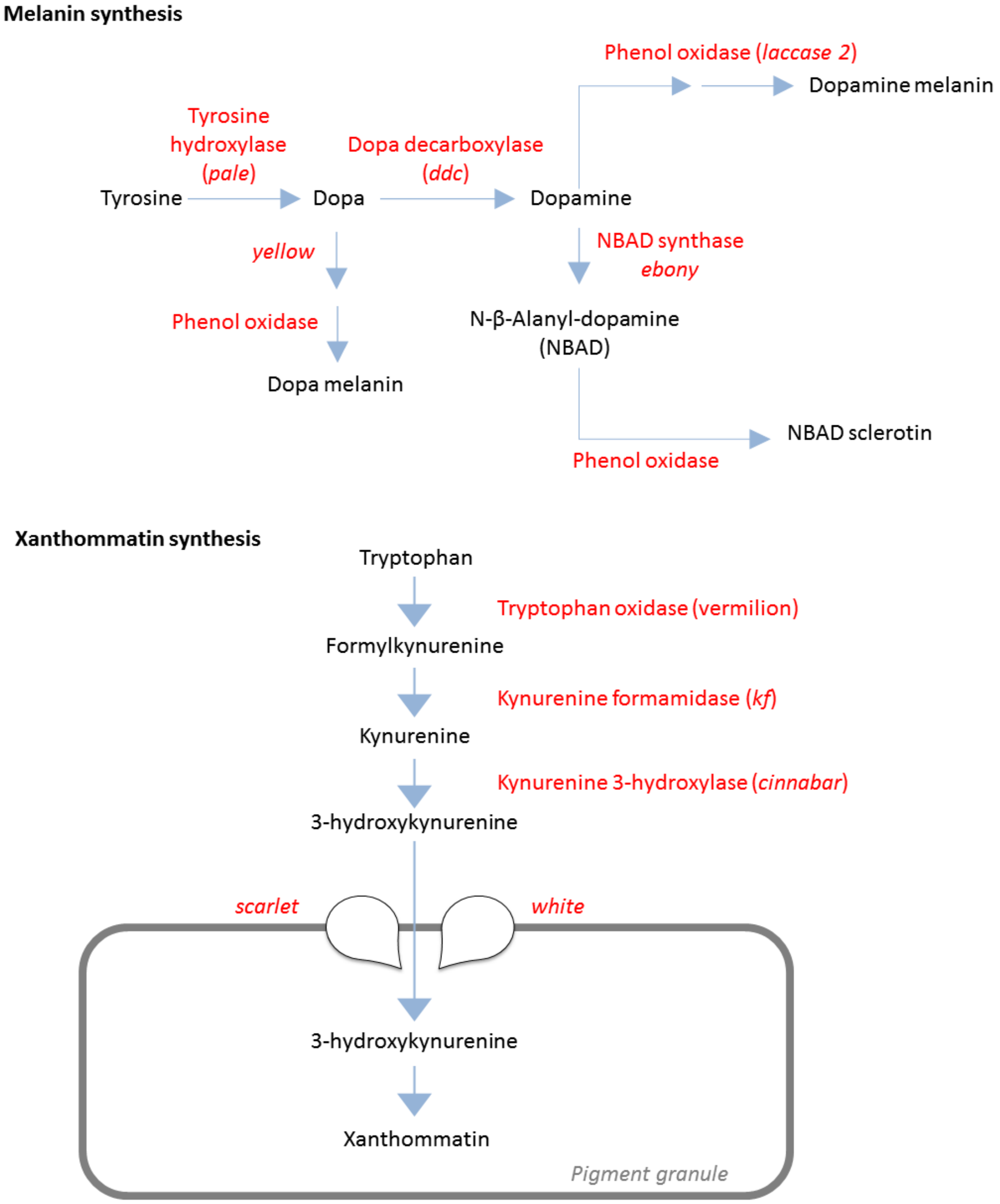

Figure S 5.15: The *Oncopeltus* genome contains most of the genes encoding enzymes involved in melanin and ommochrome synthesis. In red are all the enzymes that are encoded by genes identified in the current *Oncopeltus* assembly.

5.3 Environmental adaptations

5.3.a Stress response

Contributors: Elise M. Didion, Emily C. Jennings, Joshua B. Benoit

Antioxidant genes

Insects possess a suite of antioxidant proteins that prevent oxidative damage associated with various physiological processes. In *Oncopeltus*, 22 genes associated with the tolerance of oxidative stress were identified. Compared to other insects, the antioxidant system of *Oncopeltus* was well conserved; however, the number of catalase paralogs was greater in *Oncopeltus* than in insects such as *Drosophila* and *Tribolium* (Table S 5.13). In *Oncopeltus* three superoxide dismutases (including Cu/Zn and Mn/Fe SOD) catalyze superoxide to H_2O_2 and four catalases are present to convert H_2O_2 to water and oxygen. A total of nine genes are involved in the reduction of H_2O_2, including five peroxidoxins and four thioredoxin peroxidases. The thioredoxin system, responsible for maintaining proteins in a reduced state and scavenging reactive oxygen species, includes two genes for thioredoxin reductase. One dihydrolipoamide dehydrogenase scavenges nitric oxide and one gene for glutathione peroxidase catalyzes the breakdown of H_2O_2 and hydroperoxides. Both dual oxidase and nitric oxide synthase (one gene each) are involved in immune response. In *Oncopeltus*, antioxidants not only play an important role in normal metabolic processes, but also in the survival of oxidative stress from xenobiotic factors [190].

Aquaporin genes

Aquaporins (AQPs) impact organismal stress tolerance, specifically under periods of dehydration and cold stress, by the regulation of cellular water levels [191]. We have identified seven aquaporin genes for the milkweed bug that include those that encode for a Drosophila-integral protein (Drip), AQP2, AQP4 (two sequences), AQP5, AQP6 and Bib (Table S 5.15, Figure S 5.16). This number falls within the range of most insects (6-8) and *Oncopeltus* has members of each group previously identified from insects [191]. The number of genes is identical to those recovered from the bed bug, *Cimex lectularius* [191].

Heat shock protein genes

Heat shock proteins (Hsps) have been documented as key in relation to stress resistance under a multitude of conditions [192]. In general, there were no major expansions or retractions associated with Hsps for *Oncopeltus* when compared to other insects (Table S 5.15).

Table S 5.13: *Oncopeltus* antioxidant-associated genes (in Excel supplement).

Table S 5.14: *Oncopeltus* aquaporin-associated genes (in Excel supplement).

Table S 5.15: *Oncopeltus* heat shock proteins (in Excel supplement).

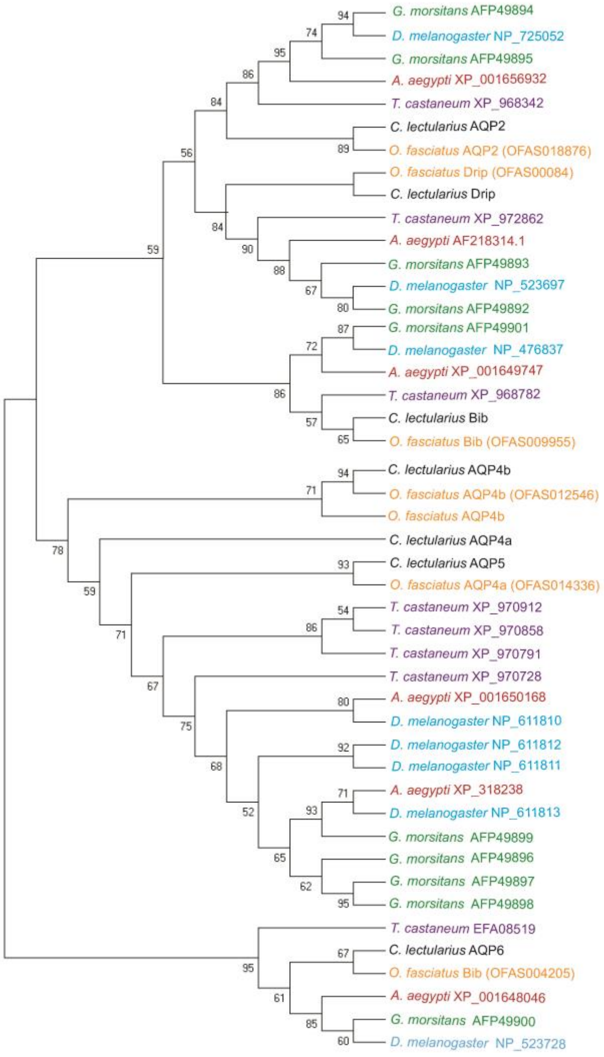

Figure S 5.16: Comparison of predicted aquaporins from other insects. Neighbor-joining tree was produced using MEGA6 using Dayhoff model and pairwise matching; branch values indicate support following 1500 bootstraps; values below 50% are omitted.

5.3.b Cytochrome P450s

Contributors: Iris M. Vargas Jentzsch, Yuichiro Suzuki, Kristen A. Panfilio

Cytochrome P450 genes encode one of the largest and most diverse enzyme families, and they can be found in the genomes of all domains of life [193]. Among an ample range of functions, P450 enzymes play a central role in the metabolism of xenobiotics and the production of hormones (see also Section 5.2.b, above). Despite their high sequence variability and substrate specificity, the overall structure of cytochrome P450 genes is highly conserved [193, 194].

As a starting point for our P450 curation, we took a sample of ten curated P450s in the bed bug *Cimex lectularius* [19], which represented all major clades in a phylogenetic tree these proteins. The selected gene models were used as queries for blastn searches into the *O. fasciatus* scaffolds, and we annotated 17 gene models from the corresponding matches (Table S 5.16). These genes, as well as additional models, were also curated as part of the gene set involved in molting and metamorphosis (see main text and Section 5.2.b, above), adding up to 20 P450 gene models. To predict which of the remaining automated models could correspond to a P450 family, we used the orthology analysis results from the OrthoDB analysis (Section 6.1). We identified all Insecta orthologous groups (OGs) containing one or more bed bug P450 gene model, and checked if these also contained *O. fasciatus* gene models (OFAS IDs). Of the bed bug P450 proteins, 69% (40 of 58) were assigned to an OG, spanning 18 distinct OGs. These 18 OGs also contained a total of 53 *O. fasciatus* proteins, including 82% (14 of 17) of our *O. fasciatus* curated P450 models (see details in Table S 5.16). Overall, between manual curation and protein orthology, we have identified a total of 58 potential P450 gene models in the *O. fasciatus* assembly.

Table S 5.16: Details on the manually curated P450 proteins in *Oncopeltus fasciatus*, and the list of OFAS IDs belonging to P450 orthologous groups identified by homology with P450s identified in *Cimex lectularius* (in Excel supplement).

5.3.c Insecticide resistance

Contributors: Lucila Traverso and Rolando Rivera Pomar

In order to provide information about the presence of genes involved in the response to insecticides in the genome of *O. fasciatus*, useful for studies on insecticide resistance, it was performed a similarity search that resulted in the annotation of 19 genes. Referring to the Glutathione S Transferase (GST) family, a microsomal GST, and one member of each class of the family (Sigma, Zeta, Omega, Delta, Theta) were annotated. Within the Carboxylesterase (COE) family, the Glutactin and Neuroligin genes were annotated, as also the sites of action of insecticides acetylcholinesterase 1 and acetylcholinesterase 2. Other genes corresponding to sites of action of insecticides that were annotated are the voltage-gated sodium channel and the GABA Receptor. In addition, it was performed the annotation of genes involved in the response to oxidative stress, also important in the response to insecticides, such as Catalase, Superoxide Dismutase 1, Superoxide Dismutase 2, Heme Peroxidase, Glutathione Peroxidase and Thiorredoxin Reductase. Another gene related to xenobiotic detoxification is UDP-glucuronosyltransferase.

5.3.d Neuropeptides and their receptors

Contributors: Lucila Traverso and Rolando Rivera Pomar

Neuropeptides are cell-to-cell signaling molecules that act as hormones, neurotransmitters, and/or neuromodulators of feeding, behavior or basic physiological processes. By homology search, we found 31 genes encoding at least 52 splicing variants of neuropeptides (see Table S 5.17). Among others, *sulfakinin* precursor genes were not found, as in *A. pisum*, but present in *R. prolixus*. We identified 32 genes encoding neuropeptide and protein hormone GPCRs. Interesting features were found in *O. fasciatus* neuropeptidome, comparing to other insects; some of the neuropeptides, Kinins, Sulfakinin and Myosuppressin, present unique sequences among the members of their families in insects. As in *R. prolixus* we did not find Arginine-Vasopressin-like peptide nor Sex peptide, but we identified a bona fide Sex Peptide receptor. The Prothoracicotropic hormone (PTTH) was also identified (see section 5.1.a). The Adipokinetic hormone (AKH) was not found, although its receptor. We cannot rule out gaps in the genome sequence, or highly diverging sequences that were not detected by our homology search.

Table S 5.17: Summary information for annotated neuropeptides and their receptors (in Excel Supplement).

5.3.e Visual genes and light detection

Contributors: Markus Friedrich, Jeffery W. Jones, Megan Porter

The milkweed bug has been one of the earliest model systems to study basic mechanisms of pattern formation during insect compound eye development [195]. The milkweed bug compound eye has also been used for a variety of physiological analyses [196], but more detailed analyses of spectral sensitivities across the compound eye exist for other hemipteran species, most notably the water striders and, most recently, cicadas [197-199]. Together with the first molecular study of opsin diversity in the vetch aphid *Megoura viciae* [200], these studies produced direct evidence of long wavelength sensitive (LWS), blue short wavelength sensitive (SWS-B), and UV short wavelength sensitive (SWS-UV) opsin subfamilies in the Hemiptera.

The genomic analysis of opsin diversity in *Oncopeltus* recovered two, tandemly duplicated, LWS opsin homologs and one UV-opsin, but failed to detect sequence evidence of an SWS-B opsin (Figure S 5.17 and Table S 5.18). The results from equivalent genomic analyses in further representatives of the Heteroptera (*Cimex lectularius*, *Rhodnius prolixus*) as well as representatives of other hemipteran suborders (Sternorrhyncha: *Pachypsylla venusta*, *Acyrthosiphon pisum*) suggest that SWS-B opsin was lost early in the last common ancestor of Heteroptera, while the LWS opsin duplication is unique for *O. fasciatus* in hemipteran species. While this points to a relatively recent time point of their origin, their protein sequences have substantially diverged to 71% protein sequence identity. In addition to these retinal opsin subfamilies, the *O. fasciatus* genome harbors two extra-retinal opsins. This includes a representative of the enigmatic but deeply conserved Rh7 opsin subfamily that has been described in *D. melanogaster* but not yet been functionally characterized [201]. The second extra-retinal opsin is a member of the recently characterized arthropsin subfamily, which was first reported from the *Daphnia* genome but has since then been detected in other arthropods including insects such as the pea aphid [202-204]. The otherwise sketchy detection in hemipteran genomes was complemented by the discovery of an ortholog in the genome draft of the Asian citrus psyllid *Diaphorina citri* (Figure S 5.17), cementing the evidence for arthropsin conservation in the Hemiptera. Given the close relationship of the Hemiptera to the

Holometabola and the pervasive absence of arthropsin in the well-curated genomes of the latter, it is reasonable to conclude that arthropsin was lost in the last common ancestor of the Holometabola. Understanding the phenotypic consequences of this gene loss will await the functional studies of arthropsin in versatile direct developing insect models like the milkweed bug. The more broadly conserved extraretinal c-opsin subfamily could not be detected in the milkweed bug. However, orthologs in *Cimex lectularius* and *Rhodnius prolixus* as well as the pea aphid document its conservation in the Hemiptera (Table S 5.18).

Table S 5.18: Opsin conservation in Hemiptera.

SPECIES	Order	Suporder	LWS	SWS-B	SWS-UV	Rh7	ARTHROPSIN	C-OPSIN
Oncopeltus fasciatus	Hemiptera	Heteroptera	2	-	1	1	1	-
Cimex lectularius	Hemiptera	Heteroptera	1	-	1	1	-	1
Rhodnius prolixus	Hemiptera	Heteroptera	1	-	1	1	-	1
Pachypsylla venusta	Hemiptera	Sternorrhyncha	1	1	1	1	-	-
Acyrthosiphon pisum	Hemiptera	Sternorrhyncha	1	-	2	4	1	1
Megoura viciae	Hemiptera	Sternorrhyncha	1	-	1	na	na	na
Nephotettix cincticeps	Hemiptera	Auchenorrhyncha	1	1	1	na	na	na

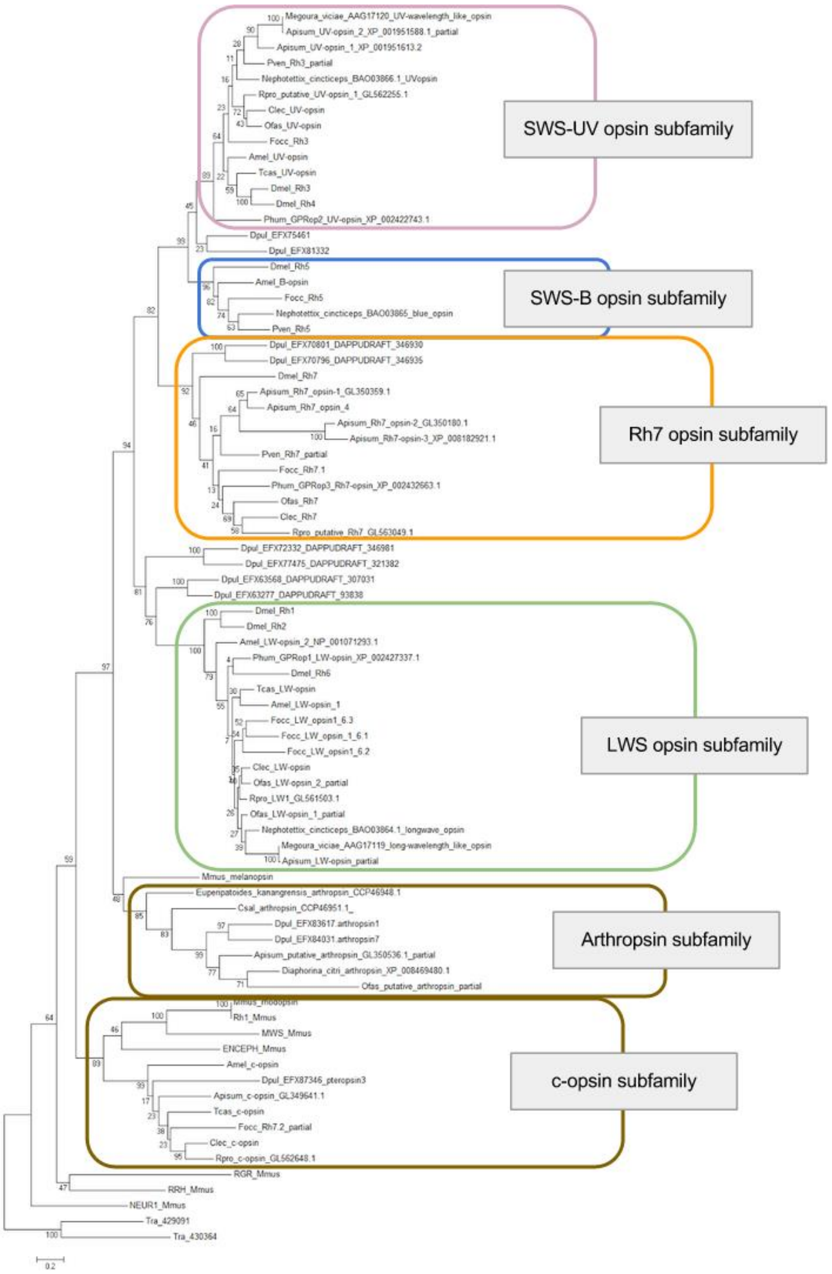

Figure S 5.17: Nonparametric bootstrap maximum likelihood tree of opsin genes from hemipteran and other arthropod species. Protein sequences were aligned with Webprank [205]. Ambiguous alignment regions were filtered using TrimAl (v. 1.3) [206] as implemented on the Phylemon 2.0 server [207] applying User defined settings (Minimum percentage of positions to conserve: 10, Gap threshold: 0.9, Similarity threshold: 0.0, Window size: 1.0), resulting in 267 multiple alignment positions for further analysis. Maximum likelihood tree was estimated in MEGA version 6.0 [66] applying the Jones-Taylor-Thornton (JTT) model of amino acid sequence evolution and assuming Gamma Distributed substitution rates across sites with 3 categories. Numbers at branches represent non-parametric bootstrap support from 100 replications. Abbreviations of species that were investigated using genome draft annotations: Amel, *Apis mellifera*; Apisum, *Acyrthosiphon pisum*; Clec, *Cimex lectularius*; Dmel, *Drosophila melanogaster*; Dpul, *Daphnia pulex*; Focc, *Frankliniella occidentalis*; Mmus, *Mus musculus*; Ofas, *Oncopeltus fasciatus*; Phum, *Pediculus humanus*; Rpro, *Rhodnius prolixus*; Pven, *Pachypsylla venusta*; Tcas, *Tribolium castaneum*. Outgroup sequence species: Tra, *Trichoplax adhaerens*. Alignment available on request.

5.3.f Chemoreceptors

Contributor: Hugh M. Robertson

The gustatory receptor (GR) family of seven-transmembrane proteins in insects mediates most of insect gustation (*e.g.*, [208, 209]), as well as some aspects of olfaction, for example, the carbon dioxide receptors in flies [210-213]. The GR family ranges in size from a low of 6 genes encoding 8 proteins in the human body louse [214] and 10 genes in the honey bee *Apis mellifera* [215] to 215 genes encoding 245 proteins in the flour beetle *Tribolium castaneum* [57]. The other sequenced hemipteroid insects have intermediate sized families, with 77 genes in the pea aphid *Acyrthosiphon pisum* [216], 28 genes encoding 30 proteins in the kissing bug *Rhodnius prolixus* [217], and 24 genes encoding 36 proteins in the bed bug *Cimex lectularius* [19]. The GR family is more ancient than the OR family, which was clearly derived from within it, and is found in the crustacean *Daphnia pulex* [218], the mite *Metaseiulus occidentalis* [219], the tick *Ixodes scapularis* (HMR, unpublished), and many other animals (HMR, unpublished). This evolutionary history is reminiscent of the more recently described ionotropic receptors (IRs) [220-222], some of which also probably function in gustation.

Methods

TBLASTN searches of the genome assembly were performed using *Cimex*, *Rhodnius*, *Acyrthosiphon*, *Pediculus*, and *Drosophila* proteins as queries, and gene models were manually assembled in a text editor (TEXTWRANGLER). Iterative searches were conducted with each new *Oncopeltus* protein as query until no new genes were identified in each major subfamily. All of the *Oncopeltus* genes and encoded proteins are detailed in Table S 5.19-5.21, and all *Oncopeltus* proteins are provided in a supplementary FASTA file (Additional File 3). The gene models for these have been updated in the Apollo genome browser as best possible (however the OR and IR updates are not included in OGSv1.2 and will only appear in later releases of the genome annotation).

The somewhat fractured nature of the genome assembly meant that there are many gene fragments that were not annotated in each family, however we endeavored

to include as many as we believe represent intact genes, even if they did not always encode more than 50% of a typical receptor protein. In some cases, for example OrCo, some ORs (Or40, 71, 110, and 114), and some IRs (Ir25a, 40a, 75c, and 75j), the ends of two scaffolds have interdigitated contigs that encode alternating exons or sets of exons, and these and a few other examples of genes that span scaffolds were built. Proteins were aligned in CLUSTALX v2.0 [223] using default settings, and problematic gene models were refined in light of these alignments.

Most *Cimex*, *Rhodnius*, *Acyrthosiphon*, and *Pediculus* GRs (excluding PhOr11 and 12 which are too short to include and ApisGr12 and 15 as they are so divergent they disrupt the alignments), as well as select other insect GRs, were included in the alignments for the phylogenetic analysis. The final alignments were trimmed using the "gappyout" option in TRIMAL v4.1 [206]. Maximum likelihood analysis was performed using PHYML v3.0 [224] with default settings. The tree figures were prepared using FIGTREE v1.4 (http://tree.bio.ed.ac.uk/software/figtree/) and Adobe Illustrator.

Results

The OR family

The OR family consists of the highly conserved and generally single-copy OrCo gene and 102 specific OR genes. The OrCo gene, like some of the IR genes below, is split across two scaffolds with exons on the ends of the scaffold separated by gaps and interdigitated with each other (Table S 5.19), as are four of the specific OR genes. The specific OR genes are generally a lot smaller, and most were intact, however many have terminal or internal exons missing. Some of these could be repaired using the available whole body RNAseq, specifically raw reads that did not map to the assembly but are available in the Short Read Archive at NCBI. One OR gene was modeled as alternatively spliced with two protein products (Or88), so the total of potentially encoded ORs is 121, and only two of these is pseudogenic, leaving 119 potentially active ORs. There are, however, several additional fragments of OR genes in the assembly, some of which may represent intact genes, while some incomplete models might actually be pseudogenes.

The phylogenetic tree is rooted with the conserved OrCo proteins (Figure S

5.18), and reveals that there is a complex web of relationships of the specific ORs across these four hemipteroids. Most of the *Pediculus* and *Acyrthosiphon* ORs form distinct clades or subfamilies, while *Oncopeltus*, *Cimex*, and *Rhodnius* ORs sometimes reveal potentially orthologous relationships. Nevertheless, the majority of large OR expansions are species-specific, and these are mostly in *Oncopeltus* and *Rhodnius*, with *Cimex* having only a few small expansions (Figure S 5.18). The largest *Oncopeltus* expansion is 36 genes (OfasOr24-59). Unlike the pea aphid repertoire, which revealed several recent expansions exhibiting signals of positive selection on some amino acid positions [216], the *Oncopeltus* ORs appear to be older expansions with relatively longer branches to most proteins.

The GR family

The GR gene set consists of 115 models, encoding 169 proteins (Table S 5.20), considerably larger than that of many other insects. Of these only 9 are clearly pseudogenic, but many models are currently missing termini or internal regions in gaps in the assembly, so their status remains uncertain. There are many genes modeled as alternatively spliced, in a fashion common to the GR family in several other insects, that is, with two or more long first exons spliced into shared C-terminal exons, although in most cases in the absence of transcriptome evidence these models remain hypothetical. The MAKER modeling had access to all available insect GRs in GenBank, for comparative information, and succeeded in building at least partial gene models for 20 of these 115 loci, while AUGUSTUS had partial models for many more, nevertheless all models required at least one change.

Oncopeltus has seven genes encoding proteins related to the highly conserved carbon dioxide receptors of flies and other insects [225], and these were named Gr1-7 (Figure S 5.19). They cluster phylogenetically with four similar proteins from the *Cimex*. This carbon dioxide lineage is absent from all Hymenoptera sequenced to date, as well as *Acyrthosiphon*, *Pediculus*, and *Rhodnius* [214, 216, 217], so appears to have been lost repeatedly. A large related subfamily expansion was discovered in the termite *Zootermopsis nevadensis* [226], indicating that this gene lineage is indeed ancient in insects. It remains to be shown whether they participate in perception of carbon dioxide.

Oncopeltus has three genes encoding candidate sugar receptors, named Gr8-10

(Figure S 5.19). This subfamily is absent from the blood-sucking hemipterans examined to date (*Pediculus*, *Rhodnius*, and *Cimex*), but is present as six genes in the plant-attacking pea aphid [19, 214, 216, 217]. The only other conserved Gr is Gr11, which is an ortholog of the DmGr43a fructose receptor [227], and is also present as a single ortholog in the other hemipteroids, except *Pediculus*.

The remaining *Oncopeltus* GRs (12-115) are quite divergent from any of the conserved GRs, and consist of several gene lineages, most of which are closer to the equivalent *Cimex* and *Rhodnius* GRs than *Pediculus* or *Acyrthosiphon* (Figure S 5.19). These include all of the alternatively spliced models. The long branches to most of these proteins are similar to those to the *Cimex* and *Rhodnius* proteins, and in stark contrast to most of the aphid GRs, which form several recently expanded gene subfamilies that reveal evidence of positive selection of amino acids indicative of adaptive divergence [216]. Most of the other *Drosophila* GRs are implicated in perception of bitter tastants [228, 229], however it is hard to be confident of such a function for these *Oncopeltus* GRs and their *Cimex/Rhodnius* relatives. It is nevertheless somewhat surprising that *Oncopeltus* has such a large repertoire of GRs. It implies that they are adapted to sense a wide range of bitter taste chemicals, presumably employed in selecting suitable host plants.

The IR family

The IR family consists of at least 37 genes (Table S 5.21and Figure S 5.20). It contains two highly conserved receptors (Ir8a and 25a) that are closely related to the ionotropic glutamate receptors from which they evolved (see [221, 226]), and which serve as co-receptors with the other IRs, along with Ir76b [222]. Another group of receptors (Ir21a, 40a, 68a, and 93a) are present in fairly conserved single orthologs, as is the case for most other insects, and several of these have recently been shown to be involved in perception of temperature and humidity in Drosophila [230-232]. The Ir41 and 75 lineages consist of multiple genes in most insects, and in *Oncopeltus* there are three and ten genes, respectively. In Drosophila they are involved in perception of acids and amines [233, 234]. Following an approach begun with the termite *Zootermopsis nevadensis* [226], and applied to *Rhodnius* and *Cimex*, the conserved IRs are named for their *Drosophila* orthologs, the Ir41 and 75 lineages are named with suffices a-c and a-j, while the divergent genes below are numbered from 101 to

avoid any confusion with the Drosophila IR names which only go to 100a (because they were named for their cytological location in that genome). There are several lineages of highly divergent receptors, and in *Oncopeltus* there are 17 of these, Ir101-117, dispersed around the tree with relationships with similarly divergent IRs in the other hemipteroid insects. Ir113-117 have multiple exons like the more conserved IRs, while the other two divergent lineages are primarily intron-less genes, with three of them acquiring idiosyncratic introns. This pattern of mostly intron-less genes is typical for the divergent IRs in other insects, and if the roles of the divergent IRs in Drosophila, and in particular the large Ir20a clade, are a guide [222, 235, 236], they might function as taste receptors.

These *Oncopeltus* IR genes were particularly difficult to annotate, despite the fact that the relatively conserved genes have clear homologs in other species, and most of the divergent receptors are intron-less. The fractured nature of the assembly, combined with the fact that they are generally large genes spanning 10-30 kb and have many short exons, caused most of the problems, ranging from exons missing in gaps, to genes split across scaffolds, including three instances of exons on different scaffolds being interdigitated with each other, and sometimes with exons in incorrect order. Luckily the combination of relatively conserved sequence and often substantial RNAseq support allowed construction of fairly complete models for most genes, however many of them cannot be properly modeled in the Apollo genome browser. Thus 14 of the 23 genes with multiple introns required repair of the assembly, commonly using raw RNAseq reads (the RNAseq reads mapped in Apollo are not useful when an exon is missing or a gene is split across scaffolds or otherwise misassembled), as well as raw genome reads (Table S 5.21). Official models existed for at least parts of most of the relatively conserved genes, but the divergent genes, despite commonly being intronless, were usually only represented in the AUGUSTUS gene set. Altogether 22 new gene models were created (Table S 5.21).

Thirteen of the IR models remain incomplete, primarily at the N-terminus, in large part because their N-termini are commonly highly divergent hence difficult to identify using TBLASTN searches, are hard to be confident about from de novo gene building, and the available RNAseq seldom extends to the N-terminus. Just two genes are clear-cut pseudogenes (both divergent IRs), at least in the assembled genome, but both are caused by single problems (one stop codon and one frame shift), so both might be intact genes in other populations or even pseudo-pseudogenes [233]. Finally,

the Ir113-117 lineage of genes is particularly difficult to model, being divergent and having little to no RNAseq support. TBLASTN searches with these five proteins reveal at least three more related genes that are present only as a few exons each and are too difficult to model in the absence of RNAseq support, so the total number of IRs in *Oncopeltus* is likely to be 40.

This repertoire of IRs is slightly larger than the other sequenced hemipteroids, with 19 in the pea aphid [32], 33 in the kissing bug [217], 30 in the bed bug [19], and 14 in body louse [214], although other insects commonly have more IRs, such as *Drosophila melanogaster* with 65 [221] and the termite *Zootermopsis nevadensis* with 150 [226],. The only obvious differences are in the numbers of the divergent lineages, which at least in *Drosophila* are implicated in gustation rather than olfaction [222, 235, 236]. This small potential gustatory receptor IR expansion in *Oncopeltus* is in line with the expansion of its GR family.

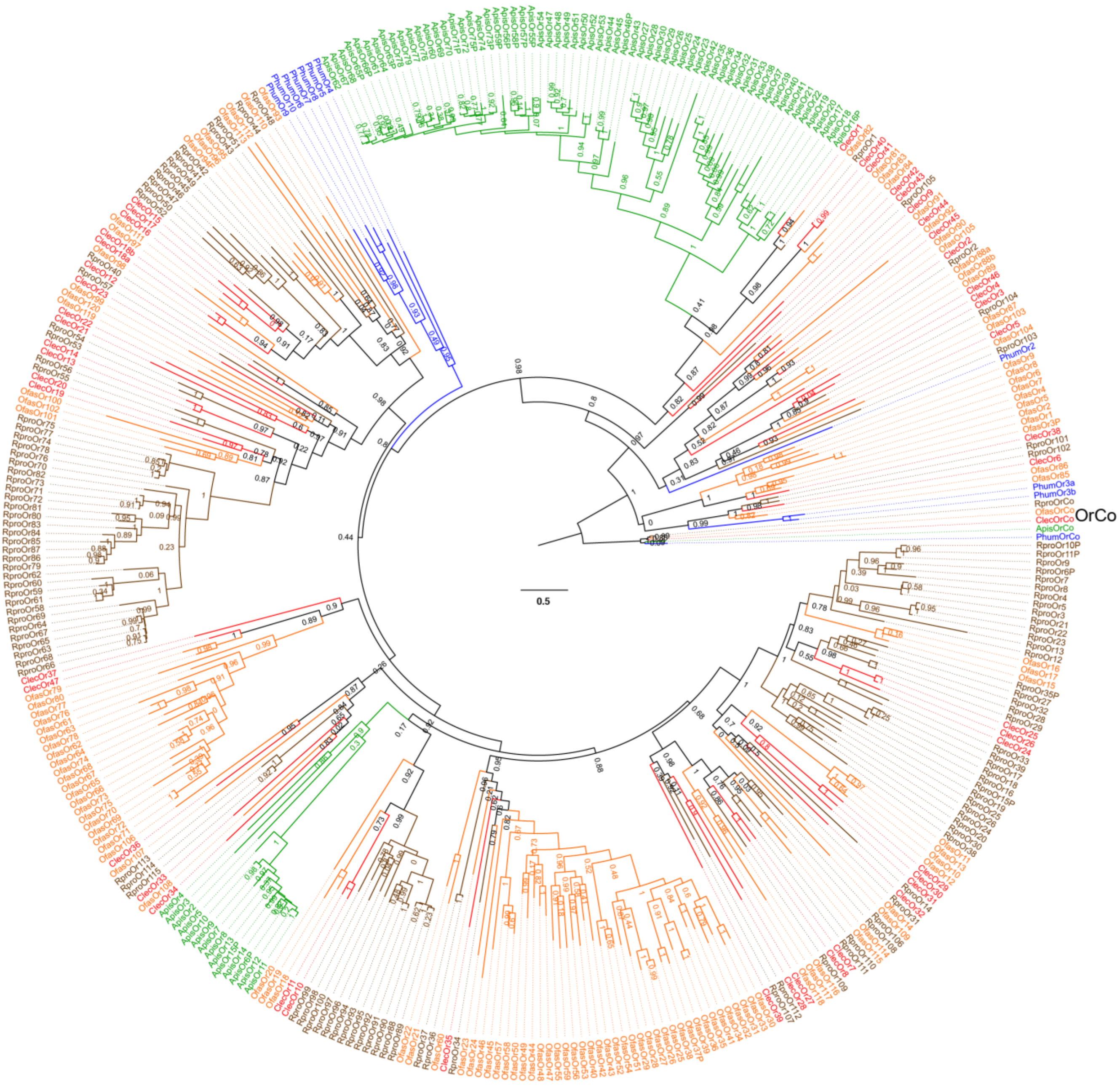

Figure S 5.18: Maximum likelihood phylogenetic tree of the *Oncopeltus*, *Cimex*, *Rhodnius*, and *Pediculus* ORs. The tree was rooted with the highly conserved and basal OrCo proteins. The *Oncopeltus*, *Cimex*, *Rhodnius*, *Acyrthosiphon*, and *Pediculus* gene/protein names are highlighted in orange, red, brown, green, and blue, respectively, as are the branches leading to them to emphasize gene lineages. Lowercase letters distinguish different protein isoforms from a single gene. Suffixes after the gene/protein names are: P – pseudogene; N – N-terminus missing; C – C-terminus missing; I – internal sequence missing; F – sequence fixed with raw reads; J – gene model joined across scaffolds.

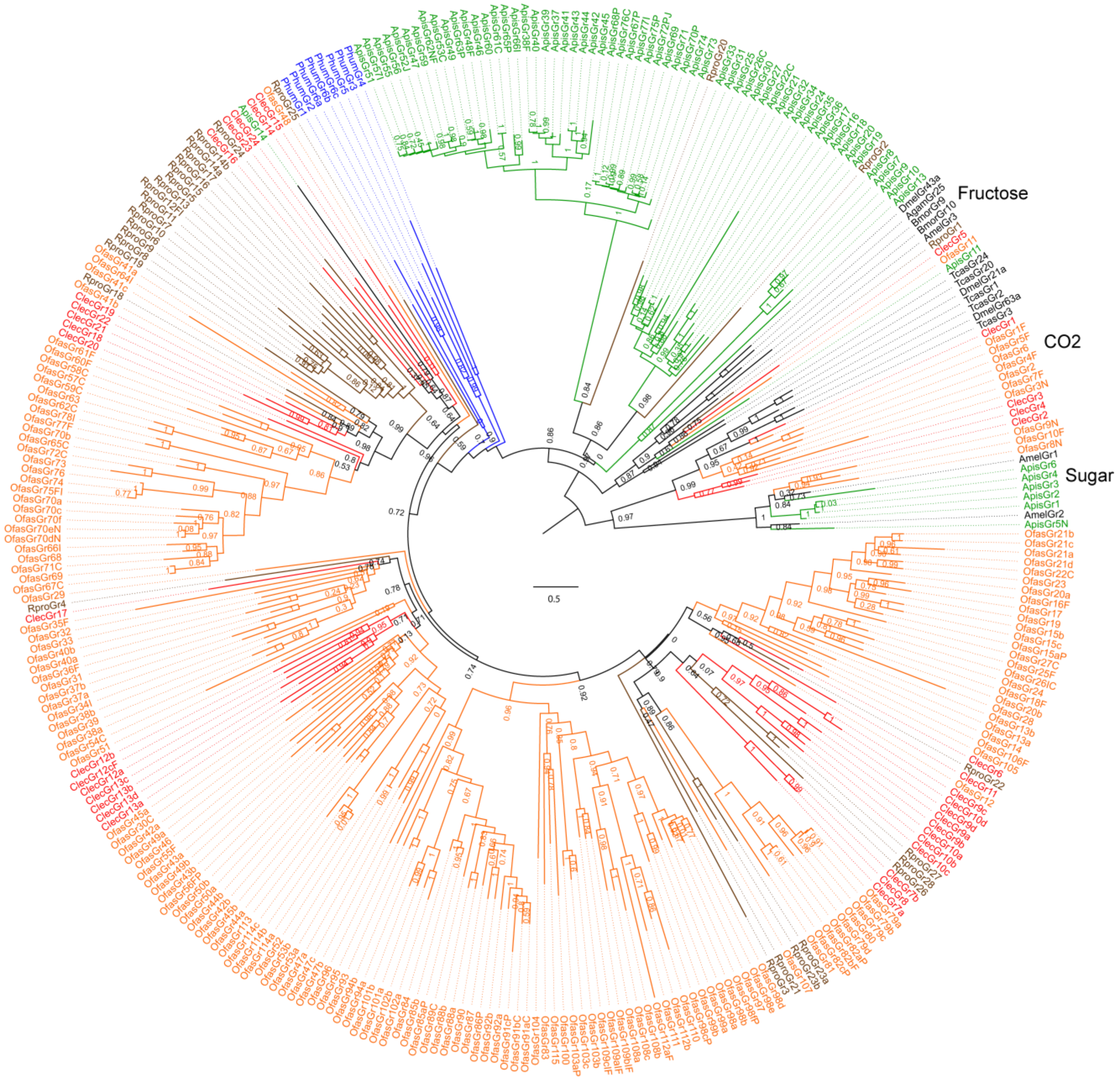

Figure S 5.19: Maximum likelihood phylogenetic tree of the *Oncopeltus*, *Cimex*, *Rhodnius*, *Acyrthosiphon*, and *Pediculus* GRs, and representative GRs from other insects. The tree was rooted with the candidate carbon dioxide and sugar receptors, subfamilies of conserved GRs that are divergent from the other GRs. See legend for Figure S 5.18 for other details.

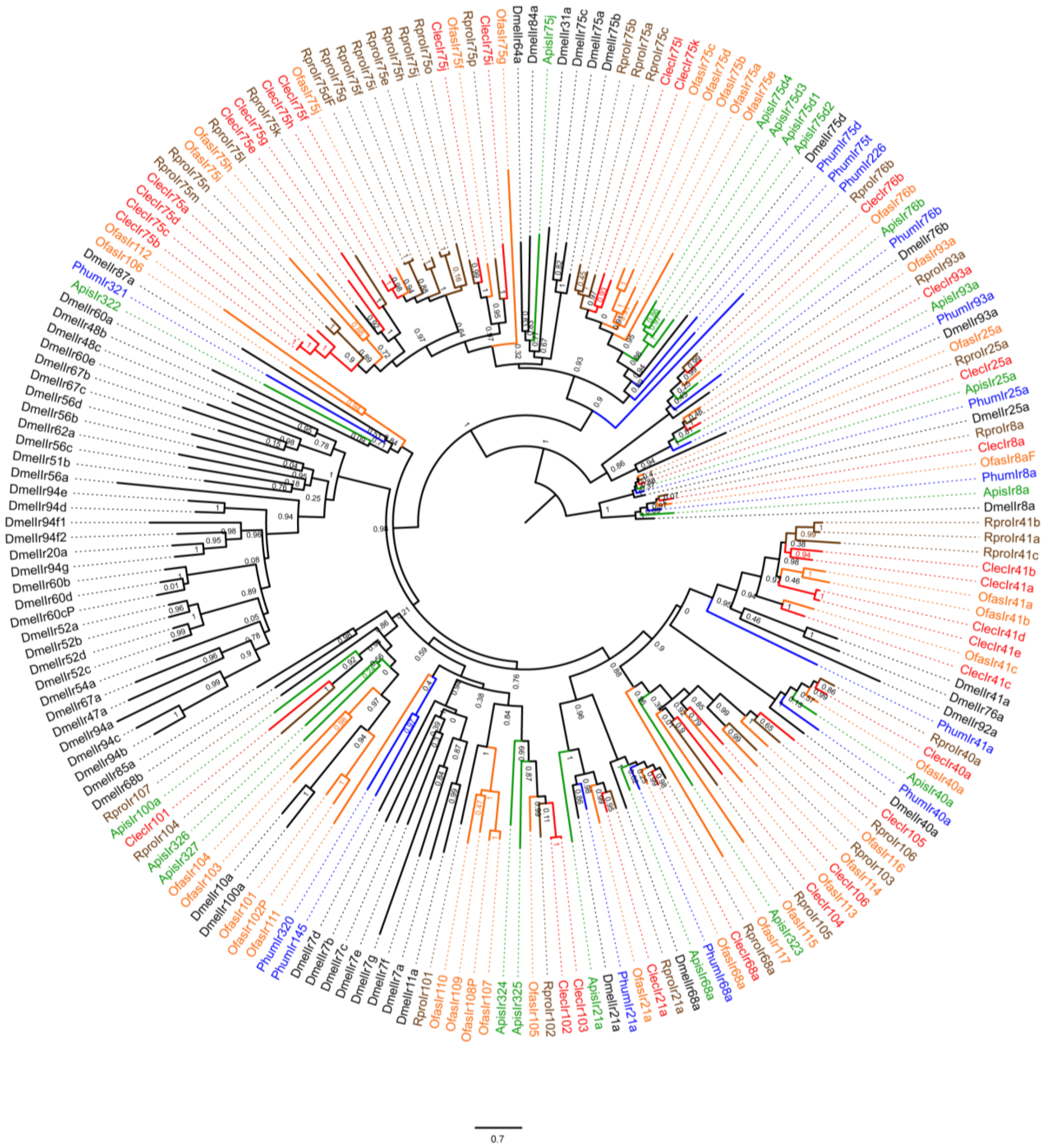

Figure S 5.20: Maximum likelihood phylogenetic tree of the *Oncopeltus*, *Cimex*, *Rhodnius*, *Acyrthosiphon*, *Pediculus*, and *Drosophila* IRs. The tree was rooted with the conserved and basal Ir8a/25a proteins. See legend for Figure S 5.18 for other details.

Table S 5.19: Details of OfasOr family genes and proteins (in Excel Supplement).

Table S 5.20: Details of OfasGr family genes and proteins (in Excel Supplement).

Table S 5.21: Details of OfasIr family genes and proteins (in Excel Supplement).

Additional file 3: Chemoreceptor sequences in FASTA format. (TXT file)

Note that due to the fragmented nature of the genome assembly, the chemoreceptor gene models were predicted independently from the assembly by manually combining the gene predictions with RNA-seq data. Thus, some gene locus coordinates provided in the supplementary tables (S5.19-21) may vary from the ones in theOGSv1.2 gff file. However, the complete curated protein sequences for all these models are provided in the supplementary fasta file ("Ofas-chemoreceptors-protein-seqs.fasta"), with sequences for 121 Or, 169 Gr, and 37 Ir proteins. In the file, suffixes after the gene/protein names are: C - C-terminus missing; F – assembly sequence fixed with raw reads or other repairs; I – internal region missing; J – model joined across scaffolds; N – N-terminus missing; P – pseudogene (Z – stop codon and X- frameshift or other major problem).

5.4 Molecular machinery

5.4.a Gene silencing machinery (RNAi, miRNA, piRNA)

Contributors: Yi-min Hsiao, Hsiao-ling Lu, Chun-che Chang

In *Oncopeltus* we have identified sequences encoding conserved components of the different post-transcriptional gene silencing pathways: RNA interference (RNAi), micro-RNA (miRNA), and piwi-interacting RNA (piRNA). These genes include *Drosha*, *partner of drosha* (*pasha*), *Dicer 1* (*Dcr1*), *Dcr 2*, *Argonaute 1* (*Ago1*), *Ago2*, *Ago3*, and *piwi*/*aubergine* (*piwi*/*aub*) (Table S 5.22). Like *Drosophila melanogaster* (fly) and *Tribolium castaneum* (beetle), where most of these genes are not duplicated, *Oncopeltus* only has a single copy of each gene (Table S 5.23), which is also the case in the fellow hemipterans the bed bug [19] and the soybean aphid [237]. In contrast, *Acyrthosiphon pisum* (aphid) has a notable expansion of the miRNA and piRNA machinery [32, 149, 238].

Table S 5.22: Major components RNAi/miRNA machinery in *Oncopeltus fasciatus.*

Gene name	Gene abbreviation	Number of copies found in *Oncopeltus*	Comments
**Gene name**	**Gene abbreviation**	**Number of copies found in *Oncopeltus***	**Comments**
Drosha	*Drosha*	1	
partner of drosha	*pasha*	1	
Dicer 1	*Dcr1*	1	CDS splits in three different scaffolds
Dicer 2	*Dcr2*	1	CDS splits in two different scaffolds
Argonaute 1	*Ago1*	1	
Argonaute 2	*Ago2*	1	
Argonaute 3	*Ago3*	1	
piwi	*piwi*	1	See annotation of germline genes.

Table S 5.23: Orthologous numbers of RNAi machinery components in four insect species.

Gene	Fruit fly	Beetle	Pea aphid	Milkweed bug
Drosha	1	1	1	1
pasha	1	1	4	1
Dcr1	1	1	2	1
Dcr2	1	1	1	1
Ago1	1	1	2	1
Ago2	1	2	1	1
Ago3	1	1	2	1
piwi	1	1	8	1*

*See also the annotation of germline genes in Table S 5.7.

5.4.b Sex determination and dosage compensation

Contributors: Subba (Reddy) Palli, Jayendra N. Shukla

O. fasciatus is a male heterogametic (XX-female and XY-male) insect [239], but the sex determination signal remains unclear. The most downstream gene of *Drosophila* sex determination cascade, *doublesex* (*dsx*), is conserved in all the insects studied so far [240] as inferred from the studies in various groups of holometabolous insects [241], and is the founding member of proteins with DM domains [242-244]. However, the *Oncopeltus* genome contains DM superfamily genes in which no Oligomerization (OD2) domain was identified. This is similar to the case of two other hemipterans, *Acyrthosiphon pisum* and *Rhodnius prolixus*, whose genomes have been sequenced recently [32]. Functional analysis of DM domain genes in hemimetabolous insects is required to ascertain their potential roles in sex determination.

Homolog of *transformer* (*tra*), the upstream regulators of *dsx* in holometabolous insects (absent in lepidopterans and basal dipteran lineages, *i.e.*, mosquitoes [241]) is present in *Oncopeltus* genome. Interestingly, the partial *tra* homolog obtained showed high sequence conservation in the auto regulation domain of the hymenopteran *tra* sequence (Figure S 5.21 A,B). Whether *Oncopeltus tra* is spliced in a sex specific manner and regulates the splicing of its own and *dsx* pre-mRNA needs further investigation. Among other core sex determination genes homologs, *transformer-2* [245], *intersex* [246], *fruitless* [247] and *P-element somatic inhibitors* [248] have also been identified in the *Oncopeltus* genome. Figure S 5.21 C represents the schematic diagram of the proposed sex determination cascade in *Oncopeltus fasciatus*.

The dosage compensation mechanism in *Drosophila* is known to equalize the dose of X chromosome linked transcripts. Out of five major genes for dosage compensation in *Drosophila* (*msl-1*, *msl-2*, *msl-3*, *mle* and *mof*) [249] homologs of *msl-2*, *msl-3*, *mle* and *mof* have been identified in the *Oncopeltus* genome, suggesting the existence of a functional dosage compensation pathway.

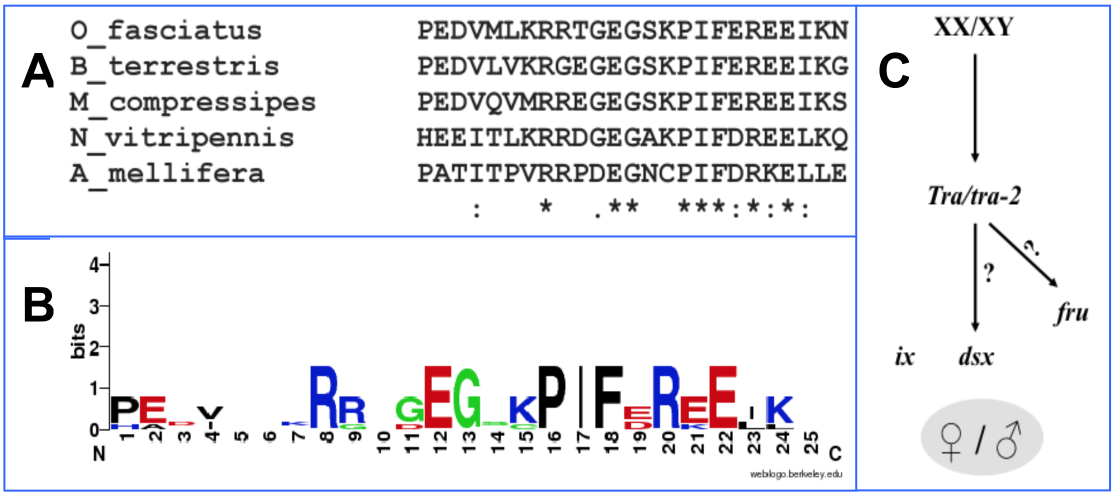

Figure S 5.21: **(A)** CLUSTAL alignment (http://www.ebi.ac.uk/Tools/msa/clustalo/) of putative *tra* auto regulation domain of *O. fasciatus* and that of other hymenopteran insects (*B. terrestris*, *M. compressipes* , *N. vitripennis*, *A. mellifera*) (please see [250]). **(B)** WebLogo (http://weblogo.berkeley.edu/logo.cgi) of putative *tra* auto regulation domain of *O. fasciatus* and that of other hymenopteran insects (*B. terrestris*, *M. compressipes*, *N. vitripennis*, *A. mellifera*) displaying a deep conservation in the sequence (see [250]). **(C)** Putative sex determination pathways in *Oncopeltus fasciatus* based on the sex determination cascade of holometabolous insects [251]. The homologs of sex determining genes *doublesex* (*dsx*), *transformer* (*tra*), *transformer-2* (*tra-2*), *fruitless* (*fru*) and *intersex* (*ix*) are present in the genome of *Oncopeltus fasciatus*.

5.4.c Epigenetic machinery

Contributor: Elizabeth J. Duncan

DNA methylation and post-translational modifications of histones are key regulators of chromatin structure and of gene expression, and these epigenetic systems have been associated with environmental responsiveness and phenotypic plasticity [252].

DNA methylation

DNA methylation is associated with gene silencing in vertebrates [253] and alternative splicing of mRNA [19, 254-256]. Functional DNA methylation systems have been demonstrated in a few insects including the honeybee *Apis mellifera* [255, 257, 258] and the hemipteran *Acyrthosiphon pisum* (the pea aphid) [259].

Like these insects, *Oncopeltus* also appears to have an intact DNA methylation system. The *Oncopeltus* genome encodes two copies of the maintenance methyltransferase *Dnmt1*, the *de novo* DNA methyltransferase *Dnmt3*, and a copy of *Tet1* (*Ten-eleven translocation methylcytosine dioxygenase 1*) that has been implicated in removing methylation marks by converting 5′ methylcytosine to 5′ hydroxymethylcytosine [260, 261].

In insects and other invertebrates DNA methylation (the addition of a methyl group to a cytosine residue in a CpG context) occurs predominately on gene bodies (exons and introns) [262-264]. In vertebrates DNA methylation occurs in CpG islands in the promoter regions of genes and is associated with gene silencing [265]. Gene body methylation also occurs in vertebrates and it has been shown to be as abundant as methylation in CpG islands [262]. The function of gene body methylation is currently unknown, however gene body methylation has been correlated with active transcription in a wide range of species [264], has been implicated in alternative splicing [254, 255] and regulating chromatin organization [266].

Over evolutionary time methylation of cytosine residues leaves them susceptible to deamination to uracil, which is repaired as a thymine, leaving methylated genes with a relatively low CpG content [267]. The CpG content can be measured mathematically as: $CpG_{[o/e]}$ = number of CpG dinucleotides in a gene/[the number of C nucleotides]×[the number of G nucleotides]. In animals without DNA

methylation, such as *Drosophila melanogaster*, a unimodal distribution of CpG content is observed (Figure S 5.22 A). In contrast, a bimodal distribution is seen in insects with an active methylation system (for instance *Apis mellifera* and *Acyrthosiphon pisum*, Figure S 5.22 B,C).

In the honeybee it has been found that there is a significant correlation between genes that have low $CpG_{[o/e]}$ (*i.e.*, are predicted to be historically methylated) and genes that are currently methylated in the brains of honeybees [255], confirming that $CpG_{[o/e]}$ is a good predictor of genes that are currently methylated.

Methods

Gene body and intragenic sequences were extracted from the predicted *Oncopeltus* gene set (OGS v1.1) using CLC Genomics Workbench (version 7). For analysis of whole genome and intragenic regions, the sequences were split into 1000-nt non-overlapping fragments, and nucleotide and dinucleotide content of gene body sequences and whole genome sequences were calculated as in previous analyses [268]. The number of components in these distributions was estimated in R (www.r-project.org) using mclust [269] model-based clustering. The best fitting model was identified among several non-nested models using Bayesian information criteria (BIC).

Results

Analysis of the CpG content of *Oncopeltus* gene bodies identifies a bimodal distribution of CpG[o/e] values, with a 'low' CpG[o/e] peak centered around 0.31 (55% of genes) and a 'high' $CpG_{[o/e]}$ peak around 0.71 (Figure S 5.22 D). This bimodal distribution is not observed with any other dinucleotide combination (Figure S 5.23). The presence of a low $CpG_{[o/e]}$ peak is consistent with historical DNA methylation as seen in other species (*e.g.*, Figure S 5.22 B,C). However, both peaks have relatively low CpG content, as almost all genes have a CpG[o/e] of less than one. This differs from other species where the high CpG[o/e] gene fraction has $CpG_{[o/e]}$ values greater than one (Figure S 5.22). This is consistent with an active DNA methylation system in *Oncopeltus*.

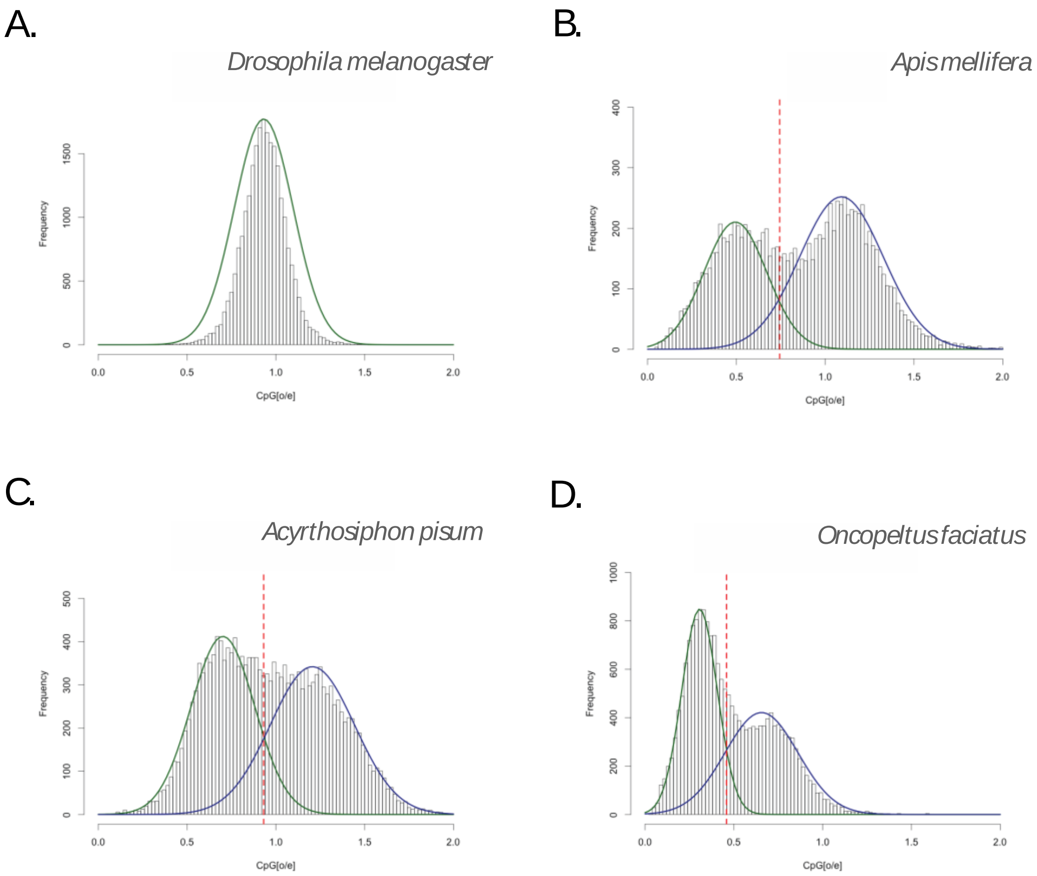

Figure S 5.22: Frequency histogram of CpG[o/e] observed in insect gene bodies. The y-axis depicts the number of genes with the specific CpG[o/e] values given on the x-axis. The distribution of CpG[o/e] in *Drosophila*, which does not have DNA methylation, is unimodal **(A)**. In contrast in species where DNA methylation is active, such as *Apis mellifera* **(B)** or the pea aphid **(C)** the distribution of CpG[o/e] is bimodal with genes that have been historically methylated having a lower than expected CpG content (green peak). *Oncopeltus* **(D)** genes also display a bimodal distribution of CpG content consistent with an active DNA methylation system in this species.

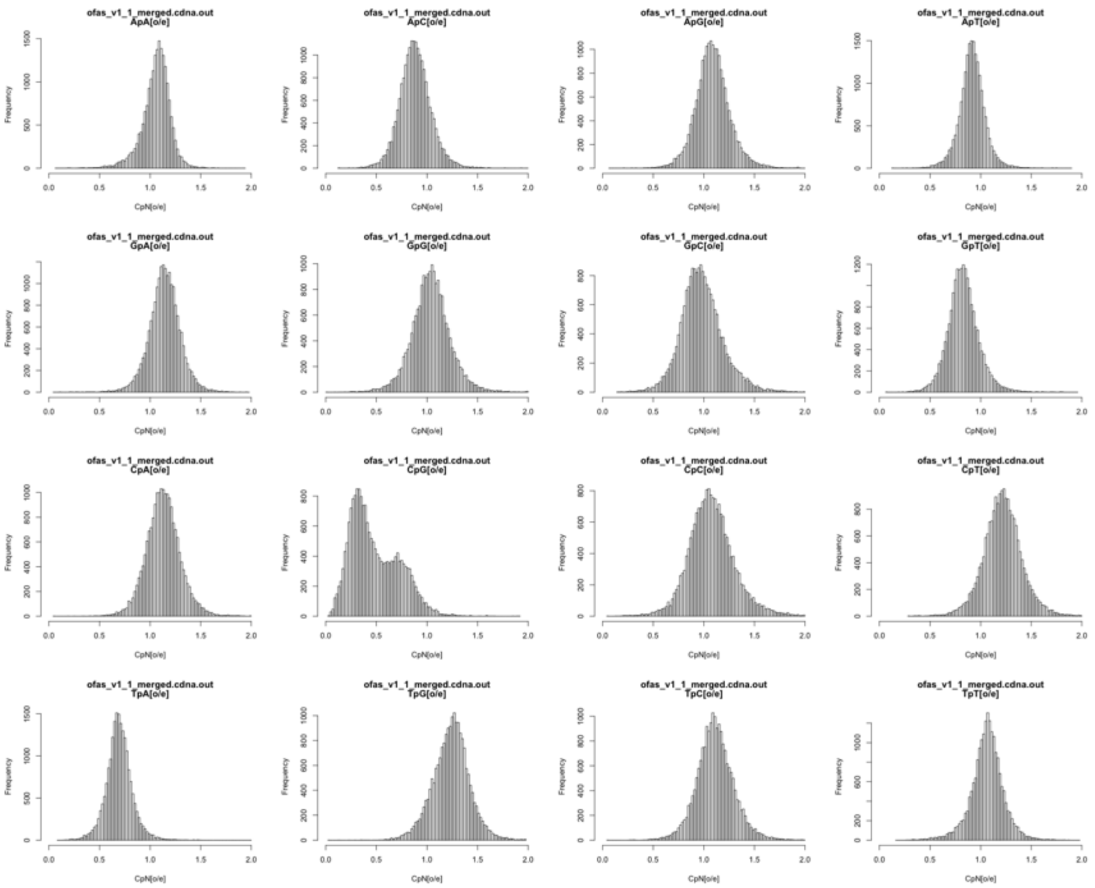

Figure S 5.23: Frequency histogram of the observed vs. expected ratio for all dinucleotide combinations in *Oncopeltus* gene bodies. The y-axis depicts the number of genes with the specific dinucleotide [o/e] values given on the x-axis.

To determine if this pattern of CpG frequency was unique to gene bodies, as it is in other species, intragenic regions were extracted and split into 1000-nt fragments. CpG[o/e] analysis of these intragenic regions demonstrates that a small number of intragenic DNA fragments (16.8%) fall into a region of the DNA with lower than expected CpG content (Figure S 5.24). This is not observed for any other dinucleotide combination (Figure S 5.25). This may be evidence for historical DNA methylation acting on regions of DNA that do not code for protein coding genes, regions encoding genes that have not yet been incorporated into the official gene set,, or other as yet unknown mechanisms that deplete CpG dinucleotide content independent of the GC content of the DNA.

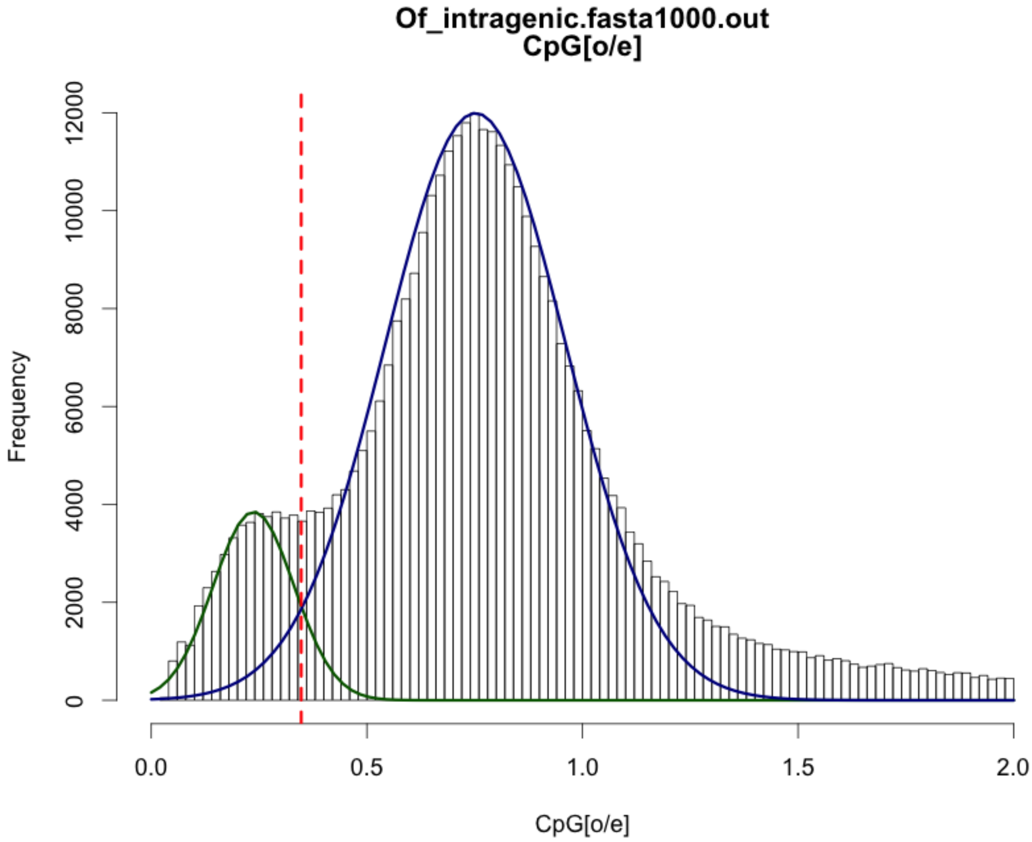

Figure S 5.24: Frequency histogram of CpG[o/e] observed in intergenic regions in *Oncopeltus*. The y-axis depicts the number of genes with the specific CpG[o/e] values given on the x-axis. The distribution is also bimodal suggesting that regions of the *Oncopeltus* genome are very CpG poor.

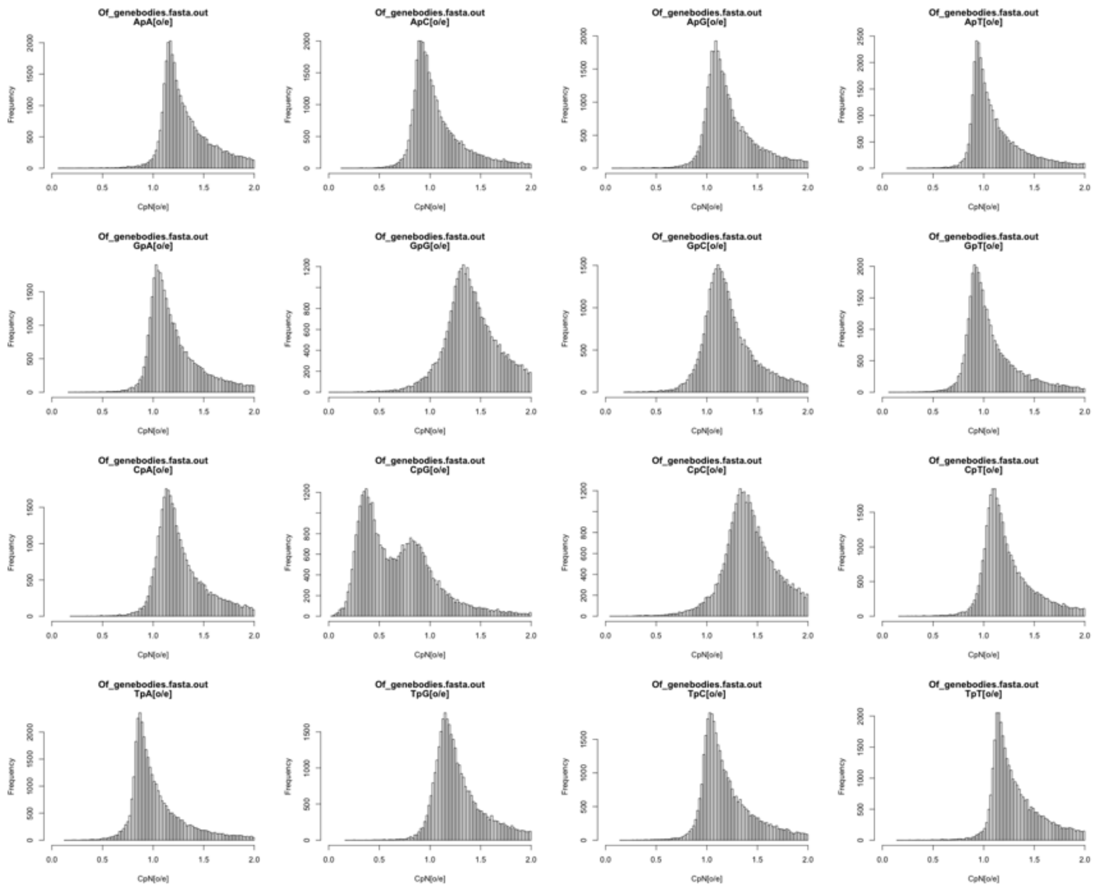

Figure S 5.25: Frequency histogram of the observed vs. expected ratio for all dinucleotide combinations in *Oncopeltus* intragenic regions. The y-axis depicts the number of genes with the specific dinucleotide [o/e] values given on the x-axis.

Histone encoding loci and histone modifying enzymes

The core unit of chromatin is the nucleosome, a highly conserved repeating unit composed of two copies of each of the four core histone proteins (H2A, H2B, H3, H4) assembled into an octamer and wound around 146-147 bp of DNA. The linker histone H1 binds the nucleosome and locks the DNA into place by binding the entry and exit sites of the DNA. These histones are post-translationally modified by methylation, acetylation and phosphorylation, dynamically influencing the structure of the chromatin. The *Oncopeltus* genome encodes fewer histone loci than any other arthropod genome (Tables S 5.24, 5.25). The linker histone, Histone H1, was initially not found in the assembly for the OGS v1.1, but it was subsequently identified from transcriptomic data, and a partial model for first exon was then identified in the assembly and incorporated into the OGS v1.2. This model is even missing a start codon due to a large gap directly upstream, which together with the missing sequence explains why the automated annotation pipeline missed it completely. *Oncopeltus* has two copies of the variant histone H2A.Z/H2.AV, which functions in environmental responsiveness and marking damaged DNA [270].

In *Drosophila* the histone genes are present in the genome in large numbers of quintet clusters, each cluster possessing one gene from each of the five classes of histone proteins. This arrangement of genes is also observed in other insects such as the pea aphid [271] and bed bug [19]. *Oncopeltus* does not have these quintet clusters and all of the histone genes are present as single copies on a scaffold.

The *Oncopeltus* genome encodes genes responsible for all classes of histone modifications; histone acetyltransferases, deacetylases, methylases and demethylases. Unusually there are duplications of the histone acetyltransferases *males absent on the first (mof), chameau (chm)* and *enoki mushroom (enok).* Duplications of *mof* and *enok* have only previously been reported for the pea aphid [271] and the bed bug (*Cimex lectularius)* [19]. Phylogenetic analysis indicates that the duplications of these genes in *Oncopeltus* are independent of the duplications in the aphid genome and in the bed bug genome.

Table S 5.24: Number of loci encoding the five classes of histones within the genomes of arthropod species. Orthologs for *A. aegypti*, *D. pulex*, *T. urticae* and *I. scapularis* were obtained by BLAST analysis. Orthologs for *A. mellifera* and *A. pisum* were obtained from published literature [255, 271].

Species	*H1*	*H2A*	*H2B*	*H3*	*H4*
Aedes aegypti	6	19	11	18	15
Apis mellifera	2	6	5	6	4
Acyrthosiphon pisum	6	5	5	7	5
Oncopeltus fasciatus	**1**	**3**	**4**	**3**	**2**
Cimex lectularius	4	14	6	13	8
Daphnia pulex	5	10	12	10	6
Tetranychus urticae	1	4	7	6	3
Ixodes scapularis	4	6	4	4	1
Strigamia	3	7	15	4	4

Table S 5.25: Histone and histone modifying genes identified in the *Oncopeltus* genome (in Excel Supplement).

5.4.d Repressive C2H2 zinc finger effectors (KAP-1/ TRIM proteins)

Contributor: Kristen A. Panfilio (KAP!)

Although the zinc finger 271-like subfamily in *Oncopeltus* shares a number of genomic, protein, and evolutionary features with the repressive KRAB-domain zinc finger protein families of vertebrates, we do not find evidence for an insect ortholog of the interaction partner KAP-1 (Figure S 5.26, [272], see also main text). In vertebrates, KRAB-associated protein 1 (KAP-1) acts as a chromatin-remodeling co-repressor, by recruiting methyltransferases (H3K9me) and deacetylases (H3K9ac and H3K14ac) to targeted genes' promoters (UniProtKB: Q13263). KAP-1, also known as TRIM28, is a member of the Tripartite motif-containing protein (TRIM) family.

Blast searches with human KAP-1 chiefly identified GenBank protein accessions designated as TRIM33 in insects and, in hemipteran genome assemblies, gene models encoding NHL repeat/ TRIM71 proteins. Thus, while insects do not have a direct ortholog of KAP-1, they do possess a homolog of the vertebrate protein family comprised of both KAP-1 and TRIM33 (Figure S 5.26: blue), where the latter is an E3 ubiquitin-protein ligase involved in BMP signaling repression in humans (UniProtKB: Q9UPN9). Curiously, the heteropteran TRIM28/33 homologs form a diverged outgroup (red) compared to other insect TRIM28/33 homologs, while genome tblastn searches with human KAP-1 rather identifies TRIM71-like homologs, which have predicted RNA-binding translational repression activity, in *Oncopeltus* and *Cimex* (grey).

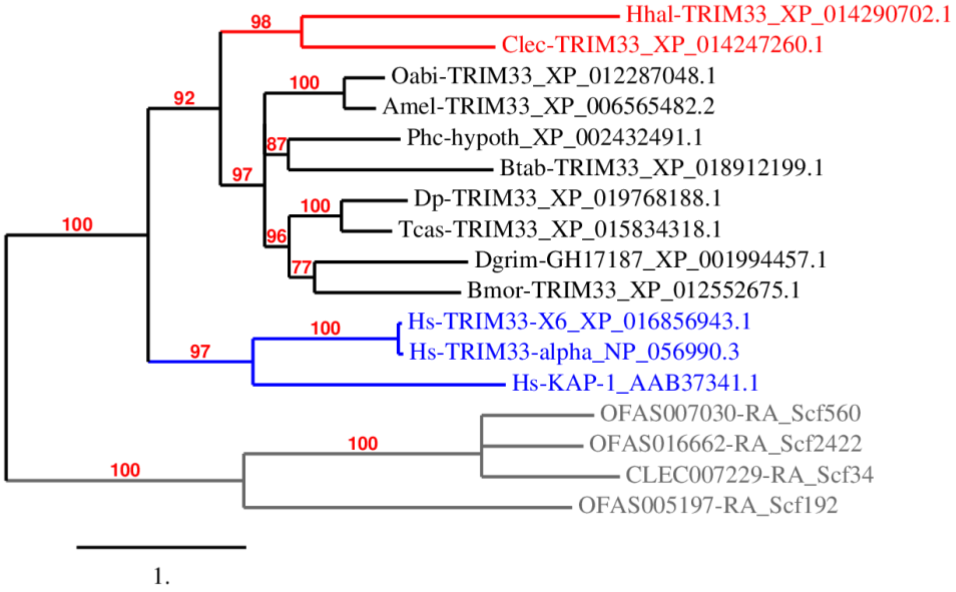

Figure S 5.26: Maximum likelihood phylogeny of selected tripartite motif-containing (TRIM) proteins. The TRIM28/33 family diversified after divergence of the vertebrate and insect lineages. The vertebrate TRIM28/33 clade (blue) is represented by the chromatin co-repressor KAP-1 (TRIM28) and two isoforms of TRIM33 from human. The diverged heteropteran TRIM28/33 homologs (red) form an outgroup to all other insect TRIM28/33 proteins, including that of the fellow hemipteran *Bemisia tabaci*. Genome tblastn searches with human KAP-1 find strongest homology among NHL repeat proteins of the TRIM71 and Brat subfamilies in *Oncopeltus* and *Cimex* (grey). GenBank accessions or OGS identifiers and scaffold locations ("Scf") are appended to protein labels. Species abbreviations: Vertebrata: Hs, *Homo sapiens*; Hemiptera: Btab, *Bemisia tabaci*; Clec, *Cimex lectularius*; Hhal, *Halyomorpha halys*; Ofas, *Oncopeltus fascia*tus; Psocodea: Phc, *Pediculus humanus corporis*; Hymenoptera: Amel, *Apis mellifera*; Oabi, *Orussus abietinus*; Coleoptera: Dp, *Dendroctonus ponderosae*; Tcas, *Tribolium castaneum*; Lepidoptera: Bmor, *Bombyx mori*; Diptera: Dgrim, *Drosophila grimshawi*. Branch support is given in percent, branch length scale is in substitutions per site.

6. Post-OGS v1.1 pipeline analyses

6.1 Protein gene orthology assessments via OrthoDB and BUSCO

Contributors: Panagiotis Ioannidis, Robert M. Waterhouse, Evgeny M. Zdobnov

Note that all of these analyses are based on the OGS v1.1, and only one isoform was considered per gene (n= 19,519 proteins). The methods described here are in support of main text Fig. 3a-b.

Orthology analysis

The OrthoDB resource [273] was used in order to find shared orthologs among *O. fasciatus* and the another ten arthropods (*Daphnia pulex*, *Zootermopsis nevadensis*, *Cimex lectularius*, *Rhodnius prolixus*, *Acyrthosiphon pisum*, *Pediculus humanus*, *Apis mellifera*, *Tribolium castaneum*, *Danaus plexippus*, and *Drosophila melanogaster*), using an expanded version of OrthoDB v.8 that also includes i5K species and mapped information for *Rhodnius prolixus.* Custom Perl scripts [274] were used in order to find the number of genes in each category shown in the bar chart in main text Fig. 3a. For the categories "Present in majority of species" and "Patchy distribution", we required that the given ortholog was found in 9-10 or 2-8 species, respectively. Proteins in the "Orthologs in other Arthropoda" category were found using the OrthoDB pipeline, while those in the "Homologs in other Arthropoda" did not meet those criteria but had hits with e-values <1e-05.

Furthermore, with the latest version of OrthoDB (v9.1, [275]), we specifically analyzed the 8,861 orthogroups resulting from orthology clustering analyses for the Hemiptera. Orthogroups are based on nine species that span the breadth of this order: *Acyrthosiphon pisum, Pachypsylla venusta, Homalodisca vitripennis, Diaphorina citri, Gerris buenoi, Rhodnius prolixus, Cimex lectularius, Halyomorpha halys*, and *Oncopeltus fasciatus.* Consistent with our BUSCO analyses presented below, we focused on how *Oncopeltus* compared to three of the other species, chosen for having high quality, published, and stable official gene sets: the pea aphid, *Cimex*, and *Rhodnius*. *Oncopeltus* compares favorably with the other species for both gene presence and for copy number (Table S 6.1). All four species have good orthogroup

representation (≥90%), and in fact *Oncopeltus* (94.0%) has better representation than either the pea aphid or *Rhodnius*. Furthermore, where orthologs are expected to be single copy, we only see a moderate increase in duplicates in *Oncopeltus* (1.64× compared to *Rhodnius*, which had the fewest), while the pea aphid – with known duplications – has a notable 5.6× higher level of duplications compared to *Rhodnius*.

Table S 6.1: OrthoDB v9.1 comparison of four species for presence and copy number in Hemiptera-level orthogroups.

**Hemipteran orthogroup metrics**	**Species**			
	Apis	***Rpro***	***Clec***	***Ofas***
A. Total hemipteran orthogroups that include this species (n= 8,861 orthogroups)	7,974 (90.0%)	8,261 (93.2%)	8,392 (94.7%)	8,330 (94.0%)
B. Total orthogroups from which the species is absent	887	600	469	531
B(i). # orthogroups missing an ortholog that is single-copy in the other 3 species	780	494	344	402
B(ii). # orthogroups missing an ortholog that is present (but not all single-copy) in the other 3 species	107	106	125	129
C. Number of groups with more than one ortholog but that is single-copy in the other 3 species	662	118	143	193
D. Total number of proteins in orthogroups with at least 3 of the 4 species	11,188	7,877	8,049	8,282

Phylogenetic analysis

For the phylogenetic analysis, an additional hemipteran species was included, the brown planthopper *Nilaparvata lugens*. However, since there was no available official gene set for this species, we extracted the sequences of conserved single-copy genes from the genome assembly using BUSCO [28] and mapped them onto OrthoDB. Subsequently, we used the genes that were present as single-copy in all twelve species in order to build a concatenated phylogenetic tree using RAxML (Randomized Axelerated Maximum Likelihood, [276]). Briefly, a multiple sequence alignment was performed using MUSCLE [277] for each orthologous group, separately. Then, the resulting alignments were trimmed using trimAl [206] with parameters "-w 3 -gt 0.95 -st 0.01". The trimmed alignments were concatenated using the "seqret" program from the EMBOSS suite [278]. This concatenated alignment was used to build the phylogeny using RAxML 7.6.6 with the PROTGAMMA model of amino acid substitutions and 100 bootstrap replicates.

BUSCO-based quality assessment

The quality of genome assemblies can be measured by searching for the presence of conserved genes. Moreover, if these conserved genes are also single-copy, the assembly can also be tested for unexpected duplications, which can be a sign of erroneous haplotype assembly. To this end, we used the Benchmarking Universal Single-Copy Orthologs (BUSCO, v3: [28, 29], scripts available under Gitlab project at https://gitlab.com/ezlab/busco), to measure the completeness of the milkweed bug genome as well as its set of predicted protein coding genes. We used both the Insecta and Arthropoda gene sets, which comprise genes that are present in at least 90% of the respective taxon. The values are highly similar between the two taxonomic datasets (Table S 6.2). This evaluation shows that most BUSCO genes are present in the *Oncopeltus* OGS v1.1 (only ~1% missing), although additional exons were identified on genomic scaffolds but not yet incorporated into the gene set (substantially fewer fragmented genes). These values compare favorably with that of high quality genomes that have been published for other hemipteran species, such as the bed bug and pea aphid (see main text).

Table S 6.2: BUSCO v3 statistics for gene count and percentage representation of the Insecta (n= 1658) and Arthropoda (n= 1066) datasets for the OGS v1.1 and Illumina assembly.

**Dataset**	**Complete (single copy and duplicated)**	**Duplicated (subset of "complete")**	**Fragmented**	**Missing**
OGS: Insecta	1,303 (78.6%)	41 (2.5%)	337 (20.3%)	18 (1.1%)
OGS: Arthropoda	870 (81.6%)	28 (2.6%)	190 (17.8%)	6 (0.6%)
Assembly: Insecta	1,568 (94.6%)	23 (1.4%)	50 (3.0%)	40 (2.4%)
Assembly: Arthropoda	1,018 (95.5%)	15 (1.4%)	29 (2.7%)	19 (1.8%)

6.2 Transcription factor classifications and orthology assignments

Contributor: Matthew T. Weirauch

See the main text for results and discussion on this section. The supplementary tables for this section are listed below.

Table S 6.3: Transcription factor counts. Values for heatmap representation (main text Fig.4a) of transcription factor abundance per family per species, log (base 2) scale, for 74 transcription factor families in 16 arthropod species (in Excel Supplement).

Table S 6.4: *Oncopeltus* transcription factors with orthology predictions based on their DNA-binding specificities, and their predicted DNA binding motifs (in Excel Supplement).

Table S 6.5: *Oncopeltus* transcription factors without orthology predictions. These *Oncopeltus* proteins could be automatically classified to a transcription factor family, but without a specific orthology assignment. Each protein's DNA-binding domains (DBDs) are listed sequentially, from 5' to 3' within the amino acid sequence (in Excel Supplement).

6.3 Gene structure evolution

Contributor: Kristen A. Panfilio

"Gold standard" manual curation gene set

To analyze gene structure, we created a high quality ("gold standard") dataset of 30 genes whose manual curation could reasonably ensure complete and accurate protein coding gene models across seven species from four insect orders (see the main text for results and discussion on this section: main text Fig. 6a). We deliberately chose multi-exonic genes encoding fairly large proteins (median of 1720 aa), with clear single-copy orthology and strong RNA-seq expression to support model curation. For broad molecular sampling, genes were chosen that encode proteins of diverse functional classes, ranging from structural molecules to signaling pathway components, enzymes, and intracellular regulators of organelle structure and vesicular trafficking (detailed in Table S 6.6).

Splice site analysis

We annotated intron positions within multiple sequence alignments of orthologous proteins and plotted gains and losses onto a phylogeny for the genes *hemocytin* (also known as *Hemolectin*, *Hml*), *Tenascin major* (*Ten-m*), and *UDP-galactose 4′-epimerase* (*GalE*) (see the main text for results and discussion on this section: main text Fig. 7; details on aligned splice positions' conservation is given in supplementary Tables S 6.7-6.9). The many-exon genes were taken from our initial "gold standard" dataset. The gene *hemocytin* (also known as *Hemolectin*, *Hml*) encodes a hemocyte clotting agent with numerous functional domains for cuticle- and protein-protein binding, and it had the most exons of any gene in the dataset (≤74 exons). Unfortunately, it was not possible to annotate a complete model of this gene in the thrips, which as a thysanopteran represents a closely related outgroup to the Hemiptera, and therefore we additionally evaluated *Tenascin major* (*Ten-m*), which encodes a teneurin family multi-domain protein involved in neural development and synaptic transmission. At the other end of the spectrum, we chose an ancient gene with conservation extending to bacteria, such that the ancestral protein had no introns: *UDP-galactose 4′-epimerase* (*GalE*) encodes an enzyme for sugar metabolism (EC

5.1.3.2) and is found across virtually all kingdoms of life. In fact, sequence conservation is so high at the nucleotide level that this gene initially came up as a potential candidate in our pipeline analyses of lateral gene transfer events in *Oncopeltus* (see also Supplemental Note 2.2), although we find here that the ancestral insect *epimerase* gene already had at least three exons.

Methods

Gene models: Single-copy orthologous gene models were obtained from public genome browsers (*D. melanogaster* and *T. castaneum*) or manually curated from automatic predictions and available expression data evidence tracks in the i5K NAL Apollo web instances (all other species: *O. fasciatus, C. lectularius, G. buenoi, F. occidentalis, A. glabripennis, C. capitata*). Manual curation was performed iteratively and by orthology comparisons in sequence alignments, to ensure protein sequence completeness even when genes were split across draft assembly scaffolds. In the case of *hemocytin* and *furry*, the models in *O. fasciatus* and *G. buenoi* were filled with place-holder X's where strong orthology conservation (including empirical data from the other two hemipterans) made clear that small internal exons were truncated or absent in the current assembly, involving the inference of two splice positions in *G. buenoi* and three in *O. fasciatus*.

Alignment: Protein translations were obtained for each exon, and the splice positions were marked (rounded to the nearest triplet/ whole amino acid position). A protein sequence alignment into which splice positions were encoded (denoted by the character "X") was then generated with ClustalW (accessed at http://www.genome.jp/tools/clustalw/) and manually refined.

Inference of evolutionary patterns: All splice positions were considered individually, and only those for which the sequence alignment was particularly poor were excluded (six sites within the less conserved N-terminal region and two other sites within *hemocytin*). Evolutionary patterns of splice position gain and loss were encoded as a binary presence/absence value per position for each species (Tables S 6.7-6.9), and the most parsimonious inference (fewest lineage-specific changes) to generate this pattern was assumed, with no weighting of probability for gains relative to losses.

Table S 6.6 List of “gold standard” manually curated genes evaluated for gene structure properties, including public database IDs for *T. castaneum* and *D. melanogaster* orthologs (in Excel Supplement).

Table S 6.7: Hemocytin (Hemolectin, Hml) splice site conservation analysis (7 species) (in Excel Supplement).

Table S 6.8: Tenascin major (Ten-m) splice site conservation analysis (8 species) (in Excel Supplement).

Table S 6.9: UDP-galactose 4′ -epimerase (GalE) splice site conservation analysis (8 species) (in Excel Supplement).

6.4 Interspecific comparisons of metabolic enzymes

Contributors: Patrice Baa-Puyoule, Gérard Febvay, Nicolas Parisot, Stefano Colella

See the main text for results and discussion on this section. The supplementary tables for this section are listed below.

Table S 6.10: Global EC statistics. Global comparison of Enzyme Commission (EC) categories and amino acid metabolism enzymes (KEGG) present in 28 samples for 27 species in the ArthropodaCyc repository, including the newly generated OncfaCyc database for *Oncopeltus* (in Excel Supplement).

Table S 6.11: Detailed listing of EC categories that are uniquely present or absent from *Oncopeltus* (in Excel Supplement).

Table S 6.12: Detailed comparison tables and Venn diagram of amino acid metabolism enzyme repertoires across four hemipterans (in Excel Supplement).

Table S 6.13: Urea cycle comparison across 27 insect species. Presence/absence of EC numbers essential to the urea cycle are indicated for each species (in Excel Supplement).

7. References

1. Gnerre S, Maccallum I, Przybylski D, Ribeiro FJ, Burton JN, Walker BJ, Sharpe T, Hall G, Shea TP, Sykes S, et al: **High-quality draft assemblies of mammalian genomes from massively parallel sequence data.** *Proc Natl Acad Sci U S A* 2011, **108:**1513-1518.
2. Hare EE, Johnston JS: **Genome size determination using flow cytometry of propidium iodide-stained nuclei.** *Methods Mol Biol* 2011, **772:**3-12.
3. Messthaler H, Traut W: **Phases of Sex Chromosome Inactivation in *Oncopeltus fasciatus* and *Pyrrhocoris apterus* (Insecta, Heteroptera).** *Caryologia* 1975, **28:**501-510.
4. Marcais G, Kingsford C: **A fast, lock-free approach for efficient parallel counting of occurrences of k-mers.** *Bioinformatics* 2011, **27:**764-770.
5. Bushnell B: **BBMap short read aligner.** 2016.
6. Chen W, Hasegawa DK, Arumuganathan K, Simmons AM, Wintermantel WM, Fei Z, Ling KS: **Estimation of the Whitefly *Bemisia tabaci* Genome Size Based on k-mer and Flow Cytometric Analyses.** *Insects* 2015, **6:**704-715.
7. Guo LT, Wang SL, Wu QJ, Zhou XG, Xie W, Zhang YJ: **Flow cytometry and K-mer analysis estimates of the genome sizes of *Bemisia tabaci* B and Q (Hemiptera: Aleyrodidae).** *Front Physiol* 2015, **6:**144.
8. Derks MF, Smit S, Salis L, Schijlen E, Bossers A, Mateman C, Pijl AS, de Ridder D, Groenen MA, Visser ME, Megens HJ: **The Genome of Winter Moth (*Operophtera brumata*) Provides a Genomic Perspective on Sexual Dimorphism and Phenology.** *Genome Biol Evol* 2015, **7:**2321-2332.
9. **K-mer Spectrum Primer** [http://www.broadinstitute.org/software/allpaths-lg/blog/?page_id=693]
10. Liu B, Shi Y, Yuan J, Hu X, Zhang H, Li N, Li Z, Chen Y, Mu D, Fan W: **Estimation of genomic characteristics by analyzing k-mer frequency in de novo genome projects.** http://arxivorg/ 2013, **1308**.
11. Shan G, Zheng W-M: **An ℓ-mer component distribution for genome size esimation.** 2009.
12. Chor B, Horn D, Goldman N, Levy Y, Massingham T: **Genomic DNA k-mer spectra: models and modalities.** *Genome Biol* 2009, **10:**R108.
13. Schmidt NO: **On the *k*-mer frequency spectra of organism genome and proteome sequences with a preliminary machine learning assessment of prime predictability.** BOISE STATE UNIVERSITY GRADUATE COLLEGE, 2012.
14. Li F, Fan G, Wang K, Sun F, Yuan Y, Song G, Li Q, Ma Z, Lu C, Zou C, et al: **Genome sequence of the cultivated cotton *Gossypium arboreum*.** *Nat Genet* 2014, **46:**567-572.

15. Abe H, Yoshikawa N, Sarower MG, Okada S: **Physiological function and metabolism of free D-alanine in aquatic animals.** *Biol Pharm Bull* 2005, **28:**1571-1577.
16. Schroder R, Wegrzyn TF, Sharma NN, Atkinson RG: **LeMAN4 endo-beta-mannanase from ripe tomato fruit can act as a mannan transglycosylase or hydrolase.** *Planta* 2006, **224:**1091-1102.
17. Nikoh N, Nakabachi A: **Aphids acquired symbiotic genes via lateral gene transfer.** *BMC Biol* 2009, **7:**12.
18. Wheeler D, Redding AJ, Werren JH: **Characterization of an ancient lepidopteran lateral gene transfer.** *PLoS One* 2012, **8:**e59262.
19. Benoit JB, Adelman ZN, Reinhardt K, Dolan A, Poelchau M, Jennings EC, Szuter EM, Hagan RW, Gujar H, Shukla JN, et al: **Unique features of a global human ectoparasite identified through sequencing of the bed bug genome.** *Nat Commun* 2016, **7:**10165.
20. Duncan EJ, Wilson MJ, Smith JM, Dearden PK: **Evolutionary origin and genomic organisation of runt-domain containing genes in arthropods.** *BMC Genomics* 2008, **9:**558.
21. Bao Z, Eddy SR: **Automated de novo identification of repeat sequence families in sequenced genomes.** *Genome Res* 2002, **12:**1269-1276.
22. Price AL, Jones NC, Pevzner PA: **De novo identification of repeat families in large genomes.** *Bioinformatics* 2005, **21 Suppl 1:**i351-358.
23. Benson G: **Tandem repeats finder: a program to analyze DNA sequences.** *Nucleic Acids Res* 1999, **27:**573-580.
24. Ruiz-Ruano FJ, López-León MD, Cabrero J, Camacho JPM: **High-throughput analysis of the satellitome illuminates satellite DNA evolution.** *Scientific Reports* 2016, **6:**28333.
25. Ross MG, Russ C, Costello M, Hollinger A, Lennon NJ, Hegarty R, Nusbaum C, Jaffe DB: **Characterizing and measuring bias in sequence data.** *Genome Biol* 2013, **14:**R51.
26. English AC, Richards S, Han Y, Wang M, Vee V, Qu J, Qin X, Muzny DM, Reid JG, Worley KC, Gibbs RA: **Mind the gap: upgrading genomes with Pacific Biosciences RS long-read sequencing technology.** *PLoS One* 2012, **7:**e47768.
27. Wang X, Fang X, Yang P, Jiang X, Jiang F, Zhao D, Li B, Cui F, Wei J, Ma C, et al: **The locust genome provides insight into swarm formation and long-distance flight.** *Nat Commun* 2014, **5:**2957.
28. Simão FA, Waterhouse RM, Ioannidis P, Kriventseva EV, Zdobnov EM: **BUSCO: assessing genome assembly and annotation completeness with single-copy orthologs.** *Bioinformatics* 2015, **31:**3210-3212.

29. Waterhouse RM, Seppey M, Simao FA, Manni M, Ioannidis P, Klioutchnikov G, Kriventseva EV, Zdobnov EM: **BUSCO applications from quality assessments to gene prediction and phylogenomics.** *Mol Biol Evol* 2017.

30. Xue J, Zhou X, Zhang C-X, Yu L-L, Fan H-W, Wang Z, Xu H-J, Xi Y, Zhu Z-R, Zhou W-W, et al: **Genomes of the rice pest brown planthopper and its endosymbionts reveal complex complementary contributions for host adaptation.** *Genome Biol* 2014, **15:**521.

31. Platt RN, 2nd, Blanco-Berdugo L, Ray DA: **Accurate transposable element annotation is vital when analyzing new genome assemblies.** *Genome Biol Evol* 2016.

32. The International Aphid Genomics Consortium: **Genome sequence of the pea aphid *Acyrthosiphon pisum*** *PLoS Biol* 2010, **8:**e1000313.

33. Zhen Y, Aardema ML, Medina EM, Schumer M, Andolfatto P: **Parallel molecular evolution in an herbivore community.** *Science* 2012, **337:**1634-1637.

34. Schoville SD, Chen YH, Andersson MN, Benoit JB, Bhandari A, Bowsher JH, Brevik K, Cappelle K, Chen MM, Childers AK, et al: **A model species for agricultural pest genomics: the genome of the Colorado potato beetle, *Leptinotarsa decemlineata* (Coleoptera: Chrysomelidae).** *Sci Rep* 2018, **8:**1931.

35. Scolari F, Benoit JB, Michalkova V, Aksoy E, Takac P, Abd-Alla AM, Malacrida AR, Aksoy S, Attardo GM: **The spermatophore in *Glossina morsitans morsitans*: Insights into male contributions to reproduction.** *Sci Rep* 2016, **6:**20334.

36. Holt C, Yandell M: **MAKER2: an annotation pipeline and genome-database management tool for second-generation genome projects.** *BMC Bioinformatics* 2011, **12:**491.

37. Stanke M, Diekhans M, Baertsch R, Haussler D: **Using native and syntenically mapped cDNA alignments to improve de novo gene finding.** *Bioinformatics* 2008, **24:**637-644.

38. Korf I: **Gene finding in novel genomes.** *BMC Bioinformatics* 2004, **5:**59.

39. Murali SC, The i5k genome assembly team (29 additional authors), Han Y, Richards S, Worley K, Muzny D, Gibbs R, Koelzer S, Panfilio KA: ***Oncopeltus fasciatus* genome assembly 1.0.** *Ag Data Commons (Database)* 2015**:**http://dx.doi.org/10.15482/USDA.ADC/1173238.

40. Hughes DST, Koelzer S, Panfilio KA, Richards S: ***Oncopeltus fasciatus* genome annotations v0.5.3.** *Ag Data Commons (Database)* 2015**:**http://dx.doi.org/10.15482/USDA.ADC/1173237.

41. Vargas Jentzsch IM, Hughes DST, Poelchau M, Robertson HM, Benoit JB, Rosendale AJ, Armisén D, Duncan EJ, Vreede BMI, Jacobs CGC, et al: ***Oncopeltus fasciatus* Official Gene Set OGS_v1.1 for genome assembly Oncopeltus fasciatus v1.0.** *Ag Data Commons (Database)* 2015**:**http://dx.doi.org/10.15482/USDA.ADC/1173142.

42. Dereeper A, Audic S, Claverie JM, Blanc G: **BLAST-EXPLORER helps you building datasets for phylogenetic analysis.** *BMC Evol Biol* 2010, **10:**8.

43. Dereeper A, Guignon V, Blanc G, Audic S, Buffet S, Chevenet F, Dufayard JF, Guindon S, Lefort V, Lescot M, et al: **Phylogeny.fr: robust phylogenetic analysis for the non-specialist.** *Nucleic Acids Res* 2008, **36:**W465-469.

44. Denton JF, Lugo-Martinez J, Tucker AE, Schrider DR, Warren WC, Hahn MW: **Extensive error in the number of genes inferred from draft genome assemblies.** *PLoS Comput Biol* 2014, **10:**e1003998.

45. Megy K, Emrich SJ, Lawson D, Campbell D, Dialynas E, Hughes DS, Koscielny G, Louis C, Maccallum RM, Redmond SN, et al: **VectorBase: improvements to a bioinformatics resource for invertebrate vector genomics.** *Nucleic Acids Res* 2012, **40:**D729-734.

46. McKenna DD, Scully ED, Pauchet Y, Hoover K, Kirsch R, Geib SM, Mitchell RF, Waterhouse RM, Ahn SJ, Arsala D, et al: **Genome of the Asian longhorned beetle (*Anoplophora glabripennis*), a globally significant invasive species, reveals key functional and evolutionary innovations at the beetle-plant interface.** *Genome Biol* 2016, **17:**227.

47. Lynch JA: **Diversity of molecules and mechanisms in establishing insect anteriorâ€“posterior polarity.** *Current Opinion in Insect Science* 2014, **1:**39-44.

48. Ewen-Campen B, Shaner N, Panfilio KA, Suzuki Y, Roth S, Extavour CG: **The maternal and early embryonic transcriptome of the milkweed bug *Oncopeltus fasciatus*.** *BMC Genomics* 2011, **12:**61.

49. Weisbrod A, Cohen M, Chipman AD: **Evolution of the insect terminal patterning system--insights from the milkweed bug, *Oncopeltus fasciatus*.** *Dev Biol* 2013, **380:**125-131.

50. Duncan EJ, Benton MA, Dearden PK: **Canonical terminal patterning is an evolutionary novelty.** *Dev Biol* 2013, **377:**245-261.

51. Ben-David J, Chipman AD: **Mutual regulatory interactions of the trunk gap genes during blastoderm patterning in the hemipteran *Oncopeltus fasciatus*.** *Dev Biol* 2010, **346:**140-149.

52. Ben-David J, Chipman AD: **Mutual regulatory interactions of the trunk gap genes during blastoderm patterning in the hemipteran *Oncopeltus fasciatus*.** *Dev Biol* 2010, **346:**140-149.

53. Erezyilmaz DF, Kelstrup HC, Riddiford LM: **The nuclear receptor E75A has a novel pair-rule-like function in patterning the milkweed bug, *Oncopeltus fasciatus*.** *Dev Biol* 2009, **334:**300-310.

54. Liu PZ, Kaufman TC: ***hunchback* is required for suppression of abdominal identity, and for proper germband growth and segmentation in the intermediate germband insect *Oncopeltus fasciatus*.** *Development* 2004, **131:**1515-1527.

55. Peel AD, Telford MJ, Akam M: **The evolution of hexapod engrailed-family genes: evidence for conservation and concerted evolution.** *Proc Biol Sci* 2006, **273:**1733-1742.

56. Krumlauf R: **Evolution of the vertebrate Hox homeobox genes.** *Bioessays* 1992, **14:**245-252.

57. Richards S, Gibbs RA, Weinstock GM, Brown SJ, Denell R, Beeman RW, Gibbs R, Bucher G, Friedrich M, Grimmelikhuijzen CJ, et al: **The genome of the model beetle and pest Tribolium castaneum.** *Nature* 2008, **452:**949-955.

58. **The homebox page** [http://evolution.genetics.washington.edu/phylip.html]

59. Gómez-Skarmeta J-L, Corral RDd, Calle-Mustienes Edl, Ferrés-Marcó D, Modolell J: ***araucan* and *caupolican*, two members of the novel Iroquois Complex, encode homeoproteins that control proneural and vein-forming genes.** *Cell* 1996, **85:**95-105.

60. Gomez-Skarmeta JL, Modolell J: **Iroquois genes: genomic organization and function in vertebrate neural development.** *Curr Opin Genet Dev* 2002, **12:**403-408.

61. Irimia M, Maeso I, Garcia-Fernandez J: **Convergent evolution of clustering of Iroquois homeobox genes across metazoans.** *Mol Biol Evol* 2008, **25:**1521-1525.

62. Cavodeassi F, Modolell J, Gómez-Skarmeta JL: **The Iroquois family of genes: from body building to neural patterning.** *Development* 2001, **128:**2847-2855.

63. Kerner P, Ikmi A, Coen D, Vervoort M: **Evolutionary history of the iroquois/Irx genes in metazoans.** *BMC Evol Biol* 2009, **9:**74.

64. Maeso I, Irimia M, Tena JJ, González-Pérez E, Tran D, Ravi V, Venkatesh B, Campuzano S, Gómez-Skarmeta JL, Garcia-Fernàndez J: **An ancient genomic regulatory block conserved across bilaterians and its dismantling in tetrapods by retrogene replacement.** *Genome Res* 2012, **22:**642-655.

65. Savard J, Tautz D, Richards S, Weinstock GM, Gibbs RA, Werren JH, Tettelin H, Lercher MJ: **Phylogenomic analysis reveals bees and wasps (Hymenoptera) at the base of the radiation of holometabolous insects.** *Genome Res* 2006, **16:**1334-1338.

66. Tamura K, Stecher G, Peterson D, Filipski A, Kumar S: **MEGA6: Molecular Evolutionary Genetics Analysis version 6.0.** *Mol Biol Evol* 2013, **30:**2725-2729.

67. Reim I, Lee HH, Frasch M: **The T-box-encoding Dorsocross genes function in amnioserosa development and the patterning of the dorsolateral germ band downstream of Dpp.** *Development* 2003, **130:**3187-3204.

68. Reim I, Frasch M: **The Dorsocross T-box genes are key components of the regulatory network controlling early cardiogenesis in Drosophila.** *Development* 2005, **132:**4911-4925.
69. Horn T, Panfilio KA: **Novel functions for Dorsocross in epithelial morphogenesis in the beetle Tribolium castaneum.** *Development* 2016, **143:**3002-3011.
70. Mangelsdorf DJ, Thummel C, Beato M, Herrlich P, Schutz G, Umesono K, Blumberg B, Kastner P, Mark M, Chambon P, Evans RM: **The nuclear receptor superfamily: the second decade.** *Cell* 1995, **83:**835-839.
71. Chambon P: **The nuclear receptor superfamily: a personal retrospect on the first two decades.** *Mol Endocrinol* 2005, **19:**1418-1428.
72. Margolis RN, Evans RM, O'Malley BW: **The Nuclear Receptor Signaling Atlas: development of a functional atlas of nuclear receptors.** *Mol Endocrinol* 2005, **19:**2433-2436.
73. Taneja R: *Nuclear Receptors in Development.* Oxford: Elsevier; 2006.
74. Fahrbach SE, Smagghe G, Velarde RA: **Insect nuclear receptors.** *Annual review of entomology* 2012, **57:**83-106.
75. McKenna NJ, Cooney AJ, DeMayo FJ, Downes M, Glass CK, Lanz RB, Lazar MA, Mangelsdorf DJ, Moore DD, Qin J, et al: **Minireview: Evolution of NURSA, the Nuclear Receptor Signaling Atlas.** *Mol Endocrinol* 2009, **23:**740-746.
76. Xiao X, Wang P, Chou KC: **Recent progresses in identifying nuclear receptors and their families.** *Current topics in medicinal chemistry* 2013, **13:**1192-1200.
77. Laudet V: **Evolution of the nuclear receptor superfamily: early diversification from an ancestral orphan receptor.** *J Mol Endocrinol* 1997, **19:**207-226.
78. Markov GV, Laudet V: **Origin and evolution of the ligand-binding ability of nuclear receptors.** *Molecular and cellular endocrinology* 2011, **334:**21-30.
79. Sladek FM: **What are nuclear receptor ligands?** *Molecular and cellular endocrinology* 2011, **334:**3-13.
80. The Nuclear Receptor Nomenclature Committee: **A unified nomenclature for the nuclear receptor superfamily.** *Cell* 1999, **97:**161-163.
81. Glass CK, Rosenfeld MG: **The coregulator exchange in transcriptional functions of nuclear receptors.** *Genes Dev* 2000, **14:**121-141.
82. Tremblay JJ, Marcil A, Gauthier Y, Drouin J: **Ptx1 regulates SF-1 activity by an interaction that mimics the role of the ligand-binding domain.** *EMBO J* 1999, **18:**3431-3441.
83. Yoo J, Ko S, Kim H, Sampson H, Yun JH, Choe KM, Chang I, Arrowsmith CH, Krause HM, Cho HS, Lee W: **Crystal structure of Fushi tarazu factor 1 ligand binding domain/Fushi**

tarazu peptide complex identifies new class of nuclear receptors. *The Journal of biological chemistry* 2011, **286:**31225-31231.

84. King-Jones K, Thummel CS: **Nuclear receptors--a perspective from *Drosophila*.** *Nat Rev Genet* 2005, **6:**311-323.

85. Ruaud AF, Lam G, Thummel CS: **The Drosophila NR4A nuclear receptor DHR38 regulates carbohydrate metabolism and glycogen storage.** *Molecular endocrinology* 2011, **25:**83-91.

86. Tennessen JM, Baker KD, Lam G, Evans J, Thummel CS: **The Drosophila estrogen-related receptor directs a metabolic switch that supports developmental growth.** *Cell Metab* 2011, **13:**139-148.

87. Shigenobu S, Bickel RD, Brisson JA, Butts T, Chang CC, Christiaens O, Davis GK, Duncan EJ, Ferrier DE, Iga M, et al: **Comprehensive survey of developmental genes in the pea aphid, *Acyrthosiphon pisum*: frequent lineage-specific duplications and losses of developmental genes.** *Insect Mol Biol* 2010, **19 Suppl 2:**47-62.

88. St Pierre SE, Ponting L, Stefancsik R, McQuilton P, FlyBase C: **FlyBase 102--advanced approaches to interrogating FlyBase.** *Nucleic Acids Res* 2014, **42:**D780-788.

89. Parker L, Stathakis DG, Arora K: **Regulation of BMP and activin signaling in Drosophila.** *Prog Mol Subcell Biol* 2004, **34:**73-101.

90. Sachs L, Chen YT, Drechsler A, Lynch JA, Panfilio KA, Lassig M, Berg J, Roth S: **Dynamic BMP signaling polarized by Toll patterns the dorsoventral axis in a hemimetabolous insect.** *eLife* 2015, **4:**e05502.

91. Weiss A, Charbonnier E, Ellertsdottir E, Tsirigos A, Wolf C, Schuh R, Pyrowolakis G, Affolter M: **A conserved activation element in BMP signaling during Drosophila development.** *Nat Struct Mol Biol* 2010, **17:**69-76.

92. Balemans W, Van Hul W: **Extracellular regulation of BMP signaling in vertebrates: a cocktail of modulators.** *Dev Biol* 2002, **250:**231-250.

93. Ozuak O, Buchta T, Roth S, Lynch JA: **Ancient and diverged TGF-beta signaling components in Nasonia vitripennis.** *Dev Genes Evol* 2014, **224:**223-233.

94. van der Zee M, da Fonseca RN, Roth S: **TGF-beta signaling in *Tribolium*: vertebrate-like components in a beetle.** *Dev Genes Evol* 2008, **218:**203-213.

95. Podos SD, Hanson KK, Wang YC, Ferguson EL: **The DSmurf ubiquitin-protein ligase restricts BMP signaling spatially and temporally during Drosophila embryogenesis.** *Dev Cell* 2001, **1:**567-578.

96. Xia L, Jia S, Huang S, Wang H, Zhu Y, Mu Y, Kan L, Zheng W, Wu D, Li X, et al: **The Fused/Smurf complex controls the fate of Drosophila germline stem cells by generating a gradient BMP response.** *Cell* 2010, **143:**978-990.

97. Norman M, Vuilleumier R, Springhorn A, Gawlik J, Pyrowolakis G: **Pentagone internalises glypicans to fine-tune multiple signalling pathways.** *Elife* 2016, **5**.

98. Lynch JA, Roth S: **The evolution of dorsal-ventral patterning mechanisms in insects.** *Genes Dev* 2011, **25:**107-118.

99. Müller U, Vogel P, Alber G, Schaub GA: **The innate immune system of mammals and insects.** *Contrib Microbiol* 2008, **15:**21-44.

100. Stein DS, Stevens LM: **Maternal control of the Drosophila dorsal-ventral body axis.** *Wiley Interdiscip Rev Dev Biol* 2014, **3:**301-330.

101. Benton MA, Pechmann M, Frey N, Stappert D, Conrads KH, Chen YT, Stamataki E, Pavlopoulos A, Roth S: **Toll Genes Have an Ancestral Role in Axis Elongation.** *Curr Biol* 2016, **26:**1609-1615.

102. Leulier F, Lemaitre B: **Toll-like receptors--taking an evolutionary approach.** *Nat Rev Genet* 2008, **9:**165-178.

103. Towb P, Sun H, Wasserman SA: **Tube Is an IRAK-4 homolog in a Toll pathway adapted for development and immunity.** *J Innate Immun* 2009, **1:**309-321.

104. Kumar S, Stecher G, Tamura K: **MEGA7: Molecular Evolutionary Genetics Analysis Version 7.0 for Bigger Datasets.** *Mol Biol Evol* 2016, **33:**1870-1874.

105. Reeves GT, Stathopoulos A: **Graded dorsal and differential gene regulation in the Drosophila embryo.** *Cold Spring Harb Perspect Biol* 2009, **1:**a000836.

106. Cremazy F, Berta P, Girard F: **Sox neuro, a new *Drosophila* Sox gene expressed in the developing central nervous system.** *Mech Dev* 2000, **93:**215-219.

107. Skeath JB: **At the nexus between pattern formation and cell-type specification: the generation of individual neuroblast fates in the Drosophila embryonic central nervous system.** *Bioessays* 1999, **21:**922-931.

108. Schneider M, Dorn A: **Differential infectivity of two Pseudomonas species and the immune response in the milkweed bug, Oncopeltus fasciatus (Insecta: Hemiptera).** *J Invertebr Pathol* 2001, **78:**135-140.

109. Bao YY, Qu LY, Zhao D, Chen LB, Jin HY, Xu LM, Cheng JA, Zhang CX: **The genome- and transcriptome-wide analysis of innate immunity in the brown planthopper, Nilaparvata lugens.** *BMC Genomics* 2013, **14:**160.

110. Talavera G, Vila R: **What is the phylogenetic signal limit from mitogenomes? The reconciliation between mitochondrial and nuclear data in the Insecta class phylogeny.** *BMC Evol Biol* 2011, **11:**315.

111. Leone P, Bischoff V, Kellenberger C, Hetru C, Royet J, Roussel A: **Crystal structure of *Drosophila* PGRP-SD suggests binding to DAP-type but not lysine-type peptidoglycan.** *Mol Immunol* 2008, **45:**2521-2530.

112. Leulier F, Parquet C, Pili-Floury S, Ryu JH, Caroff M, Lee WJ, Mengin-Lecreulx D, Lemaitre B: **The *Drosophila* immune system detects bacteria through specific peptidoglycan recognition.** *Nat Immunol* 2003, **4:**478-484.

113. Yokoi K, Koyama H, Ito W, Minakuchi C, Tanaka T, Miura K: **Involvement of NF-kappaB transcription factors in antimicrobial peptide gene induction in the red flour beetle, Tribolium castaneum.** *Dev Comp Immunol* 2012, **38:**342-351.

114. Yokoi K, Koyama H, Minakuchi C, Tanaka T, Miura K: **Antimicrobial peptide gene induction, involvement of Toll and IMD pathways and defense against bacteria in the red flour beetle, Tribolium castaneum.** *Results Immunol* 2012, **2:**72-82.

115. Duncan EJ, Dearden PK: **Evolution of a genomic regulatory domain: the role of gene co-option and gene duplication in the Enhancer of split complex.** *Genome Res* 2010, **20:**917-928.

116. Schaeper ND, Prpic NM, Wimmer EA: **A clustered set of three Sp-family genes is ancestral in the Metazoa: evidence from sequence analysis, protein domain structure, developmental expression patterns and chromosomal location.** *BMC Evol Biol* 2010, **10:**88.

117. Birkan M, Schaeper ND, Chipman AD: **Early patterning and blastodermal fate map of the head in the milkweed bug Oncopeltus fasciatus.** *Evol Dev* 2011, **13:**436-447.

118. Thompson JD, Higgins DG, Gibson TJ: **CLUSTAL W: improving the sensitivity of progressive multiple sequence alignment through sequence weighting, position-specific gap penalties and weight matrix choice.** *Nucleic Acids Res* 1994, **22:**4673-4680.

119. Murat S, Hopfen C, McGregor AP: **The function and evolution of Wnt genes in arthropods.** *Arthropod Struct Dev* 2010, **39:**446-452.

120. Oberhofer G, Grossmann D, Siemanowski JL, Beissbarth T, Bucher G: **Wnt/beta-catenin signaling integrates patterning and metabolism of the insect growth zone.** *Development* 2014, **141:**4740-4750.

121. Bao R, Fischer T, Bolognesi R, Brown SJ, Friedrich M: **Parallel duplication and partial subfunctionalization of beta-catenin/armadillo during insect evolution.** *Mol Biol Evol* 2012, **29:**647-662.

122. Janssen R, Le Gouar M, Pechmann M, Poulin F, Bolognesi R, Schwager EE, Hopfen C, Colbourne JK, Budd GE, Brown SJ, et al: **Conservation, loss, and redeployment of Wnt ligands in protostomes: implications for understanding the evolution of segment formation.** *BMC Evol Biol* 2010, **10:**374.

123. Bolognesi R, Farzana L, Fischer TD, Brown SJ: **Multiple Wnt genes are required for segmentation in the short-germ embryo of Tribolium castaneum.** *Curr Biol* 2008, **18:**1624-1629.

124. Sullivan JC, Ryan JF, Mullikin JC, Finnerty JR: **Conserved and novel Wnt clusters in the basal eumetazoan Nematostella vectensis.** *Dev Genes Evol* 2007, **217:**235-239.

125. Doumpas N, Jekely G, Teleman AA: **Wnt6 is required for maxillary palp formation in Drosophila.** *BMC Biol* 2015, **11:**104.

126. Beermann A, Pruhs R, Lutz R, Schroder R: **A context-dependent combination of Wnt receptors controls axis elongation and leg development in a short germ insect.** *Development* 2011, **138:**2793-2805.

127. Kojima T: **The mechanism of *Drosophila* leg development along the proximodistal axis.** *Dev Growth Differ* 2004, **46:**115-129.

128. Siegel JG, Fristrom JW: **The biochemistry of imaginal disc development. In (eds.). The Genetics and Biology of Drosophila.** In *Volume* 2A. Edited by Ashburner M, Wright TRF. New York: Academic Press; 1978: 317–394

129. Cohen B, McGuffin ME, Pfeifle C, Segal D, Cohen SM: **apterous, a gene required for imaginal disc development in Drosophila encodes a member of the LIM family of developmental regulatory proteins.** *Genes Dev* 1992, **6:**715-729.

130. Simcox AA, Roberts IJ, Hersperger E, Gribbin MC, Shearn A, Whittle JR: **Imaginal discs can be recovered from cultured embryos mutant for the segment-polarity genes engrailed, naked and patched but not from wingless.** *Development* 1989, **107:**715-722.

131. Ober KA, Jockusch EL: **The roles of wingless and decapentaplegic in axis and appendage development in the red flour beetle, Tribolium castaneum.** *Dev Biol* 2006, **294:**391-405.

132. Angelini DR, Kaufman TC: **Functional analyses in the milkweed bug *Oncopeltus fasciatus* (Hemiptera) support a role for Wnt signaling in body segmentation but not appendage development.** *Dev Biol* 2005, **283:**409-423.

133. Jiang J, Struhl G: **Complementary and mutually exclusive activities of decapentaplegic and wingless organize axial patterning during Drosophila leg development.** *Cell* 1996, **86:**401-409.

134. Abu-Shaar M, Mann RS: **Generation of multiple antagonistic domains along the proximodistal axis during Drosophila leg development.** *Development* 1998, **125:**3821-3830.

135. Cohen SM, Bronner G, Kuttner F, Jurgens G, Jackle H: **Distal-less encodes a homoeodomain protein required for limb development in Drosophila.** *Nature* 1989, **338:**432-434.

136. Wu J, Cohen SM: **Proximodistal axis formation in the *Drosophila* leg: subdivision into proximal and distal domains by *Homothorax* and *Distal-less*.** *Development* 1999, **126:**109-117.

137. Rieckhof GE, Casares F, Ryoo HD, Abu-Shaar M, Mann RS: **Nuclear translocation of extradenticle requires homothorax, which encodes an extradenticle-related homeodomain protein.** *Cell* 1997, **91:**171-183.

138. Angelini DR, Kaufman TC: **Functional analyses in the hemipteran *Oncopeltus fasciatus* reveal conserved and derived aspects of appendage patterning in insects.** *Dev Biol* 2004, **271:**306-321.

139. Angelini DR, Kikuchi M, Jockusch EL: **Genetic patterning in the adult capitate antenna of the beetle *Tribolium castaneum*.** *Dev Biol* 2009, **327:**240-251.

140. Angelini DR, Smith FW, Aspiras AC, Kikuchi M, Jockusch EL: **Patterning of the adult mandibulate mouthparts in the red flour beetle, *Tribolium castaneum*.** *Genetics* 2012, **190:**639-654.

141. Halder G, Polaczyk P, Kraus ME, Hudson A, Kim J, Laughon A, Carroll S: **The Vestigial and Scalloped proteins act together to directly regulate wing-specific gene expression in Drosophila.** *Genes Dev* 1998, **12:**3900-3909.

142. Williams JA, Bell JB, Carroll SB: **Control of Drosophila wing and haltere development by the nuclear vestigial gene product.** *Genes Dev* 1991, **5:**2481-2495.

143. James AA, Bryant PJ: **Mutations causing pattern deficiencies and duplications in the imaginal wing disk of Drosophila melanogaster.** *Dev Biol* 1981, **85:**39-54.

144. Liu Z, Steward R, Luo L: **Drosophila Lis1 is required for neuroblast proliferation, dendritic elaboration and axonal transport.** *Nat Cell Biol* 2000, **2:**776-783.

145. O'Keefe DD, Thomas JB: **Drosophila wing development in the absence of dorsal identity.** *Development* 2001, **128:**703-710.

146. Medved V, Marden JH, Fescemyer HW, Der JP, Liu J, Mahfooz N, Popadic A: **Origin and diversification of wings: Insights from a neopteran insect.** *Proc Natl Acad Sci U S A* 2015, **112:**15946-15951.

147. Ewen-Campen B, Jones TEM, Extavour CG: **Evidence against a germ plasm in the milkweed bug Oncopeltus fasciatus, a hemimetabolous insect.** *Biol Open* 2013, **2:**556-568.

148. Bickel RD, Cleveland HC, Barkas J, Jeschke CC, Raz AA, Stern DL, Davis GK: **The pea aphid uses a version of the terminal system during oviparous, but not viviparous, development.** *Evodevo* 2013, **4:**10.

149. Lu HL, Tanguy S, Rispe C, Gauthier JP, Walsh T, Gordon K, Edwards O, Tagu D, Chang CC, Jaubert-Possamai S: **Expansion of genes encoding piRNA-associated argonaute proteins in the pea aphid: diversification of expression profiles in different plastic morphs.** *PLoS One* 2011, **6:**e28051.

150. Ewen-Campen B, Jones TE, Extavour CG: **Evidence against a germ plasm in the milkweed bug *Oncopeltus fasciatus*, a hemimetabolous insect.** *Biol Open* 2013, **2:**556-568.

151. Bao R, Friedrich M: **Molecular evolution of the *Drosophila* retinome: exceptional gene gain in the higher Diptera.** *Mol Biol Evol* 2009, **26:**1273-1287.

152. Dilda CL, Mackay TF: **The genetic architecture of *Drosophila* sensory bristle number.** *Genetics* 2002, **162:**1655-1674.

153. Norga KK, Gurganus MC, Dilda CL, Yamamoto A, Lyman RF, Patel PH, Rubin GM, Hoskins RA, Mackay TF, Bellen HJ: **Quantitative analysis of bristle number in *Drosophila* mutants identifies genes involved in neural development.** *Curr Biol* 2003, **13:**1388-1396.

154. Hartenstein V, Wodarz A: **Initial neurogenesis in *Drosophila*.** *Wiley Interdiscip Rev Dev Biol* 2013, **2:**701-721.

155. Tilney LG, Connelly P, Smith S, Guild GM: **F-actin bundles in *Drosophila* bristles are assembled from modules composed of short filaments.** *J Cell Biol* 1996, **135:**1291-1308.

156. Simpson P: **The stars and stripes of animal bodies: evolution of regulatory elements mediating pigment and bristle patterns in Drosophila.** *Trends Genet* 2007, **23:**350-358.

157. Marcellini S, Simpson P: **Two or four bristles: functional evolution of an enhancer of scute in Drosophilidae.** *PLoS Biol* 2006, **4:**e386.

158. Negre B, Simpson P: **Evolution of the achaete-scute complex in insects: convergent duplication of proneural genes.** *Trends Genet* 2009, **25:**147-152.

159. Fyrberg EA, Bond BJ, Hershey ND, Mixter KS, Davidson N: **The actin genes of *Drosophila*: protein coding regions are highly conserved but intron positions are not.** *Cell* 1981, **24:**107-116.

160. Lawrence PA: **Mitosis and the cell cycle in the metamorphic moult of the milkweed bug Oncopeltus fasciatus; a radioautographic study.** *J Cell Sci* 1968, **3:**391-404.

161. Lawrence PA: **Cellular differentiation and pattern formation during metamorphosis of the milkweed bug Oncopeltus.** *Dev Biol* 1969, **19:**12-40.

162. Riddiford LM: **Prevention of Metamorphosis by Exposure of Insect Eggs to Juvenile Hormone Analogs.** *Science* 1970, **167:**287-&.

163. Willis JH, Lawrence PA: **Deferred Action of Juvenile Hormone.** *Nature* 1970, **225:**81-83.

164. Masner P, Bowers WS, Kalin M, Muhle T: **Effect of precocene II on the endocrine regulation of development and reproduction in the bug, *Oncopeltus fasciatus*.** *Gen Comp Endocrinol* 1979, **37:**156-166.

165. Erezyilmaz DF, Riddiford LM, Truman JW: **The pupal specifier broad directs progressive morphogenesis in a direct-developing insect.** *Proceedings of the National Academy of Sciences of the United States of America* 2006, **103:**6925-6930.

166. Gilbert LI: **Halloween genes encode P450 enzymes that mediate steroid hormone biosynthesis in Drosophila melanogaster.** *Mol Cell Endocrinol* 2004, **215:**1-10.

167. Rewitz K, Rybczynski R, Warren J, Gilbert L: **Developmental expression of Manduca shade, the P450 mediating the final step in molting hormone synthesis.** *Mol Cell Endocrinol* 2006, **247:**166-174.

168. Huet F, Ruiz C, Richards G: **Sequential gene activation by ecdysone in Drosophila melanogaster: the hierarchical equivalence of early and early late genes.** *Development* 1995, **121:**1195-1204.

169. Ureña E, Manjón D, Franch-Marro X, Martin D: **Transcription factor E93 specifies adult metamorphosis in hemimetabolous and holometabolous insects. .** *Proc Natl Acad Sci U S A* 2014, **111:**7024-7029.

170. Lam GT, Jiang C, Thummel CS: **Coordination of larval and prepupal gene expression by the DHR3 orphan receptor during Drosophila metamorphosis.** *Development* 1997, **124:**1757-1769.

171. Stone BL, Thummel CS: **The Drosophila 78c Early-Late Puff Contains E78, an Ecdysone-Inducible Gene That Encodes a Novel Member of the Nuclear Hormone-Receptor Superfamily.** *Cell* 1993, **75:**307-320.

172. King-Jones K, Charles J, Lam G, Thummel C: **The ecdysone-induced DHR4 orphan receptor coordinates growth and maturation in Drosophila.** *Cell* 2005, **121:**773-784.

173. Woodard C, Baehrecke E, Thummel C: **A molecular mechanism for the stage specificity of the Drosophila prepupal genetic response to ecdysone.** *Cell* 1994, **79:**607-615.

174. Baker K, Shewchuk L, Kozlova T, Makishima M, Hassell A, Wisely B, Caravella J, Lambert M, Reinking J, Krause H, et al: **The Drosophila orphan nuclear receptor DHR38 mediates an atypical ecdysteroid signaling pathway.** *Cell* 2003, **113:**731-742.

175. Truman J, Rountree D, Reiss S, Schwartz L: **Ecdysteroids regulate the release and action of eclosion hormone in the tobacco hornworm, Manduca sexta (L.)** *J Insect Physiol* 1983, **29:**895–900.

176. Zitnan D, Kingan TG, Hermesman JL, Adams ME: **Identification of ecdysis-triggering hormone from an epitracheal endocrine system.** *Science* 1996, **271:**88-91.

177. Ewer J, Truman JW: **Increases in cyclic 3',5'-guanosine monophosphate (cGMP) occur at ecdysis in an evolutionarily conserved crustacean cardioactive peptide-immunoreactive insect neuronal network.** *Journal of Comparative Neurology* 1996, **370:**330-341.

178. Dewey E, McNabb S, Ewer J, Kuo G, Takanishi C, Truman J, Honegger H: **Identification of the gene encoding bursicon, an insect neuropeptide responsible for cuticle sclerotization and wing spreading.** *Current Biology* 2004, **14:**1208-1213.

179. Willis JH: **Structural cuticular proteins from arthropods: Annotation, nomenclature, and sequence characteristics in the genomics era.** In *Insect Biochemistry and Molecular Biology*, vol. 40. pp. 189-204; 2010:189-204.

180. Zhu F, Gujar H, Gordon JR, Haynes KF, Potter MF, Palli SR: **Bed bugs evolved unique adaptive strategy to resist pyrethroid insecticides.** *Sci Rep* 2013, **3:**1456.

181. Ioannidou ZS, Theodoropoulou MC, Papandreou NC, Willis JH, Hamodrakas SJ: **CutProtFam-Pred: Detection and classification of putative structural cuticular proteins from sequence alone, based on profile Hidden Markov Models.** *Insect Biochemistry and Molecular Biology* 2014, **52:**51-59.

182. Willis JH, Papandreou NC, Iconomidou VA, Hamodrakas SJ: **5 Cuticular Proteins.** In *Insect Molecular Biology and Biochemistry.* Academic Press San Diego; 2012: 134-166

183. Parkash R, Aggarwal DD, Lambhod C, Singh D: **Divergence of water balance mechanisms and acclimation potential in body color morphs of Drosophila ananassae.** *J Exp Zool A Ecol Genet Physiol* 2014, **321:**13-27.

184. Wright TR: **The genetics of biogenic amine metabolism, sclerotization, and melanization in Drosophila melanogaster.** *Adv Genet* 1987, **24:**127-222.

185. Wittkopp PJ, Vaccaro K, Carroll SB: **Evolution of yellow gene regulation and pigmentation in Drosophila.** *Current Biology* 2002, **12:**1547-1556.

186. Wittkopp PJ, Carroll SB, Kopp A: **Evolution in black and white: genetic control of pigment patterns in Drosophila.** *Trends in Genetics* 2003, **19:**495-504.

187. Liu J, Lemonds TR, Marden JH, Popadic A: **A Pathway Analysis of Melanin Patterning in a Hemimetabolous Insect.** *Genetics* 2016, **203:**403-413.

188. Liu J, Lemonds TR, Popadic A: **The genetic control of aposematic black pigmentation in hemimetabolous insects: insights from Oncopeltus fasciatus.** *Evolution & Development* 2014, **16:**270-277.

189. Lawrence PA: **Some new mutants of large milkweed bug *Oncopeltus fasciatus* Dall.** *Genetical Research* 1970, **15:**347-350.

190. Cervera A, Maymó AC, Martínez-Pardo R, Garcerá MD: **Antioxidant Enzymes in *Oncopeltus fasciatus* (Heteroptera: Lygaeidae) Exposed to Cadmium.** *Environmental Entomology* 2003, **32:**705-710.

191. Benoit JB, Hansen IA, Szuter EM, Drake LL, Burnett DL, Attardo GM: **Emerging roles of aquaporins in relation to the physiology of blood-feeding arthropods.** *J Comp Physiol B* 2014, **184:**811-825.

192. Benoit JB, Lopez-Martinez G (Eds.): **Role of conventional and unconventional stress proteins during the response of insects to traumatic environmental conditions**; 2012.

193. Werck-Reichhart D, Feyereisen R: **Cytochromes P450: a success story.** *Genome Biol* 2000, **1:**REVIEWS3003.

194. Peterson JA, Graham SE: **A close family resemblance: the importance of structure in understanding cytochromes P450.** *Structure* 1998, **6:**1079-1085.

195. Shelton PM, Lawrence PA: **Structure and development of ommatidia in Oncopeltus fasciatus.** *J Embryol Exp Morphol* 1974, **32:**337-353.

196. Dudek FE: **The visual response from the compound eye of *Oncopeltus fasciatus*: effects of temperature and sensory adaptation.** *J Insect Physiol* 1975, **21:**517-528.

197. Bennett RR, Ruck P: **Spectral sensitivities of dark- and light-adapted Notonecta compound eyes.** *J Insect Physiol* 1970, **16:**83-88.

198. Bruckmoser P: **Die spektrale Empfindlichkeit einzelner Sehzellen des Rückenschwimmers Notonecta glauca L. (Heteroptera).** *Z Vgl Physiol* 1968, **59:**187-204.

199. Wakakuwa M, Stewart F, Matsumoto Y, Matsunaga S, Arikawa K: **Physiological basis of phototaxis to near-infrared light in Nephotettix cincticeps.** *J Comp Physiol A Neuroethol Sens Neural Behav Physiol* 2014, **200:**527-536.

200. Gao N, Foster RG, Hardie J: **Two opsin genes from the vetch aphid, *Megoura viciae*.** *Insect Mol Biol* 2000, **9:**197-202.

201. Brody T, Cravchik A: **Drosophila melanogaster G Protein-coupled Receptors.** *J Cell Biol* 2000, **150:**F83-F88.

202. Colbourne JK, Pfrender ME, Gilbert D, Thomas WK, Tucker A, Oakley TH, Tokishita S, Aerts A, Arnold GJ, Basu MK, et al: **The ecoresponsive genome of *Daphnia pulex*.** *Science* 2011, **331:**555-561.

203. Eriksson BJ, Fredman D, Steiner G, others: **Characterisation and localisation of the opsin protein repertoire in the brain and retinas of a spider and an onychophoran.** *BMC Evol Biol* 2013, **13:**186.

204. Hering L, Mayer G: **Analysis of the opsin repertoire in the tardigrade Hypsibius dujardini provides insights into the evolution of opsin genes in panarthropoda.** *Genome Biol Evol* 2014, **6:**2380-2391.

205. Löytynoja A, Goldman N: **webPRANK: a phylogeny-aware multiple sequence aligner with interactive alignment browser.** *BMC Bioinformatics* 2010, **11:**579.

206. Capella-Gutiérrez S, Silla-Martínez JM, Gabaldón T: **trimAl: a tool for automated alignment trimming in large-scale phylogenetic analyses.** *Bioinformatics* 2009, **25:**1972-1973.

207. Sánchez R, Serra F, Tárraga J, Medina I, Carbonell J, Pulido L, de María A, Capella-Gutíerrez S, Huerta-Cepas J, Gabaldón T, et al: **Phylemon 2.0: a suite of web-tools for molecular evolution, phylogenetics, phylogenomics and hypotheses testing.** *Nucleic Acids Res* 2011, **39:**W470-474.

208. Su CY, Menuz K, Carlson JR: **Olfactory perception: receptors, cells, and circuits.** *Cell* 2009, **139:**45-59.

209. Touhara K, Vosshall LB: **Sensing odorants and pheromones with chemosensory receptors.** *Annu Rev Physiol* 2009, **71:**307-332.

210. Jones WD, Cayirlioglu P, Kadow IG, Vosshall LB: **Two chemosensory receptors together mediate carbon dioxide detection in Drosophila.** *Nature* 2007, **445:**86-90.

211. Kwon JY, Dahanukar A, Weiss LA, Carlson JR: **The molecular basis of CO2 reception in Drosophila.** *Proc Natl Acad Sci U S A* 2007, **104:**3574-3578.

212. Lu T, Qiu YT, Wang G, Kwon JY, Rutzler M, Kwon HW, Pitts RJ, van Loon JJ, Takken W, Carlson JR, Zwiebel LJ: **Odor coding in the maxillary palp of the malaria vector mosquito Anopheles gambiae.** *Curr Biol* 2007, **17:**1533-1544.

213. Liman ER, Zhang YV, Montell C: **Peripheral coding of taste.** *Neuron* 2014, **81:**984-1000.

214. Kirkness EF, Haas BJ, Sun W, Braig HR, Perotti MA, Clark JM, Lee SH, Robertson HM, Kennedy RC, Elhaik E, et al: **Genome sequences of the human body louse and its primary endosymbiont provide insights into the permanent parasitic lifestyle.** *Proc Natl Acad Sci U S A* 2010, **107:**12168-12173.

215. Robertson HM, Wanner KW: **The chemoreceptor superfamily in the honey bee, Apis mellifera: expansion of the odorant, but not gustatory, receptor family.** *Genome Res* 2006, **16:**1395-1403.

216. Smadja C, Shi P, Butlin RK, Robertson HM: **Large gene family expansions and adaptive evolution for odorant and gustatory receptors in the pea aphid, Acyrthosiphon pisum.** *Mol Biol Evol* 2009, **26:**2073-2086.

217. Mesquita RD, Vionette-Amaral RJ, Lowenberger C, Rivera-Pomar R, Monteiro FA, Minx P, Spieth J, Carvalho AB, Panzera F, Lawson D, et al: **Genome of *Rhodnius prolixus*, an insect vector of Chagas disease, reveals unique adaptations to hematophagy and parasite infection.** *Proc Natl Acad Sci USA* 2015, **112:**14936-14941.

218. Penalva-Arana DC, Lynch M, Robertson HM: **The chemoreceptor genes of the waterflea Daphnia pulex: many Grs but no Ors.** *BMC Evol Biol* 2009, **9:**79.

219. Hoy MA, Waterhouse RM, Wu K, Estep AS, Ioannidis P, Palmer WJ, Pomerantz AF, Simao FA, Thomas J, Jiggins FM, et al: **Genome sequencing of the phytoseiid predatory mite *Metaseiulus occidentalis* reveals completely atomized Hox genes and superdynamic intron evolution.** *Genome Biol Evol* 2016, **8:**1762-1775.

220. Benton R, Vannice KS, Gomez-Diaz C, Vosshall LB: **Variant ionotropic glutamate receptors as chemosensory receptors in Drosophila.** *Cell* 2009, **136:**149-162.

221. Croset V, Rytz R, Cummins SF, Budd A, Brawand D, Kaessmann H, Gibson TJ, Benton R: **Ancient protostome origin of chemosensory ionotropic glutamate receptors and the evolution of insect taste and olfaction.** *PLoS Genet* 2010, **6:**e1001064.

222. Rytz R, Croset V, Benton R: **Ionotropic receptors (IRs): chemosensory ionotropic glutamate receptors in Drosophila and beyond.** *Insect Biochem Mol Biol* 2013, **43:**888-897.

223. Larkin MA, Blackshields G, Brown NP, Chenna R, McGettigan PA, McWilliam H, Valentin F, Wallace IM, Wilm A, Lopez R, et al: **Clustal W and Clustal X version 2.0.** *Bioinformatics* 2007, **23:**2947-2948.

224. Guindon S, Dufayard JF, Lefort V, Anisimova M, Hordijk W, Gascuel O: **New algorithms and methods to estimate maximum-likelihood phylogenies: assessing the performance of PhyML 3.0.** *Syst Biol* 2010, **59:**307-321.

225. Robertson HM: **The insect chemoreceptor superfamily in Drosophila pseudoobscura: Molecular evolution of ecologically-relevant genes over 25 million years** *J Insect Sci* 2009, **9:**18.

226. Terrapon N, Li C, Robertson HM, Ji L, Meng X, Booth W, Chen Z, Childers CP, Glastad KM, Gokhale K, et al: **Molecular traces of alternative social organization in a termite genome.** *Nat Commun* 2014, **5:**3636.

227. Miyamoto T, Amrein H: **Diverse roles for the Drosophila fructose sensor Gr43a.** *Fly (Austin)* 2014, **8:**19-25.

228. Delventhal R, Carlson JR: **Bitter taste receptors confer diverse functions to neurons.** *Elife* 2016, **5**.

229. Weiss LA, Dahanukar A, Kwon JY, Banerjee D, Carlson JR: **The molecular and cellular basis of bitter taste in Drosophila.** *Neuron* 2011, **69:**258-272.

230. Enjin A, Zaharieva EE, Frank DD, Mansourian S, Suh GS, Gallio M, Stensmyr MC: **Humidity Sensing in Drosophila.** *Curr Biol* 2016, **26:**1352-1358.

231. Knecht ZA, Silbering AF, Ni L, Klein M, Budelli G, Bell R, Abuin L, Ferrer AJ, Samuel AD, Benton R, Garrity PA: **Distinct combinations of variant ionotropic glutamate receptors mediate thermosensation and hygrosensation in Drosophila.** *Elife* 2016, **5**.

232. Ni L, Klein M, Svec KV, Budelli G, Chang EC, Ferrer AJ, Benton R, Samuel AD, Garrity PA: **The Ionotropic Receptors IR21a and IR25a mediate cool sensing in Drosophila.** *Elife* 2016, **5**.

233. Prieto-Godino LL, Rytz R, Bargeton B, Abuin L, Arguello JR, Peraro MD, Benton R: **Olfactory receptor pseudo-pseudogenes.** *Nature* 2016, **539:**93-97.

234. Silbering AF, Rytz R, Grosjean Y, Abuin L, Ramdya P, Jefferis GS, Benton R: **Complementary function and integrated wiring of the evolutionarily distinct Drosophila olfactory subsystems.** *J Neurosci* 2011, **31:**13357-13375.

235. Koh TW, He Z, Gorur-Shandilya S, Menuz K, Larter NK, Stewart S, Carlson JR: **The Drosophila IR20a clade of ionotropic receptors are candidate taste and pheromone receptors.** *Neuron* 2014, **83:**850-865.

236. Stewart S, Koh TW, Ghosh AC, Carlson JR: **Candidate ionotropic taste receptors in the Drosophila larva.** *Proc Natl Acad Sci U S A* 2015, **112:**4195-4201.

237. Bansal R, Michel AP: **Core RNAi Machinery and *Sid1*, a component for systemic RNAi, in the hemipteran insect, *Aphis glycines*.** *Int J Mol Sci* 2013, **14:**3786-3801.

238. Jaubert-Possamai S, Rispe C, Tanguy S, Gordon K, Walsh T, Edwards O, Tagu D: **Expansion of the miRNA pathway in the hemipteran insect *Acyrthosiphon pisum*.** *Mol Biol Evol* 2010, **27:**979-987.

239. LaChance LE, Richard RD: **Irradiation of sperm and oocytes in *Oncopeltus fasciatus* (Hemiptera: Lygaeidae): sex ratio, fertility, and chromosome aberrations in the F1 progeny.** *Can J Genet Cytol* 1973, **15:**713-721.

240. Shukla JN, Nagaraju J: **Doublesex: a conserved downstream gene controlled by diverse upstream regulators.** *J Genet* 2010, **89:**341-356.

241. Geuverink E, Beukeboom LW: **Phylogenetic distribution and evolutionary dynamics of the sex determination genes doublesex and transformer in insects.** *Sex Dev* 2014, **8:**38-49.

242. An W, Cho S, Ishii H, Wensink PC: **Sex-specific and non-sex-specific oligomerization domains in both of the doublesex transcription factors from Drosophila melanogaster.** *Mol Cell Biol* 1996, **16:**3106-3111.

243. Biewer M, Schlesinger F, Hasselmann M: **The evolutionary dynamics of major regulators for sexual development among Hymenoptera species.** *Front Genet* 2015, **6:**124.

244. Shukla JN, Palli SR: **Doublesex target genes in the red flour beetle, Tribolium castaneum.** *Sci Rep* 2012, **2:**948.

245. Sarno F, Ruiz MF, Eirin-Lopez JM, Perondini AL, Selivon D, Sanchez L: **The gene transformer-2 of Anastrepha fruit flies (Diptera, Tephritidae) and its evolution in insects.** *BMC Evol Biol* 2010, **10:**140.

246. Siegal ML, Baker BS: **Functional conservation and divergence of intersex, a gene required for female differentiation in *Drosophila melanogaster*.** *Dev Genes Evol* 2005, **215:**1-12.

247. Clynen E, Ciudad L, Belles X, Piulachs MD: **Conservation of fruitless' role as master regulator of male courtship behaviour from cockroaches to flies.** *Dev Genes Evol* 2011, **221:**43-48.

248. Suzuki MG, Imanishi S, Dohmae N, Nishimura T, Shimada T, Matsumoto S: **Establishment of a novel in vivo sex-specific splicing assay system to identify a trans-acting factor that negatively regulates splicing of *Bombyx mori* dsx female exons.** *Mol Cell Biol* 2008, **28:**333-343.

249. Conrad T, Cavalli FM, Vaquerizas JM, Luscombe NM, Akhtar A: **Drosophila dosage compensation involves enhanced Pol II recruitment to male X-linked promoters.** *Science* 2012, **337:**742-746.

250. Verhulst EC, van de Zande L, Beukeboom LW: **Insect sex determination: it all evolves around transformer.** *Curr Opin Genet Dev* 2010, **20:**376-383.

251. Gempe T, Beye M: **Function and evolution of sex determination mechanisms, genes and pathways in insects.** *Bioessays* 2011, **33:**52-60.

252. Duncan EJ, Gluckman PD, Dearden PK: **Epigenetics, plasticity, and evolution: How do we link epigenetic change to phenotype?** *J Exp Zool B Mol Dev Evol* 2014, **322:**208-220.

253. Jones PA: **Functions of DNA methylation: islands, start sites, gene bodies and beyond.** *Nat Rev Genet* 2012, **13:**484-492.

254. Foret S, Kucharski R, Pellegrini M, Feng S, Jacobsen SE, Robinson GE, Maleszka R: **DNA methylation dynamics, metabolic fluxes, gene splicing, and alternative phenotypes in honey bees.** *Proc Natl Acad Sci U S A* 2012, **109:**4968-4973.

255. Lyko F, Foret S, Kucharski R, Wolf S, Falckenhayn C, Maleszka R: **The honey bee epigenomes: differential methylation of brain DNA in queens and workers.** *PLoS Biol* 2010, **8:**e1000506.

256. Sati S, Tanwar VS, Kumar KA, Patowary A, Jain V, Ghosh S, Ahmad S, Singh M, Reddy SU, Chandak GR, et al: **High resolution methylome map of rat indicates role of intragenic DNA methylation in identification of coding region.** *PLoS One* 2012, **7:**e31621.

257. Herb BR, Wolschin F, Hansen KD, Aryee MJ, Langmead B, Irizarry R, Amdam GV, Feinberg AP: **Reversible switching between epigenetic states in honeybee behavioral subcastes.** *Nat Neurosci* 2012, **15:**1371-1373.

258. Wang Y, Jorda M, Jones PL, Maleszka R, Ling X, Robertson HM, Mizzen CA, Peinado MA, Robinson GE: **Functional CpG methylation system in a social insect.** *Science* 2006, **314:**645-647.

259. Walsh TK, Brisson JA, Robertson HM, Gordon K, Jaubert-Possamai S, Tagu D, Edwards OR: **A functional DNA methylation system in the pea aphid, Acyrthosiphon pisum.** *Insect Mol Biol* 2010, **19 Suppl 2:**215-228.

260. Guo JU, Su Y, Zhong C, Ming GL, Song H: **Hydroxylation of 5-methylcytosine by TET1 promotes active DNA demethylation in the adult brain.** *Cell* 2011, **145:**423-434.

261. Tahiliani M, Koh KP, Shen Y, Pastor WA, Bandukwala H, Brudno Y, Agarwal S, Iyer LM, Liu DR, Aravind L, Rao A: **Conversion of 5-methylcytosine to 5-hydroxymethylcytosine in mammalian DNA by MLL partner TET1.** *Science* 2009, **324:**930-935.

262. Feng S, Cokus SJ, Zhang X, Chen PY, Bostick M, Goll MG, Hetzel J, Jain J, Strauss SH, Halpern ME, et al: **Conservation and divergence of methylation patterning in plants and animals.** *Proc Natl Acad Sci U S A* 2010, **107:**8689-8694.

263. Suzuki MM, Kerr AR, De Sousa D, Bird A: **CpG methylation is targeted to transcription units in an invertebrate genome.** *Genome Res* 2007, **17:**625-631.

264. Zemach A, McDaniel IE, Silva P, Zilberman D: **Genome-wide evolutionary analysis of eukaryotic DNA methylation.** *Science* 2010, **328:**916-919.

265. Kass SU, Landsberger N, Wolffe AP: **DNA methylation directs a time-dependent repression of transcription initiation.** *Curr Biol* 1997, **7:**157-165.

266. Laurent L, Wong E, Li G, Huynh T, Tsirigos A, Ong CT, Low HM, Kin Sung KW, Rigoutsos I, Loring J, Wei CL: **Dynamic changes in the human methylome during differentiation.** *Genome Res* 2010, **20:**320-331.

267. Elango N, Hunt BG, Goodisman MA, Yi SV: **DNA methylation is widespread and associated with differential gene expression in castes of the honeybee, Apis mellifera.** *Proc Natl Acad Sci U S A* 2009, **106:**11206-11211.

268. Chipman AD, Ferrier DEK, Brena C, Qu J, Hughes DST, Schröder R, Torres-Oliva M, Znassi N, Jiang H, Almeida FC, et al: **The first myriapod genome sequence reveals conservative arthropod gene content and genome organisation in the centipede *Strigamia maritima*.** *PLoS Biol* 2014, **12:**e1002005.

269. Fraley C, Raftery AE: **Enhanced model-based clustering, density estimation, and discriminant analysis software: MCLUST.** *Journal of Classification* 2003, **20:**263-286.

270. Talbert PB, Henikoff S: **Environmental responses mediated by histone variants.** *Trends Cell Biol* 2014, **24:**642-650.

271. Rider SD, Srinivasan DG, Hilgarth RS: **Chromatin-remodelling proteins of the pea aphid, *Acyrthosiphon pisum* (Harris).** *Insect Molecular Biology* 2010, **19:**201-214.

272. Garcia-Perez JL, Widmann TJ, Adams IR: **The impact of transposable elements on mammalian development.** *Development* 2016, **143:**4101-4114.

273. Kriventseva EV, Tegenfeldt F, Petty TJ, Waterhouse RM, Simão FA, Pozdnyakov IA, Ioannidis P, Zdobnov EM: **OrthoDB v8: update of the hierarchical catalog of orthologs and the underlying free software.** *Nucl Acids Res* 2015, **43:**D250-D256.

274. Ioannidis P, Simao FA, Waterhouse RM, Manni M, Seppey M, Robertson HM, Misof B, Niehuis O, Zdobnov EM: **Genomic features of the damselfly *Calopteryx splendens* representing a sister clade to most insect orders.** *Genome Biol Evol* 2017, **9:**415-430.

275. Zdobnov EM, Tegenfeldt F, Kuznetsov D, Waterhouse RM, Simão FA, Ioannidis P, Seppey M, Loetscher A, Kriventseva EV: **OrthoDB v9.1: cataloging evolutionary and functional annotations for animal, fungal, plant, archaeal, bacterial and viral orthologs.** *Nucleic Acids Res* 2017, **45:**D744-D749.

276. Stamatakis A: **RAxML-VI-HPC: maximum likelihood-based phylogenetic analyses with thousands of taxa and mixed models.** *Bioinformatics* 2006, **22:**2688-2690.

277. Edgar RC: **MUSCLE: multiple sequence alignment with high accuracy and high throughput.** *Nucl Acids Res* 2004, **32:**1792-1797.

278. Rice P, Longden I, Bleasby A: **EMBOSS: The European Molecular Biology Open Software Suite.** *Trends Genet* 2000, **16:**276-277.
